# Supplementary figures and images for: The Kelch13 compartment contains highly divergent vesicle trafficking proteins in malaria parasites
Source: PLoS Pathog. 2023 Dec 1;19(12):e1011814. doi: 10.1371/journal.ppat.1011814 (PMC10718435; doi:10.1371/journal.ppat.1011814)

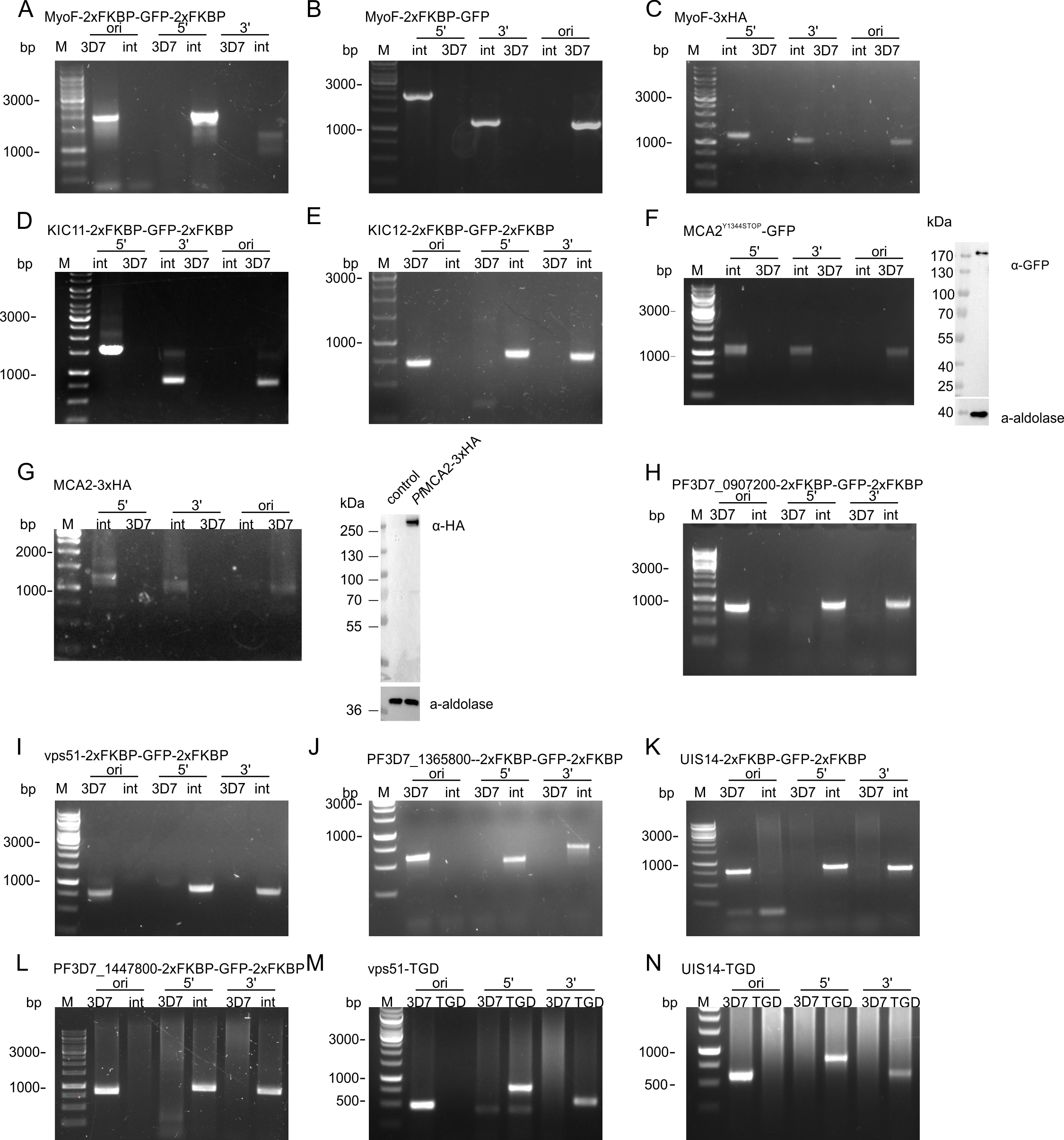

Supplement: S1 Fig — Confirmatory PCR of unmodified wildtype (WT) and transgenic knock-in (KI) / targeted-gene-disruption (TGD) cell lines to confirm correct genomic integration at the 3’- and 5’-end of the locus. Oligonucleotides used are listed in S2 Table. (A) MCA2Y1344STOP-GFPendo; (B) MCA2-3xHAendo; (C) MyoF-2xFKBP-GFP-2xFKBPendo; (D) MyoF-2xFKBP-GFPendo; (E) MyoF-3xHAendo; (F) KIC11-2xFKBP-GFP-2xFKBPendo; (G) KIC12-2xFKBP-GFP-2xFKBPendo; (H) PF3D7_0907200-2xFKBP-GFP-2xFKBPendo; (I) VPS51-2xFKBP-GFP-2xFKBPendo; (J) PF3D7_1365800-2xFKBP-GFP-2xFKBPendo; (K) UIS14-2xFKBP-GFP-2xFKBPendo; (L) PF3D7_1447800-2xFKBP-GFP-2xFKBPendo; (M) VPS51-TGDendo; (N) UIS14-TGDendo. Right panel in (F and G) Western Blot analysis of (F) MCA2Y1344STOP-GFPendo cell line using mouse anti-GFP to detect the tagged fusion protein (upper panel) and rabbit anti-aldolase to control for equal loading (lower panel)(expected molecular weight for MCA2Y1344STOP-GFP fusion proteins: 187 kDa) and (G) wildtype (3D7) and knock-in MCA2-3xHAendo cell line using mouse anti-HA to detect the tagged full-length protein (upper panel) and rabbit anti-aldolase to control for equal loading (lower panel) (expected molecular weight for MCA2-3xHA fusion protein: 281 kDa). Protein size is indicated in kDa. (TIF) [file ppat.1011814.s001.tif]

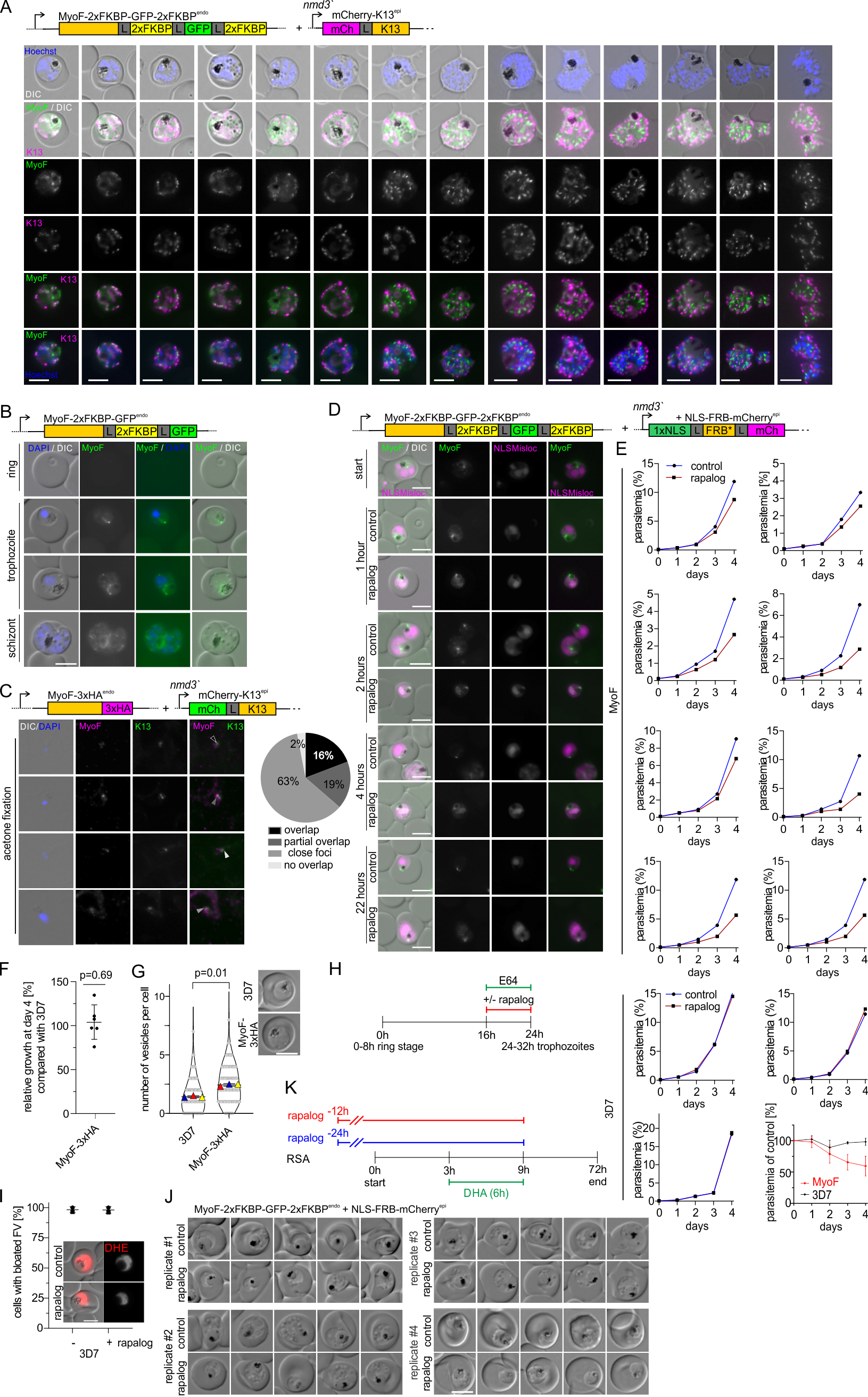

Supplement: S2 Fig — (A) Extended panel of live cell microscopy images of parasites expressing the MyoF-2xFKBP-GFP-2xFKBP fusion protein from the endogenous locus with episomally expressed mCherry-K13. Nuclei were stained with Hoechst. Scale bar, 5 μm. (B) Localisation of MyoF-2xFKBP-GFP by live-cell microscopy across the intra-erythrocytic development cycle. Nuclei were stained with DAPI. Scale bar, 5 μm. (C) IFA microscopy images of acetone-fixed parasites expressing MyoF-3xHA with episomally expressed mCherry-K13 across the intra-erythrocytic development cycle. Nuclei were stained with DAPI. Scale bar, 5μm. Foci were categorized into ‘overlap’ (black), ‘partial overlap’ (dark grey), close foci (light blue) and ‘non overlap’ (light grey) in n = 31 parasites. Scale bar, 5μm. (D) Live-cell microscopy of knock sideways (+ rapalog) and control (without rapalog) MyoF-2xFKBP-GFP-2xFKBPendo+1xNLSmislocaliser parasites at 0, 1, 2, 4 and 22 hours after the induction of knock-sideways by addition of rapalog. (E) Individual growth curves of MyoF-2xFKBP-GFP-2xFKBPendo+1xNLSmislocaliser with (red) or without (blue) addition of rapalog shown in Fig 1F. Summary of individual growth curves shown as percentage of control parasites (without rapalog). Three (3D7, black) and eight (MyoF-2xFKBP-GFP-2xFKBPendo, red) independent experiments. Error bars, mean ± SD. (F) Relative growth of synchronised MyoF-3xHAendo compared with 3D7 wild type parasites after two growth cycles. Each dot shows one of six independent growth experiments. P-values determined by one-sample t-test. (G) Number of vesicles per parasite in trophozoites determined by live-cell fluorescence microscopy (DIC) in 3D7 and MyoF-3xHAendo parasites. Three independent experiments with each time n = 27–38 (mean 31.7) parasites analysed per condition. Representative DIC images shown on the right. (H) Experimental setup of the bloated food vacuole assay shown in Fig 1H. (I) Bloated food vacuole assay with 3D7 parasites 8 hours after rapalog additio [file ppat.1011814.s002.tif]

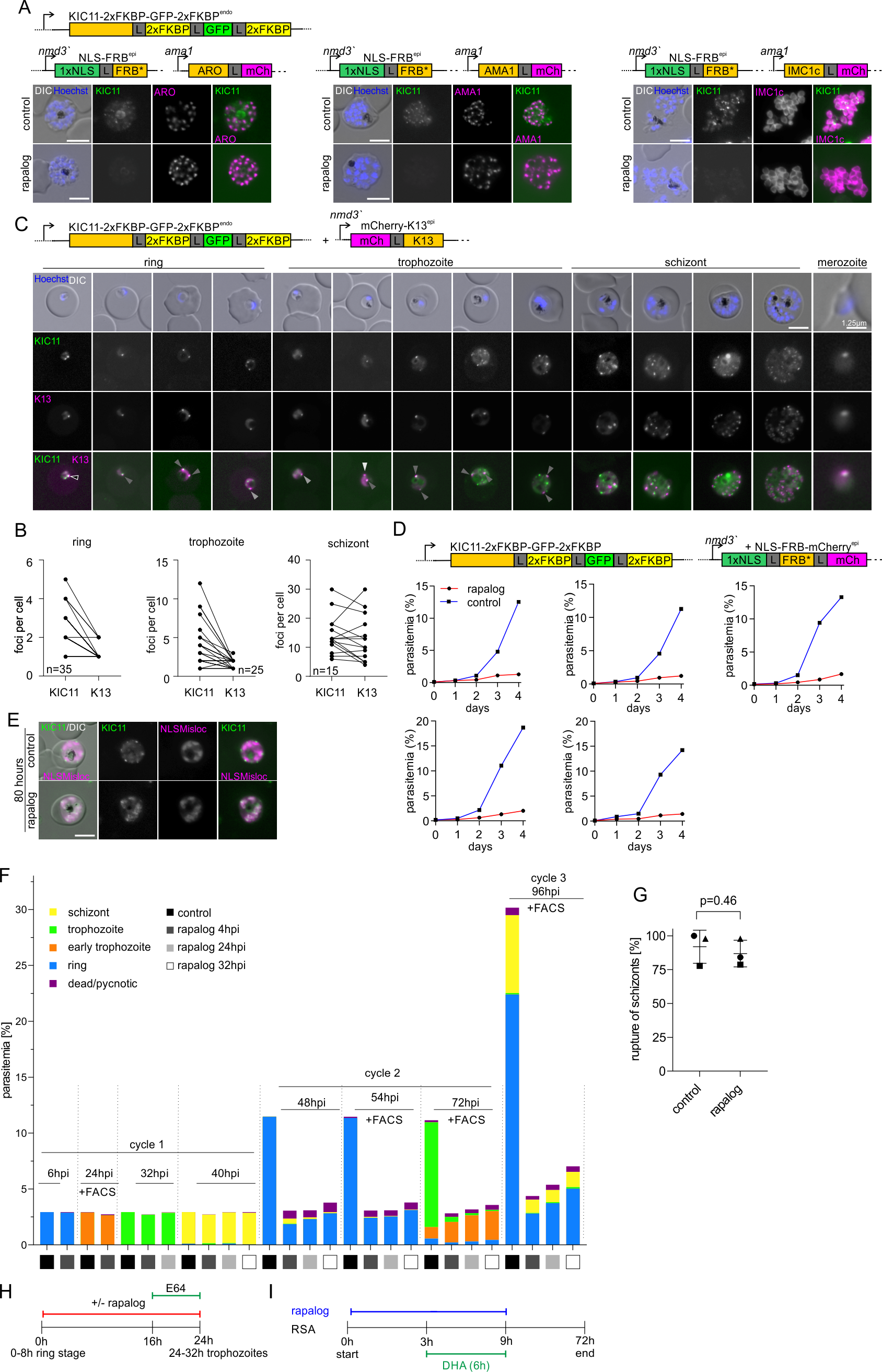

Supplement: S3 Fig — (A) Live-cell microscopy of knock sideways (+ rapalog) and control (without rapalog) KIC11-2xFKBP-GFP-2xFKBPendo ama1IMC1c-mSca_nmd3’-NLS-FRB-T2A-DHODH, KIC11-2xFKBP-GFP-2xFKBPendo+ ama1ARO-mSca_nmd3’-NLS-FRB-T2A-DHODH or KIC11-2xFKBP-GFP-2xFKBPendo+ ama1AMA1-mSca_nmd3’-NLS-FRB-T2A-DHODH parasites 40 hours after the induction of knock-sideways by addition of rapalog. Nuclei were stained with Hoechst; scale bar, 5 μm. (B) Quantification of KIC11 and K13 foci per cell in ring, trophozoite and schizont stage KIC11-2xFKBP-GFP-2xFKBPendo with episomally expressed mCherry-K13 parasites. N indicates the number of analysed KIC11-2xFKBP-GFP-2xFKBP parasites with episomally expressed mCherry-K13 per stage. (C) Extended panel of live cell microscopy images of parasites expressing KIC11-2xFKBP-GFP-2xFKBPendo with episomally expressed mCherry-K13 shown in Fig 2B. Nuclei were stained with DAPI. Scale bar, 5 μm except last column (zoom merozoite image): Scale bar = 1.25μm. Arrows are indicating categories from Fig 2C (D) Individual growth curves of knock sideways (+ rapalog) and control (without rapalog) of KIC11-2xFKBP-GFP-2xFKBPendo+1xNLSmislocaliser shown in Fig 2E. (E) Live-cell microscopy of knock sideways (+ rapalog) and control (without rapalog) KIC11-2xFKBP-GFP-2xFKBPendo+1xNLSmislocaliser parasites 80 hours after the induction of knock-sideways by addition of rapalog. Scale bar, 5 μm. (F) Parasite stage distribution in Giemsa smears at the time points (average hours post invasion, h) indicated above each bar in tightly synchronised (±4h) KIC11-2xFKBP-GFP-2xFKBPendo+1xNLSmislocaliser parasites (rapalog addition at 4 hpi, 20 hpi, or 32 hpi and control) parasite cultures over two consecutive cycles (last time point in cycle 3). A second replicate is shown in Fig 2F. (G) Quantification of ruptured schizonts at ‘post-egress’ time point compared to ‘pre-egress’ time point in knock-sideways (+rapalog) compared to control (-rapalog) in KIC11-2xFKBP-GFP-2xFKBPendo+1xNLSmislocalise [file ppat.1011814.s003.tif]

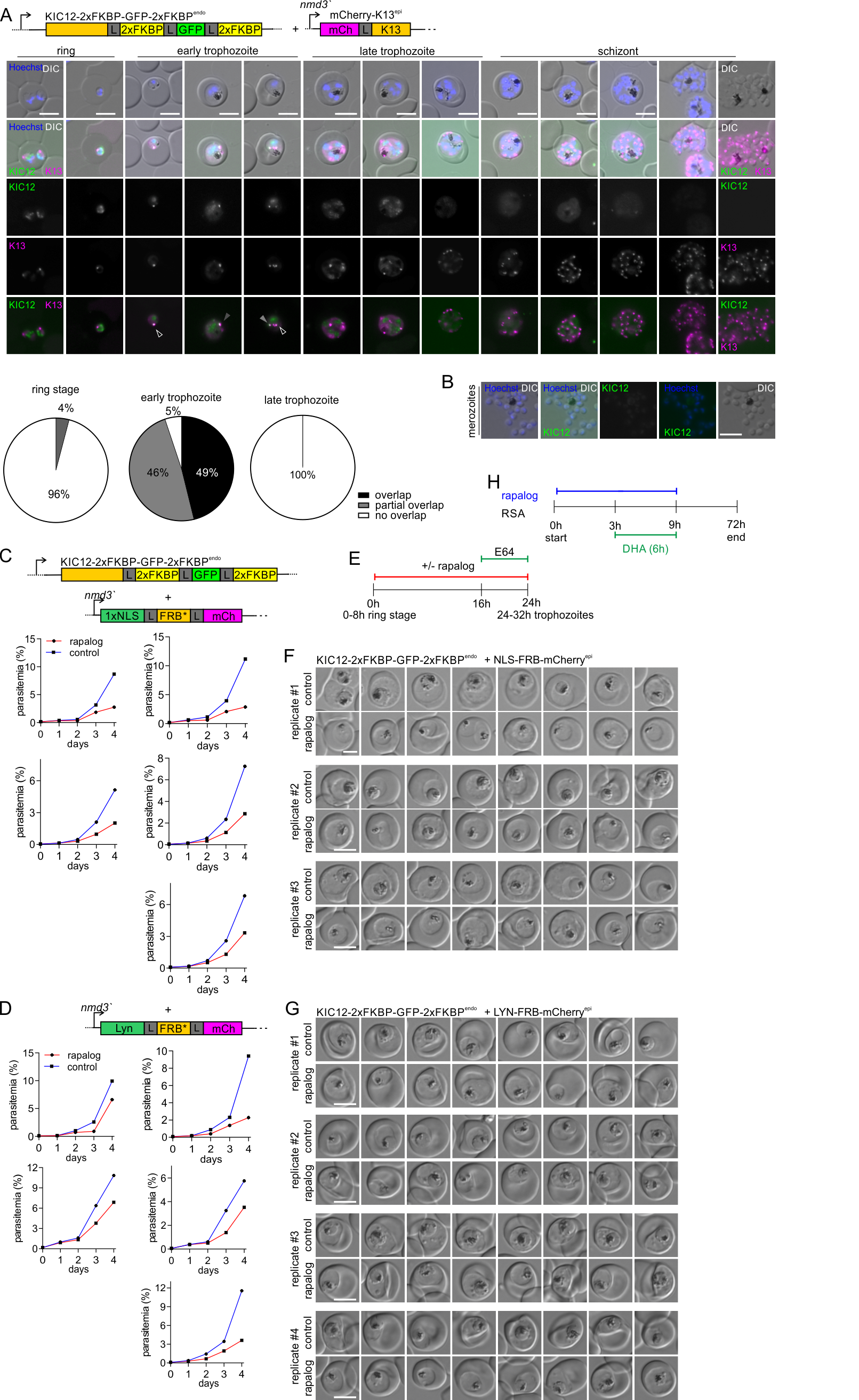

Supplement: S4 Fig — (A) Extended panel of KIC12-2xFKBP-GFP-2xFKBPendo localisation by live-cell microscopy across the intra-erythrocytic development cycle. Nuclei were stained with Hoechst. Scale bar, 5 μm. Expanded panel of Fig 3B Foci were categorized into ‘overlap’ (black), ‘partial overlap’ (grey) and ‘no overlap’ (white) and shown as frequencies in the pie chart (n = 97 cells were scored from a total of five independent experiments). (B) Absence of KIC12-2xFKBP-GFP-2xFKBPendo in free merozoites by live-cell microscopy. Nuclei were stained with Hoechst. Scale bar, 5 μm. (C) Individual growth curves of knock sideways (+ rapalog; red) and control (without rapalog; blue) KIC12-2xFKBP-GFP-2xFKBPendo+1xNLSmislocaliser shown in Fig 3D. (D) Individual growth curves of knock sideways (+ rapalog; red) and control (without rapalog; blue) KIC12-2xFKBP-GFP-2xFKBPendo+LYNmislocaliser shown in Fig 3D. (E) Experimental setup of the bloated food vacuole assay shown in Fig 3F. (F) Representative images of bloated food vacuole assay knock sideways (+ rapalog; bottom row) and control (without rapalog; top row) KIC12-2xFKBP-GFP-2xFKBPendo+1xNLSmislocaliser Scale bar, 5 μm. (G) Representative images of knock sideways (+ rapalog, top row) and control (without rapalog; bottom row) KIC12-2xFKBP-GFP-2xFKBPendo+LYNmislocaliser parasites from bloated food vacuole assay. Scale bar, 5 μm. (H) Experimental setup of the RSA shown in Fig 3L. Schematic representation of the cell lines depicted above the corresponding panel. (TIF) [file ppat.1011814.s004.tif]

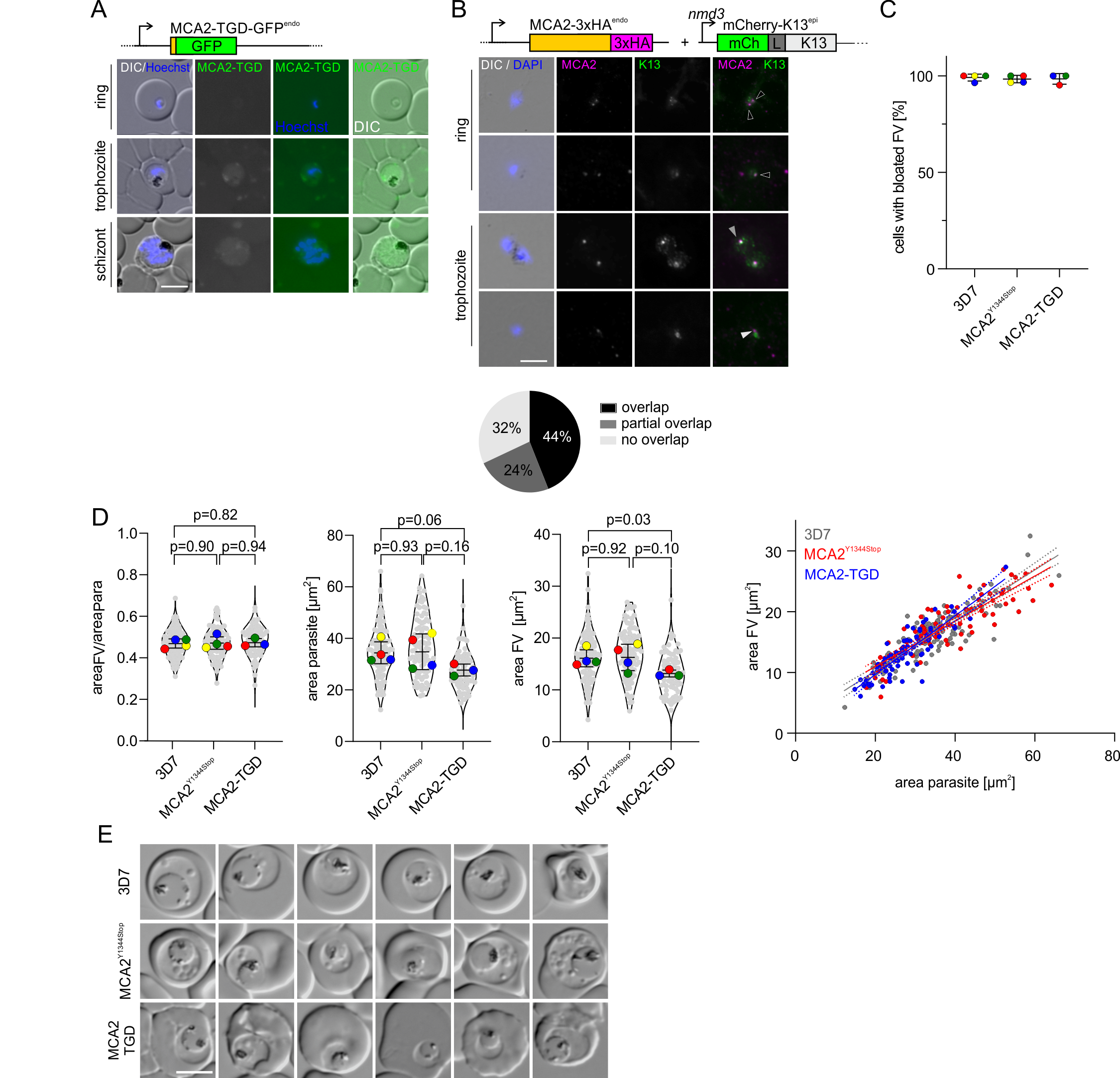

Supplement: S5 Fig — (A) Localisation of MCA2-TGD-GFP by live-cell microscopy across the intra-erythrocytic development cycle. Nuclei were stained with DAPI. Scale bar, 5 μm. (B) IFA microscopy images of acetone-fixed parasites expressing MCA2-3xHA with episomally expressed mCherry-K13 across the intra-erythrocytic development cycle. Nuclei were stained with DAPI. Scale bar, 5μm. Foci were categorized into ‘overlap’ (black), ‘partial overlap’ (dark grey) and ‘no overlap’ (light grey) in n = 30 parasites. Schematic representation of the cell lines depicted above the corresponding panel. (C) Bloated food vacuole assay with 3D7, MCA2Y1344STOP and MCA2-TGD parasites. Cells were categorized as with ‘bloated FV’ or ‘non-bloated FV’ and percentage of cells with bloated FV is displayed; n = 4 (3D7, MCA2Y1344STOP) or n = 3 (MCA2-TGD) independent experiments with each n = 10–34 (mean 25.8) parasites analysed per condition. (D) Area of the FV, area of the parasite and area of FV divided by area of the corresponding parasites were determined. Mean of each independent experiment indicated by coloured symbols, individual data points by grey dots. Data presented according to SuperPlot guidelines [147]; Error bars represent mean ± SD. P-value determined by un-paired t-test. Area of FV of individual cells plotted versus the area of the corresponding parasite. Line represents linear regression with error indicated by dashed line. (E) Representative DIC images from S2C and S2D Fig are displayed. Schematic representation of the cell lines depicted above the corresponding panel. (TIF) [file ppat.1011814.s005.tif]

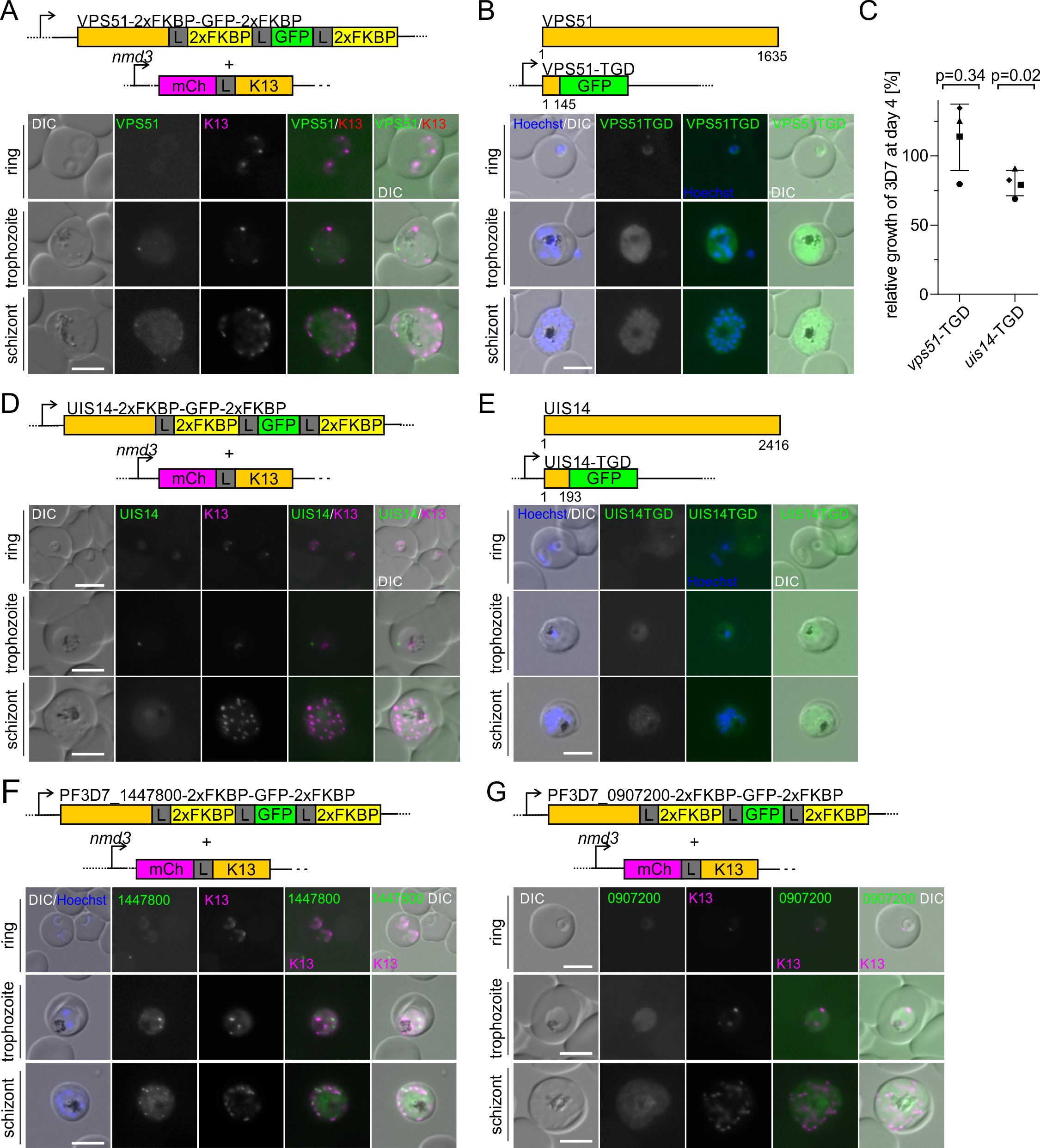

Supplement: S6 Fig — (A) Live cell microscopy images of parasites endogenously expressing VPS51-2xFKBP-GFP-2xFKBP with episomally expressed mCherry-K13 across the intra-erythrocytic development cycle. Scale bar, 5 μm. (B) Localisation of truncated VPS51TGD-GFP fusion protein by live-cell microscopy across the intra-erythrocytic development cycle. Nuclei were stained with DAPI. Scale bar, 5 μm. Schematic representation of the truncation strategy depicted above the panel, numbers indicating AA. (C) Relative growth of VPS51TGD and UIS14TGD parasites compared to 3D7 wild type parasites after two cycles. Four independent growth experiments. P-values determined by one-sample t-test. (D) Live cell microscopy images of parasites endogenously expressing UIS14-2xFKBP-GFP-2xFKBP with episomally expressed mCherry-K13 across the intra-erythrocytic development cycle. Scale bar, 5 μm. (E) Localisation of truncated UIS14TGD-GFP fusion protein by live-cell microscopy across the intra-erythrocytic development cycle. Nuclei were stained with DAPI. Scale bar, 5 μm. Schematic representation of the truncation strategy depicted above the panel, numbers indicating AA. (F) Live cell microscopy images of parasites endogenously expressing PF3D7_1447800-2xFKBP-GFP-2xFKBP with episomally expressed mCherry-K13 across the intra-erythrocytic development cycle. Nuclei were stained with DAPI. Scale bar, 5 μm. (G) Live cell microscopy images of parasites endogenously expressing PF3D7_0907200-2xFKBP-GFP-2xFKBP with episomally expressed mCherry-K13 across the intra-erythrocytic development cycle. Scale bar, 5 μm. Schematic representation of relevant features of each cell line depicted above the corresponding panel. (TIF) [file ppat.1011814.s006.tif]

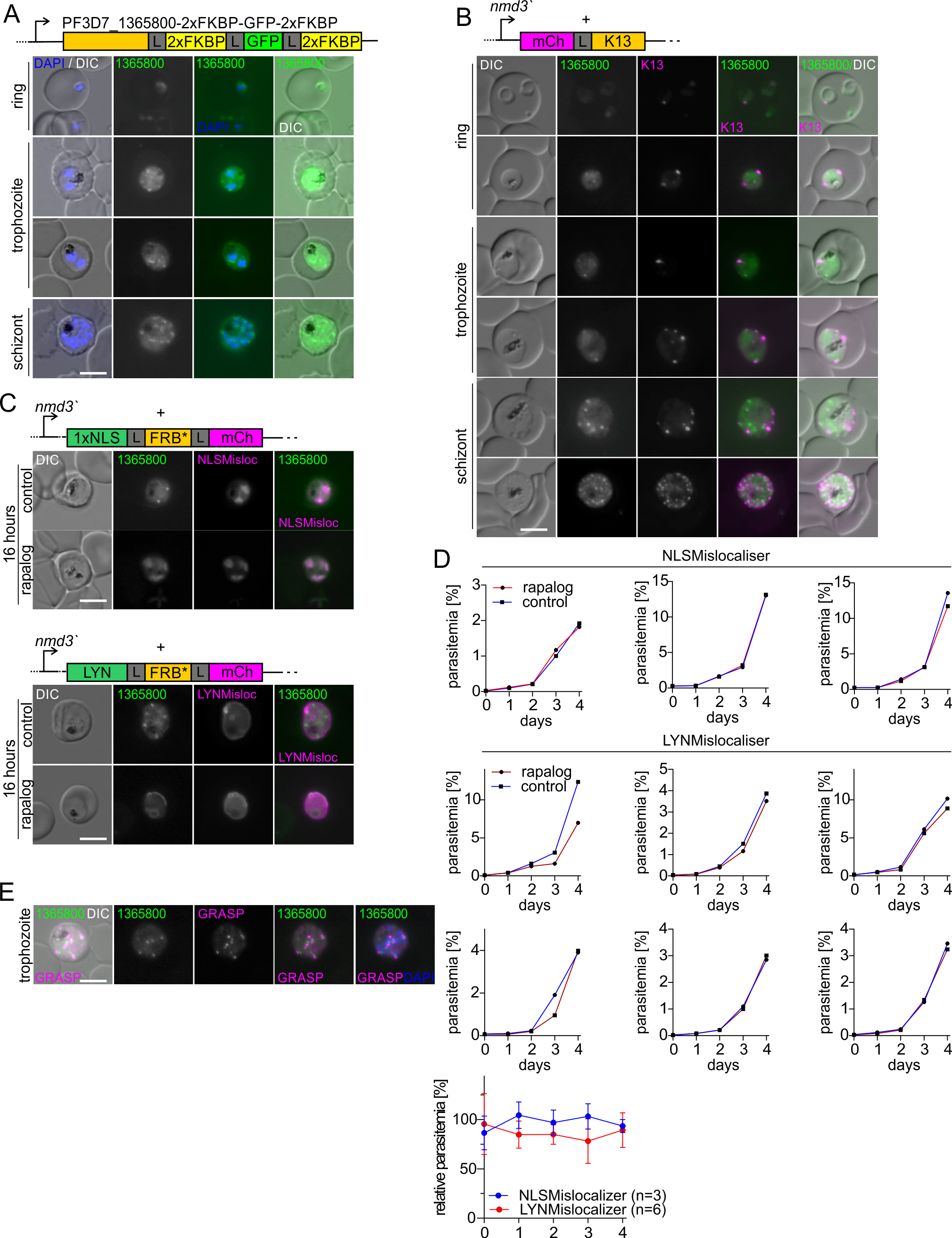

Supplement: S7 Fig — (A) Live cell microscopy images of parasites endogenously expressing PF3D7_1365800-2xFKBP-GFP-2xFKBP by live-cell microscopy across the intra-erythrocytic development cycle. Nuclei were stained with DAPI. Scale bar, 5 μm. (B) Expression of PF3D7_1365800-2xFKBP-GFP-2xFKBP with episomally expressed mCherry-K13. Scale bar, 5 μm. (C) Live-cell microscopy of knock sideways (+ rapalog) and control (without rapalog) PF3D7_1365800-2xFKBP-GFP-2xFKBPendo+1xNLSmislocaliser parasites or PF3D7_1365800-2xFKBP-GFP-2xFKBPendo+LYNmislocaliser parasites 16 hours after the induction of knock-sideways by addition of rapalog. Scale bar, 5 μm. (D) Individual growth curves of PF3D7_1365800-2xFKBP-GFP-2xFKBPendo+1xNLSmislocaliser or PF3D7_1365800-2xFKBP-GFP-2xFKBPendo+LYNmislocaliser parasites with (red) or without (blue) addition of rapalog. Relative growth of knock sideways (+ rapalog) compared to control (without rapalog) PF3D7_1365800-2xFKBP-GFP-2xFKBPendo+1xNLSmislocaliser (blue) or PF3D7_1365800-2xFKBP-GFP-2xFKBPendo+LYNmislocaliser (red) parasites over five days. Three (NLSmisloc) or six (LYNmisloc) independent growth experiments were performed and mean relative parasitemia ± SD is shown. (E) Live cell microscopy images of parasites expressing PF3D7_1365800-2xFKBP-GFP-2xFKBP with episomally expressed Golgi marker GRASP-mCherry. Schematic representation of the cell lines depicted above the corresponding panel. Scale bar, 5 μm. (TIF) [file ppat.1011814.s007.tif]

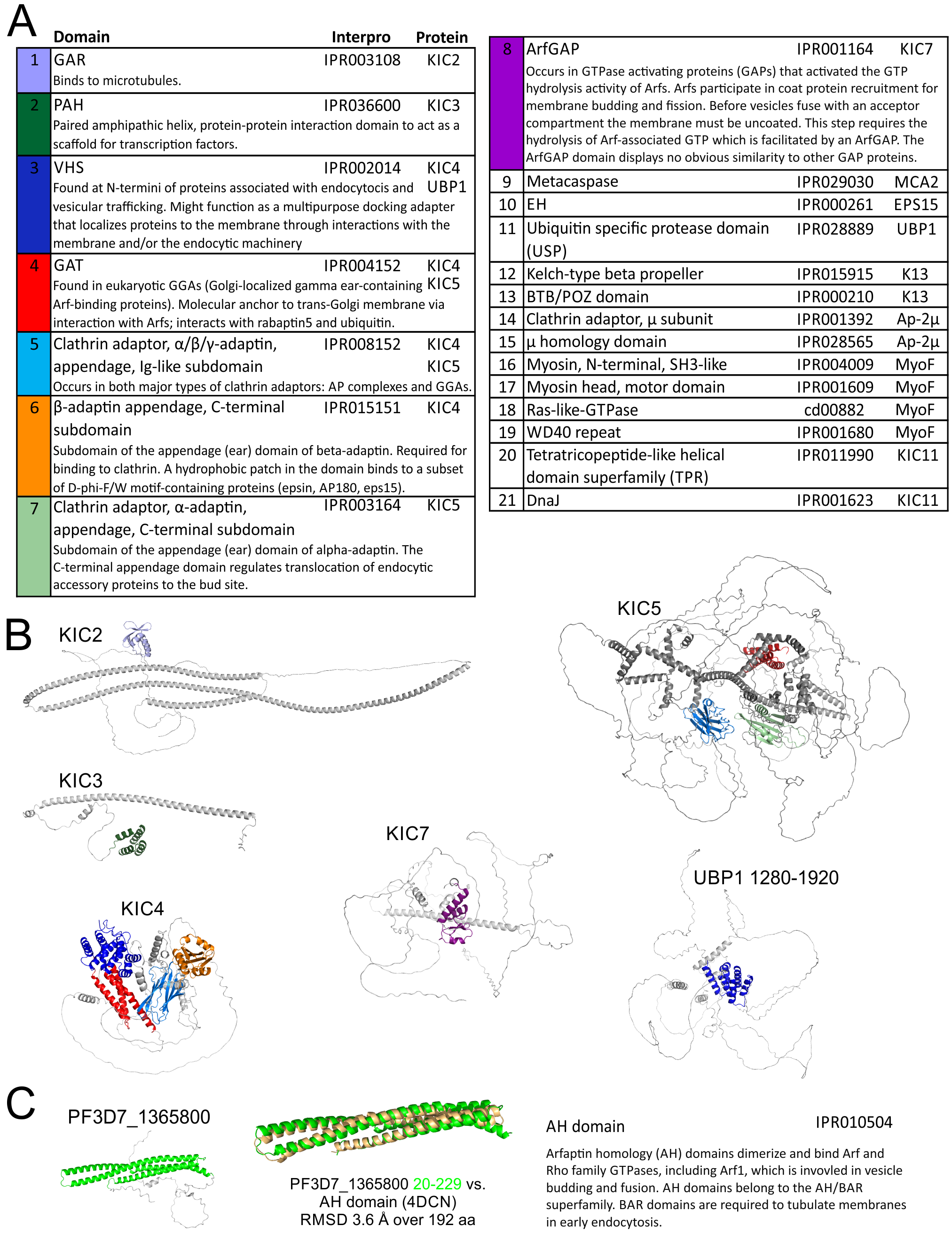

Supplement: S8 Fig — (A) Full domain names and Interpro domain numbers for each domain in Fig 5 with indication in which K13-compartment members these proteins occur. For each newly identified domain a brief summary of its reported function is given. Colours are as in Fig 5. (B) Full length AlphaFold predictions for each K13-compartment member in which new domains were identified. New domains are coloured as in A and Fig 5. For UBP1 no prediction was available in the EMBL-AlphaFold database, and the structure was predicted in fragments as described in the methods. The predicted fragment containing the newly identified domain is shown. (C) Full length structure of PF3D7_1365800, with AH domain in green. PF3D7_1365800 AH domain in green aligned with the most similar domain from the PDB. PDB ID and alignment details are indicated beneath each set of aligned domains. Brief summary about the AH domain and its Interpro ID are given. (TIF) [file ppat.1011814.s008.tif]

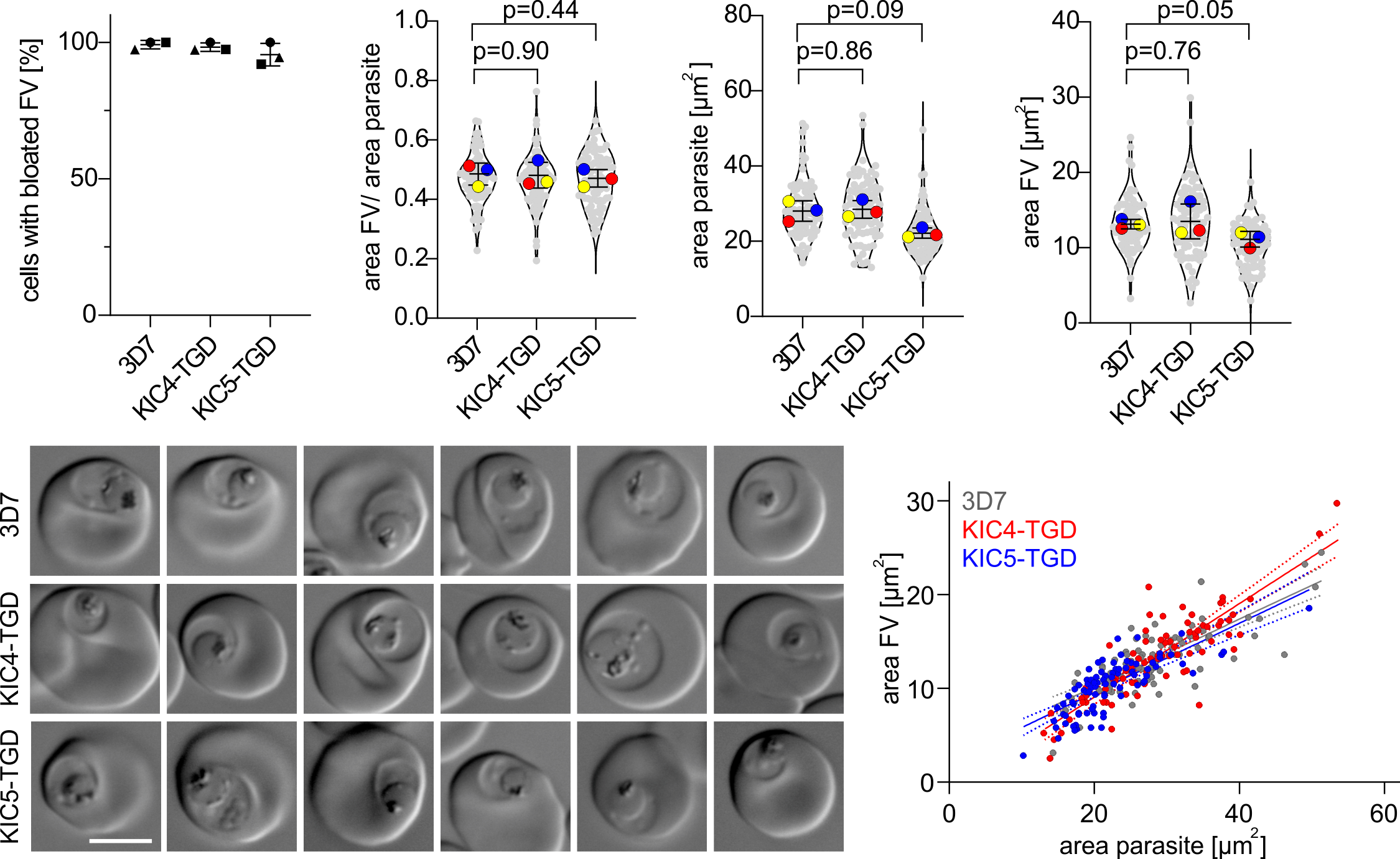

Supplement: S9 Fig — Bloated food vacuole assay with 3D7, KIC4-TGD and KIC5-TGD parasites. Cells were categorized as with ‘bloated FV’ or ‘non-bloated FV’ and percentage of cells with bloated FV is displayed; n = 3 independent experiments with each n = 20–38 (mean 26.7) parasites analysed per condition. Representative DIC are displayed. Area of the FV, area of the parasite and area of FV divided by area of the corresponding parasites were determined. Mean of each independent experiment indicated by coloured symbols, individual data points by grey dots. Data presented according to SuperPlot guidelines [147]; Error bars represent mean ± SD. P-value determined by paired t-test. Area of FV of individual cells plotted versus the area of the corresponding parasite. Line represents linear regression with error indicated by dashed line. (TIF) [file ppat.1011814.s009.tif]

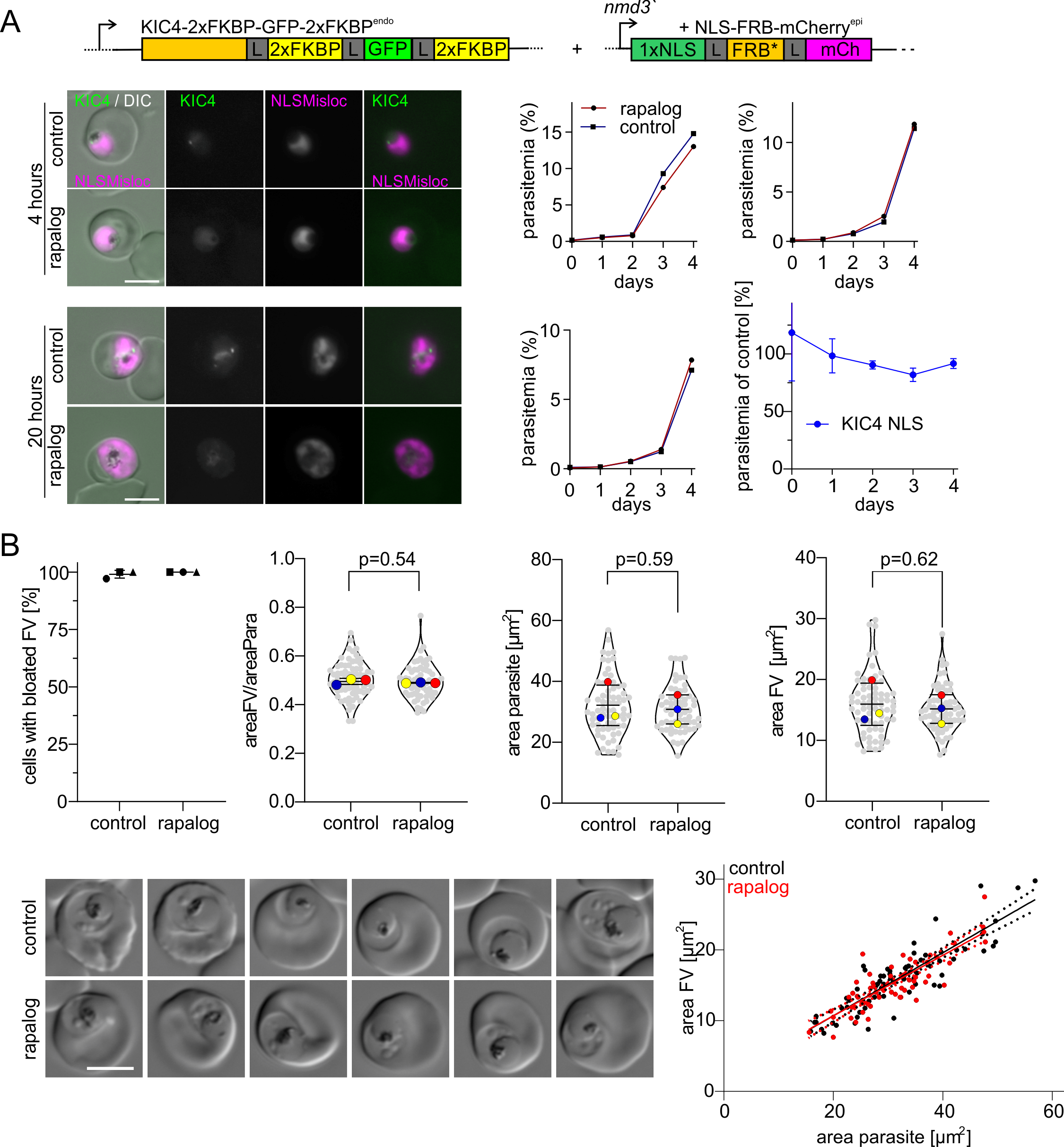

Supplement: S10 Fig — (A) Live-cell microscopy of knock sideways (+ rapalog) and control (without rapalog) KIC4-2xFKBP-GFP-2xFKBPendo+ 1xNLS mislocaliser parasites 4 and 20 hours after the induction of knock-sideways by addition of rapalog. Scale bar, 5 μm. Graphs show relative growth of asynchronous KIC4-2xFKBP-GFP-2xFKBPendo+1xNLSmislocaliser plus rapalog compared with control parasites over five days (3 independent experiments). Mean relative parasitemia ± SD of plus rapalog vs control of the 3 experiments is shown in the fourth graph. (B) Bloated food vacuole assay with KIC4-2xFKBP-GFP-2xFKBPendo+1xNLSmislocaliser parasites 8 hours after inactivation of KIC4 (+rapalog). Cells were categorized as with ‘bloated FV’ or ‘non-bloated FV’ and percentage of cells with bloated FV is displayed; n = 3 independent experiments with each n = 19–30 (mean 21.4) parasites analysed per condition. Representative DIC are displayed. Area of the FV, area of the parasite and area of FV divided by area of the corresponding parasites were determined. Mean of each independent experiment indicated by coloured symbols, individual data points by grey dots. Data presented according to SuperPlot guidelines [147]; Error bars represent mean ± SD. P-value determined by paired t-test. Area of FV of individual cells plotted versus the area of the corresponding parasite. Line represents linear regression with error indicated by dashed line. (TIF) [file ppat.1011814.s010.tif]
