# Supplementary material for: The Kelch13 compartment contains highly divergent vesicle trafficking proteins in malaria parasites
Source: PLoS Pathog. 2023 Dec 1;19(12):e1011814. doi: 10.1371/journal.ppat.1011814 (PMC10718435; doi:10.1371/journal.ppat.1011814)
Supplement: S1 Table — Ranked by enrichment: from high to low by the average log2 ratio normalized of four published Kelch13 BioID experiments [29]. (PDF) [file ppat.1011814.s011.pdf]

Table S1				
**Gene ID**	**PlasmoDB annotation**	**Protein Name or Symbol**	**average log2 Ratio normalized Kelch13 from Birnbaum et al. 2020**	**proteins analyzed in this study**
PF3D7_1343700	kelch protein K13	K13	6.29	
PF3D7_0606000	protein KIC1	KIC1	4.43	
PF3D7_1227700	protein KIC2	KIC2	4.03	
PF3D7_1246300	protein KIC4	KIC4	3.83	
PF3D7_0914400	protein KIC4	KIC3	3.64	
PF3D7_1438400	metacaspase-like protein	MCA2	3.63	X
PF3D7_1025000	Eps15-like protein	Eps15	3.50	
PF3D7_1138700	protein KIC5	KIC5	3.39	
PF3D7_1243400	conserved Plasmodium protein, unknown function		3.24	X
PF3D7_0813000	protein KIC7	KIC7	3.13	
PF3D7_1442400	protein KIC9	KIC9	3.03	
PF3D7_0609700	protein KIC6	KIC6	2.98	
PF3D7_0104300	ubiquitin carboxyl-terminal hydrolase 1, putative	UBP1	2.90	
PF3D7_0204300	*conserved Plasmodium protein, unknown function*		2.84	
PF3D7_1014900	protein KIC8	KIC8	2.78	
PF3D7_0915400	ATP-dependent 6-phosphofructokinase	PFK9	2.58	
PF3D7_1365800	*conserved Plasmodium protein, unknown function*		2.53	X
PF3D7_1122500	protein KIC10	KIC10	2.44	
PF3D7_1447800	calponin homology domain-containing protein, putative		2.19	X
PF3D7_1142100	conserved Plasmodium protein, unknown function	KIC11*	1.65	X
PF3D7_1345600	inner membrane complex protein		1.55	
PF3D7_0103100	*vacuolar protein sorting-associated protein 51, putative*	*VPS51*	1.55	X
PF3D7_0109000	*photosensitized INA-labeled protein PHIL1, putative*	*PhIL1*	1.49	
PF3D7_1117900	*conserved Plasmodium protein, unknown function*		1.46	
PF3D7_0408100	conserved Plasmodium protein, unknown function		1.45	
PF3D7_1247400	peptidyl-prolyl cis-trans isomerase FKBP35	FKBP35	1.45	
PF3D7_0721100	*conserved Plasmodium protein, unknown function*		1.42	
PF3D7_1016200	*Rab3 GTPase-activating protein non-catalytic subunit, putative*		1.41	
PF3D7_0708400	heat shock protein 90	HSP90	1.41	
PF3D7_1329100	myosin F	MyoF	1.32	X
PF3D7_1329500	conserved Plasmodium protein, unknown function	KIC12*	1.32	X
PF3D7_0717600	conserved Plasmodium protein, unknown function	IMC32	1.30	
PF3D7_0405700	lysine decarboxylase, putative	UIS14	1.23	X
PF3D7_0907200	*GTPase-activating protein, putative*		1.15	X
PF3D7_0822900	*conserved Plasmodium protein, unknown function*	*PIC2*	1.15	
PF3D7_1018200	*serine/threonine protein phosphatase 8, putative*	*PPP8*	1.13	
PF3D7_1141300	conserved Plasmodium protein, unknown function	APR1	1.11	

(dark purple)	KICs (Birnbaum et al. 2020)
(light purple)	new candidates
(pale purple)	new candidates not analyzed
(red)	BioID baits (Birnbaum et al. 2020)
(grey)	considered false positives / IMC proteins
*	renamed in current manuscript
italic	less stringent filtering group (significant with FDR<1% in 2 out of 4 reactions of any bait (Birnbaum et al. 2020))
