# Supplementary material for: The Kelch13 compartment contains highly divergent vesicle trafficking proteins in malaria parasites
Source: PLoS Pathog. 2023 Dec 1;19(12):e1011814. doi: 10.1371/journal.ppat.1011814 (PMC10718435; doi:10.1371/journal.ppat.1011814)
Supplement: S2 Table — (PDF) [file ppat.1011814.s012.pdf]

Table S2: Oligonucleotides	
name	sequence
MCA2(wt)-3xHA-T2A-Neo fw	ggtgacactatagaatactcaagctgcggccgcTAATGAGTACTCACCAGATGACATCAAATTTTTATCAC
MCA2(wt)-3xHA-T2A-Neo rv	CTGGAACATCGTATGGGTACATGGTGGTACCGGAAACACATTTAATATTCAAATCGATAATACC
MCA2(wt)-3xHA int check fw	GGAATATAATTTGAAATATGTAAAATTTAAACTGTCC
MCA2(wt)-3xHA int check rv	CATAAACACAAAAATTTAAAGATGGAGTGG
MCA2 (Y1334.)-GFP fwd	ggtgacactatagaatactcgcggccgctaaAATAATTTTAGCAAACCAAATTTTTTAGATAAATTTTTTTATG
MCA2 (Y1334.)-GFP rev	CAGCACCAGCAGCAGCACCTCTAGCacgcgtTTTTTTTAATTGTTCATATAACTTTTTATTTTTGGTC
Intcheck 5UTR MCA2 (Y1344.) fw	ATCCATAACAATAATAATAATATTGAGTGG
Intcheck 3UTR MCA2 (Y1344.) rv	AACTTTTTTGTTTTGGTATCTTTGTCAAGC
PfMyosinC_NotI_fwd	cagtgcggccgctaaatagtaataaaacatatgatgaaag
PfMyosinC_AvrII_rev	cagtcctaggtacaaaagacctgcgccagaaaac
Intcheck 5UTR MyoF wt fw	CAAATTATCAGGTCATAAAAAGGCAATAACATG
Intcheck 3UTR MyoF wt rv	ATATATATATATATGAACAAAATTTACAATATC
MyoF intcheck fw JSW175	GGCACACTTAAATCATATGAAC
MyoF intcheck rv JSW176	catgtgaatagaaaagtaaac
MyoF(wt)-3xHA-T2A-Neo fw	gctatttaggtgacactatagaatactcaagctgcggccgcTAATCTCAGAAGATAAAATTATTTCATCATC
MyoF(wt)-3xHA-T2A-Neo rv	CGTAATCTGGAACATCGTATGGGTACATGGTGGTACCTACAAAAGACCTGCGCCAGAAAACTATG
KIC11 TGD fw	gctatttaggtgacactatagaatactcgcggccgctaaAACGATAAAAAGAATAGCATTAATAAAAG
KIC11 TGD rv	cagcaccagcagcagcacctctagcacgcgtTATATGATTATTCTTTTTATTTATAG
KIC11 fw	gctatttaggtgacactatagaatactcgcggccgctaaGATGATGCTGACGAGGAGGAAGAAG
KIC11 rv	CAGCAGATCTTGATCTCAATCCTGAcctaggTTTTTTATCCTTTGTTTTAAGTTTAT
KIC11 int check 5 fw	ATGTAACATATGATAATAATAATG
KIC11 int check 3 rv	TATTAAAAAGAATTTCATTTGATG
KIC12fw	gctatttaggtgacactatagaatactcgcggccgctaaAGAGTGGTGGTAGTAACAACAATAG
KIC12 rv	CAGCAGATCTTGATCTCAATCCTGAcctaggATAATTTAACTCTGGGTGACCACTAAAC
KIC12 TGD fw	gctatttaggtgacactatagaatactcgcggccgctaaGGACAATTTATATTATAATAAATATAC
KIC12 TGD rv	cagcaccagcagcagcacctctagcacgcgtCATGTTTTTCTTGGCATTATAAAATG
KIC12 int check 5 fw	TTACAGCATATGTAAAGAATTCATC
KIC12 int check 3 rv	TTTCCAATCAACCTATATATGTGTG
KIC12 TGD int check 5 fw	AATGGGCATATTCTATTTTCGTGTG
KIC12 TGD int check 3 rv	AACATCAAGCAAAAAAATAACATCC
VPS51 fw	tatttaggtgacactatagaatactcgcggccgctaaAGTGATATGTATGAAAAAATTGAAG
VPS51 rv	AGCAGCAGATCTTGATCTCAATCCTGAcctaggCTCTTTAAACATTTTCAATATAAATAATTTATTTTC
VPS51 int check 5 fw	TGAAGGACAGGAAAGAGAATATG
VPS 51 int check 3 rv	ATGAATCTATCCTTTCTTTACTC
VPS51 TGD fw	gctatttaggtgacactatagaatactcgcggccgctaaAATAAAAATAATAGAAGAAAAAATG
VPS51 TGD rv	ccagcaccagcagcagcacctctagcacgcgtCTTTTTCGATTTATTTATTAAATC
VPS51 TGD int check 5 fw NEW	cgcctgggagtcattcctaaatttg
VPS 51 TGD int check 3 rv NEW	GATGTACACATTTGTTATTATCCC
PF3D7_1243400 rv	AGCAGCAGCAGATCTTGATCTCAATCCTGAcctaggAGAAGCTACCTTTTGTATTTCC
PF3D7_1243400fw	aagctatttaggtgacactatagaatactcgcggccgctaaGAGAACGAACAAAAGATATCCTTC
PF3D7_1447800 fw	gctatttaggtgacactatagaatactcgcggccgctaaAATAATATACATAATAATAATAAAC
PF3D7_1447800 rv	CAGCAGATCTTGATCTCAATCCTGAcctaggATCTTGACCTGTGATCATATTTTC
PF3D7_1447800 int check 5 fw	ATGATAAAAATAATAATGCACATAG
PF3D7_1447800check 3 rv	AGCAATATATCTAACCAATGGACAC
PF3D7_1365800 fw	gctatttaggtgacactatagaatactcgcggccgctaatattttagAAATTCAAGTATCAGG
PF3D7_1365800 rv	CAGCAGATCTTGATCTCAATCCTGAcctaggTTGGGGGAATATAGGTGTAATATTAG
PF3D7_1365800 int check 5 fw	tatatgtgcacatatttatatttac
PF3D7_1365800 int check rv NEW JSW	gtgatgtccatataaataatgttgac
PfUIS14fw	gctatttaggtgacactatagaatactcgcggccgctaaATTGTAATTATGTTAAAAAATGTG
PfUIS14 rv	CAGCAGATCTTGATCTCAATCCTGAcctaggCTTGTGTTTGGTTTCACAGTCATC
PfUIS14 TGD fw	gctatttaggtgacactatagaatactcgcggccgctaaGAACAATATAGATCAGAATAAAATC
PfUIS14 TGD rv	cagcaccagcagcagcacctctagcacgcgtATTAATTATCTCATGTAAATTTTCTG
PfUIS14 int check 5 fw	CTAGCTAGTAATAACAATTATTGTG
PfUIS14 int check 3 rv	ATATAATATATATAATTCCATGCTG
PfUIS14 TGD int check 5 fw	TTTCAAATCGATGAGGATTCTTTAC
PfUIS14 TGD int check 3 rv	TAGAAACTTCTTTATATTTTTTATC
PF3D7_0907200 fw	gctatttaggtgacactatagaatactcgcggccgctaaTGCATGAACTTAATATGTGTGATAATG
PF3D7_0907200 rv	CAGCAGATCTTGATCTCAATCCTGAcctaggAAATGTGTGAGCATCGTCTACCAG
PF3D7_0907200 int check 5 fw	TAATCAAAATATAAAAAATCATCAC
PF3D7_0907200 int check 3 rv	TCAAGAAAAAAAATTTAATTATACG
PF3D7_1243400 TGD fw	cactatagaatactcgcggccgctaaTTTCCCAGTTTTAATAA
PF3D7_1243400 TGD rv	ctgatattaacttctgctcgtttaaacCTTCTCTTGGTTATCTTGTA
ama1 fw NotI	GGTGACACTATAGAATACTCgcggccgcGAGGTGTGTTGGGAAACAGAAG
ama1 rv KpnI	TCCTggtaccTTTGTACAATTTATAACAAGTAC
IMC1c fw KpnI	CTCGggtaccATGGCAGATTCAATCAAAAGTTCAAACAG
ARO fw KpnI	CTCGggtaccATGGGAAATAATTGCTGTGCAGGAAG
AMA1 fw KpnI	CTCGggtaccATGAGAAAATTATACTGCGTATTATTATTG
mCherry rv XmaI	GAACATTAAGCTGCCATATCCCTCGACCCGGGTTACTTGTACAGCTCGTCCATGCCGCC
