# Supplementary material for: The Kelch13 compartment contains highly divergent vesicle trafficking proteins in malaria parasites
Source: PLoS Pathog. 2023 Dec 1;19(12):e1011814. doi: 10.1371/journal.ppat.1011814 (PMC10718435; doi:10.1371/journal.ppat.1011814)
Supplement: Attachment — Submitted filename: 132768_1_rebuttal_2748733_ryf6yr.pdf [file ppat.1011814.s014.pdf]

Full Revision

Manuscript number: RC-2022-01801

Corresponding author(s): Tobias Spielmann

1. General Statements [optional]:

We are very grateful for the constructive and very thorough assessment of all three reviewers and carefully edited the manuscript according to their feedback and suggestions. This included the following additional experimental data to address the comments:

- 3D7 +/- rapalog bloated assay control experiment (Figure S3I)
- MCA2-TGD, MCA2Y1344, 3D7 bloated FV assays (Figure S5C-E)
- KIC4-TGD, KIC5-TGD bloated FV assays (Figure S9)
- MCA2-GFP/K13mCherry co-localisation in schizont stage parasites (Figure 4C)
- MyoF-GFP/K13 co-localisation in ring stage and schizont parasites (Figure 1C, S2A)
- extended MyoF bloated FV assay analysis (Figure 1J-K, S2J)
- MyoF-3xHA growth and vesicle accumulation assays (Figure S2F-G)
- KIC12 expression/location in free merozoites (Figure S4A-B)
- KIC12/K13 overlap quantification (Figure S4A)
- KIC11/K13 overlap quantification (Figure 2C)
- RSA with parasites under Cytochalasin D (Figure 1M)
- KIC11 invasion/egress assay (Figure 2H, S3G)
- KIC11 mislocalization efficacy 80h post induction (Figure S3E)
- KIC11 ARO/AMA1/IMC1c co-localization (Figure S3A)
- effect of KIC11 inactivation on ARO/AMA1/IMC1c (Figure S3A)
- PF3D7_1365800 mislocalisation with LYN mislocalizer (Figure S7C-D)
- Non successful attempt to generate N-terminal 2xFKBP-GFP-2xFKBP^{endo}-PF3D7_1243400

In addition we also provided a new figure part showing the correlation of level of inactivation of MyoF with the degree of endocytosis phenotypes (Figure 1L) to better illustrate the consistency of the functional data with that protein.

Besides including additional data, we carefully re-structured the manuscript following the suggestion of the reviewers to increase readability.

Finally, in an attempt to keep the manuscript up to date, we also included citations to the following relevant publications that appeared when our work was under review and in revision:

- Wan et al. 2023: The Toxoplasma micropore mediates endocytosis for selective nutrient salvage from host cell compartments Nat Commun. DOI:10.1038/s41467-023-36571-4
- Sabitzki et al. 2023: Identification of a Rabenosyn-5 like protein and Rab5b in host cell cytosol uptake reveals conservation of endosomal transport in malaria parasites, bioRxiv
- Tutor et al. 2023: The Plasmodium falciparum artemisinin resistance-associated protein Kelch 13 is required for formation of normal cytostomes. bioRxiv
- Liffner et al. 2023: Atlas of Plasmodium falciparum intraerythrocytic development using expansion microscopy. bioRxiv
- Devarakonda et al. 2023 Trafficking of nuclear-encoded apicoplast proteins depends on F-actin and Myosin F in Toxoplasma gondii. bioRxiv

- Morano et al. 2023 A PPP-type pseudophosphatase is required for the maintenance of basal complex integrity in Plasmodium falciparum. Nat Commun. DOI:10.1038/s41467-023-39435-z

2. Point-by-point description of the revisions

Please find our point-to-point response to the reviewer's comments below (in blue font), where we marked all changes implemented in the manuscript in *blue+italics font*.

Reviewer #1 (Evidence, reproducibility and clarity (Required)):

With the emergence and spread of resistance to Artemisinin (ART), a key component of current frontline malaria combination therapies, there is a growing effort to understand the mechanisms that lead to ART resistance. Previous work has shown that ART resistant parasites harbour mutations in the Kelch13 protein, which in turn leads to reduced endocytosis of host haemoglobin. The digestion of haemoglobin is thought to be critical for the activation of the artemisinin endoperoxide bridge, leading to the production of free radicals and parasite death. However, the mechanisms by which the parasites endocytose host cell haemoglobin remain poorly understood.
Previous work by the authors identified several proteins in the proximity of K13 using proximity-based labelling (BioID) (Birnbaum et al. 2020). The authors then went on to characterise several of these proteins, showing that when proteins including EPS15, AP2mu, UBP1 and KIC7 are disrupted, this leads to ART resistance and defects in endocytosis leading to the hypothesis that these two processes are inextricably linked.
In this manuscript, Schmidt et al. set themselves the task of characterising more K13 component candidates identified in their previous work (Birnbaum et al. 2020) that were not previously validated or characterised. They chose 10 candidates and investigated their localisations, and colocalisation with K13, and their involvement in endocytosis and in vitro ART resistance, 2 processes mediated by K13 and some members of the K13 compartments

The authors show that of their 10 candidates, only 4 can be co-localised with K13. Then, using a combination of targeted gene disruption (TGD) as well as knock sideways (KS), they characterised these 4 proteins found in the K13 compartment. They show that MyoF and KIC12 are involved in endocytosis and are important for parasite growth, however their disruption does not lead to a change in ART sensitivity. The authors also confirm the findings of their previous publication (Birnbaum et al. 2020), using a slightly different TGD (**note from the authors:** we apologise if this has not properly transpired from the manuscript but the difference between the TGDs is substantial and relevant: one has less than 3% of the protein left and hence can be considered to fully inactivate MCA2 and has a growth defect whereas the other contains about two thirds of the protein (1344 amino acids/~66% are left), has no growth defect, although it lacks the MCA2 domain (hence that domain can not be critical for the growth defect)), that MCA2 is involved in ART resistance, however they did not check whether its disruption impacts haemoglobin uptake. They also show that KIC11 is not involved in mediating haemoglobin uptake or ART resistance. To finish, the authors used AlphaFold to identify new domains in the proteins of the K13 compartment. This led them to the conclusion that vesicle trafficking domains are enriched in proteins of the K13 compartment involved in endocytosis and in vitro ART resistance.

The majority of the experiments conducted by the authors are performed to a good standard in biological and technical replicates, with the correct controls. Their findings provide confirmation that their 4 candidate genes seem to be important for parasite growth, and show that some of their candidates are involved in endocytosis. While the KD and KS approaches employed by the authors to study their candidate genes each have their own advantages and can be excellent tools for studying a large sets or genes, this manuscript highlights the many limitations of these approaches. For example, the large tag used for the KS approach can mislocalise proteins or disrupt their function (as is the case for MyoF), resulting in spurious results, or indeed the inability to generate the tagged line (as is the case for MCA2). The KS approach also makes the results of a protein with a dual localisation, like KIC12, extremely difficult to interpret.
We thank the reviewer for this thorough and insightful review.

The limitations mentioned above were addressed in the response to the main points and a general detailed response in regards to the systems used for this research are added at the end of this rebuttal. Briefly summarised here: while we agree that there are limitations of the system used, we are convinced that
- the advantages of using a large tag in most cases outweighs the drawbacks as it permits to track the inactivation of the target, if need be on the individual cell level
- while not optimal for MyoF, the partial inactivation actually helps in its functional study as detailed in major point 23&28 or reviewer#3 major point 11: it shows a consistent correlation of the phenotype with different causes and degrees of inactivation (this is now better illustrated in Figure 1L1M). Further, regarding the concern of the large tag: the effect of the tag based on localisation was overestimated in the review by what seems to have been a mix up comparing numbers from MyoF with a number from MCA2 (there is a difference, but it is only small) (see reviewer#1 major point #23).
- KS is the optimal method for most of the assays in this work (e.g. bloated food vacuole assays and RSAs); these assays would be impossible or difficult to use with other inactivation systems currently used in *P. falciparum* research (see details in the response to the specific points and after the rebuttal)

In regards to the difficulty to interpret KIC12 data: this is only true for measuring absolute essentiality, everything else we believe we actually have the optimal method. If not KS, which method targets a specific pool of a protein with a dual localisastion? Again, our assays targeting the K13 pool and revealing the specific function would have been difficult or impossible with any other system.

Ultimately the question is whether any other system would have resulted in a different conclusion on the function of the proteins studied. At present we are confident this would not be the case and other systems probably would not have delivered the specific functional data shown in this work. Clearly, more in depth work will provide more nuanced and detailed insights into the proteins analysed in this work and this likely will also include the use of other systems for specific aspects they are most suitable for. However, this (e.g. different complementations in a diCre cKO) is complex and therefore beyond what fits into this work which had the goal to assess which proteins are true positives for the K13 compartment and to place them into functional groups in regards to endocytosis.

Moreover, the manuscript is disjointed at times, with the authors choosing to conduct certain

experiments for only a subset of genes, but not for others. For example, considering that the aim of this paper was to identify more proteins involved in ART resistance and endocytosis, it is confusing why the authors do not perform the endocytosis assays for all their selected proteins, and why they do not do this for the proteins they identify in their domain search. There is significant room for improvement for this manuscript, and a generally interesting question.
The reviewer remarks that not every experiment was done for every target. Based on the rebuttal we tried to amend this but also note that there was some sentiment by the reviewers to better stick to the point and not make the manuscript more disjointed. We attempted to balance that as much as possible and hope we were able to honour both aspects (amendments were done as detailed in the point by point response below).

In regards to endocytosis and choice of targets: We did do endocytosis assays for all proteins that showed a growth phenotype upon inactivation in this work. We therefore assume the reviewer here refers to major point #40 asking for endocytosis assays with KIC4 and KIC5 (which were not studied in this manuscript) as well as MCA2 (point 17). We fully agree with the reviewer that this would fill a gap in the work on K13 compartment proteins but such assays are difficult with TGDs (there are issues with non-comparable samples and compensatory effects) and proteins that are not essential (and hence likely have a smaller impact on endocytosis when truncated). We nevertheless now carried them out, but due to the limitations to do this with these lines would be hesitant to draw definite conclusions (see major point 17 and 40 for details and outcomes).

But in it's current format, other than confirming that MCA2 is involved in ART resistance (which was already known from the Birnbaum paper), the authors do not further expand our understanding of the link between ART resistance and endocytosis in this manuscript.
We would like to point out that the importance of the K13 compartment and endocytosis goes beyond ART resistance (see e.g. also newly published papers on the K13 compartment in Toxoplasma, (Wan *et al.*, 2023; Koreny *et al.*, 2023)). Endocytosis is an essential and prominent process in blood stages. However, in contrast to processes such as invasion, our understanding about endocytosis is only rudimentary. Hence, this manuscript provides important insights on an emerging topic that in our opinion deserves more attention:

- it identifies novel proteins at the K13 compartment and provides 2 new proteins in endocytosis (MyoF and KIC12); getting an as complete as possible list of proteins involved in the process will be critical to study and understand it

- it leads to the realisation that not all growth-relevant proteins detected at the K13 compartment are needed for endocytosis

- it provides domains and stage specificity of function for several K13 compartment proteins, overall bolstering the model of endocytosis in ART resistance and providing a framework critical to direct future studies on endocytosis and their detailed mechanistic function at the cytostome

- the identified vesicle trafficking domains (for instance now also found in UBP1) are expected to strengthen the support for the role of endocytosis of the K13 compartment; this and also the above points are important as (based on the current literature) there still seems to be

prominent sentiment in the field that (in part due to the involvement of UBP1 and K13) the cause of ART resistance is due to various unclearly defined stress response pathways

- with MyoF it also shows the first protein in connection with the K13 compartment that acts downstream of the generation of hemoglobin-filled containers in the parasite and provides the first protein that explains the suspected involvement of actin in endocytosis (so far this was only based on CytD studies)

Overall we therefore believe this manuscript contains critical information and a framework for future studies on endocytosis and the K13 compartment. We hope the relevance of endocytosis as one of the most prominent and essential processes in the parasites and the connection to various aspects linked with many commercial drugs (in addition to the role of endocytosis in ART resistance), is adequately explained in the introduction. We also would like to mention that the main focus of the work is reflected in the title of the manuscript which does not mention ART susceptibility.

Major Comments

1) line 31: please change defined to characterised - defined suggests that novel proteins were identified in this study, which is not the case.
We apologise, but we do not fully understand this comment. We did identify novel proteins not before known to be at the K13 compartment (MCA2 (admittedly this one was likely but had not previously been verified), MyoF, KIC11 and KIC12). In our view "further defining the composition of the K13 compartment" therefore is an accurate statement. Additionally, the identification of previously not-discovered domains, the stage-specificity and function of these proteins helped to further define the K13 compartment.
If the reviewer is referring to the fact that the proteins analysed in this study were taken from a previously generated list of hits, we would like to stress that the presence in such a list (obtained from a BioID, but also if from an IP etc) can not be equalled for them to be true positives, they are merely candidates that still need to be experimentally validated. This is what we did in this work to find out which further proteins from the list can be classified as K13 compartment proteins (for hits with lower FDRs this is even more relevant as illustrated by the fact that 6 of the here analysed hits were not at the K13 compartment). In an attempt to address this comment in the manuscript, we changed the wording of this sentence to (line 31)*: "Here we further defined the composition of the K13 compartment by analysing more hits from a previous BioID, showing that MyoF and MCA2 as well as Kelch13 interaction candidate (KIC) 11 and 12 are found at this site."*

2) line 37: please change 'second' to "another". As explained further below, the authors identified 3 classes of proteins (confer ART resistance + involved in HCCU, involved in HCCU only, or involved in neither).
We realized that the groups description wasn't clear in the abstract. Please see response to major comment #41 for a detailed answer to this (endocytosis is an overarching criterion, ART resistance is a subgroup and applies only to those proteins with a function in endocytosis in ring stages). To clarify this (see also major point #8) we added an explanation on the influence of stage-specificity of endocytosis on ART susceptibility to the introduction (line 76): *"In contrast to K13 which is only needed for endocytosis in ring stages (the stage relevant for in vitro ART resistance), some of these proteins (AP2μ and UBP1) are also needed for*

endocytosis in later stage parasites (Birnbaum *et al.*, 2020). *At least in the case of UBP1, this is associated with a higher fitness cost but lower resistance compared to K13 mutations* (Behrens *et al.*, 2021; Behrens *et al.*, 2023). *Hence, the stage-specificity of endocytosis functions is relevant for in vitro ART resistance: proteins influencing endocytosis in trophozoites are expected to have a high fitness cost whereas proteins not needed for endocytosis in rings would not be expected to influence resistance."* The abstract was changed in response to this and other comments and hope it is now clearer in regards to the groups.

3) Line 40: You define KIC11 as essential but according to your data some parasites are still alive and replicating 2 cycles after induction of the knock sideways. Please consider changing "essential" to "important for asexual parasite growth".
We fully agree with the reviewer, we reworded the sentence as suggested.

4) Line 40: please change 'second group' to 'this group'
We reworded this part of the abstract and it know reads: (line 38): *"While this strengthened the link of the K13 compartment to endocytosis, many proteins of this group showed unusual domain combinations and large parasite-specific regions, indicating a high level of taxon-specific adaptation of this process."*

5) line 41: state here that despite it being essential, it is unknown what it is involved in.
With the newly added data we show that this protein either has a function in invasion or very early ring development although we did not see any evidence for the latter. We therefore changed the sentence to (line 43): *"We here identified the first protein of this group that is important for asexual blood stage development and showed that it likely is involved in invasion.."*

6) Line 50: the authors should state here that there is actually a reversal in this trend over the last few years.
Done as suggested.

7) Line 54: please separate out the references for each of the two statements made in this line (a: that ART resistance is widespread in SEA, and b: that ART resistance is now in Africa) Reference 14 also seems to reference ART resistance in Amazonia - which is not covered by the statement made by the authors (in which case the authors should state ART is now present in Africa and South America). The authors should also reference PMID: 34279219 for their statement that ART resistance is now found in Africa (albeit a different mutation to the one found in SEA).
Done as suggested.

8) Line 65: it is also worth mentioning here that there are other mutations in proteins other than K13, such as AP2mu and UBP1 (PMID: 24994911;24270944) that can lead to ART resistance.
As suggested by the reviewer, we included a sentence about non-K13 mutations linked with reduced ART susceptibility in the introduction (line 74): *"Beside K13 mutations in other genes, such as Coronin (Demas et al., 2018) UBP1 (Borrmann et al., 2013; Henrici et al., 2020b; Birnbaum et al., 2020; Simwela et al., 2020) or AP2µ (Henriques et al., 2014; Henrici et al., 2020b) have also been linked with reduced ART susceptibility."*
We here also added data on fitness cost that is related to this and is also relevant for the issue of proteins with a stage-specific function in endocytosis, making a transition for this statement

which might help clarifying the grouping of K13 compartment proteins (see also major point #2).

9) Line 80, 86: ref 43 is misused. Reference 43 refers to Maurer's clefts trafficking which takes place in the erythrocyte cytosol and is not involved in haemoglobin uptake as far as I know. Please replace ref 43 with one showing the role of actin in haemoglobin uptake.
We thank the reviewer for pointing this out, Ref 43 was removed from the manuscript.

10) Line 98: the authors state here that they 'identified' further candidates from the K13 proxiome. This suggests that they identified new proteins in this paper, when in fact the list was already generated in ref 26. All they did was characterise proteins from that list that were not previously characterised. The authors should therefore remove identified from this statement.
We agree with the reviewer that we did not identify further candidates, we identified new K13 compartment proteins from the list of potential K13 compartment proteins. We therefore changed “identified further candidates” into “*identified further K13 compartment proteins*” (line 116). Please see also response to major comment #1.

11) Line 107-108: it is not clear from this sentence why these proteins were left out of the initial analysis in Ref 26. A sentence here explaining this would be valuable for the reader.
This is a good point. One reason why we did not analyse more in our previous publication was that we had to stop somewhere and adding more would have been very difficult to fit into what was already a packed paper. However, as shown in this work, the list does contain further interesting candidates (e.g. K13 compartment proteins that are involved in endocytosis).
We altered the relevant part of the introduction to highlight that we previously analysed the top hits, clarifying that the 'remaining' hits analysed in this work were further down in the list. This now reads: (line 113)“*We reasoned that due to the high number of proteins that turned out to belong to the K13 compartment when validating the top hits of the K13 BioID* (Birnbaum *et al.*, 2020)*, the remaining hits of these experiments might contain further proteins belonging to the K13 compartment.”* We hope this clarifies that we simply moved further down in the candidate list.

12) Line 117-123: The authors say that PF3D7_0204300, PF3D7_1117900 and PF3D7_1016200 were not studied because they were not in the top 10 hits. However, the current organisation of Supplementary Table 1 shows all 3 proteins among the top 10 hits (MyoF, KIC12, UIS14 and 0907200 being after them). I think the authors should reorganise their table. It is also unclear according to what the proteins in the table are ranked. Could the authors indicate the metric used for the ranking?
We thank the reviewer for alerting us to this. The issue here is that the 3 non-analysed proteins belong to a 'lower stringency' group comprising hits significant with FDR<1% in only 2 out of 4 reactions of any bait (Birnbaum et al. 2020; in the supplementary data table S1 of this paper, these proteins appear, but are in light grey font to indicate they are not part of our hit list according to the stringent selection criteria (FDR<1% in 3 out of 4 reactions of any bait)). To account for this, we now marked all proteins that were from this 'lower stringency' group in italic font in Table S1, similar to Birnbaum et al. 2020. There are 10 such proteins in the list (as now evident in the revised table S1). The problem with these 'lower' hits is that they do not consistently have values in all experiments (e.g. 2 of the non-selected were no enriched/absent in the Eps15 BioID) and some also have an enrichment in one or more

clathrin BioIDs (which we considered as a control negative list based on the Birnbaum et al., 2020 paper). This would be very complex to add to this table which is ranked based on the average K13 enrichment with the low stringency hits included. What we did for this work is to take three out of the 10 lower stringency hits to 'assess' this group. None of the selected ones (PF3D7_1365800, PF3D7_0103100 and PF3D7_0907200) were actually at the K13 compartment, hence we do not consider this group a good source to find more K13 compartment proteins and did not include further ones for validation. In order to not further complicate the manuscript, and particularly because these hits did not turn out to be K13 compartment proteins, we hope that simply highlighting the lower stringency hits in the table will suffice to explain this issue.
The information about ranking is now also included as "Table legend" in the revised manuscript and the Table heading has been changed to: *"List of putative K13 compartment proteins, proteins selected for further characterization in this manuscript are highlighted."*

13) Line 129-141: Can the authors be clearer with their explanations of the identification of mutation Y1344Stop? One dataset (ref 61) shows that 52% of African parasites have a mutation in MCA2 in position 1344 leading to a STOP codon. But another dataset (ref 62) shows that the next base is also mutated, reverting the stop codon. That should have been seen in the first dataset as well. Could the authors please clarify.
This mutation was first spotted in the MalariaGEN database (https://www.malariagen.net) (MalariaGEN *et al.*, 2021), which allows online accessing of the data by using the "variant catalogue" tool, which is in a table format of frequency rather than in a sequence context. Hence, only after further research later on it became evident to us, that this mutation does not occur alone when looking at individual MCA2 sequences from patient samples in (Wichers *et al.*, 2021b). We hope this is accurately reflected in our results section.

14) Line 147: the authors say that MCA2 is expressed throughout the intraerythrocytic cycle as shown by live cell imaging. In Birnbaum et al 2020 fig 4I, the authors show that MCA2 is mainly expressed between 4 and 16hpi. But in Figure 1B of this manuscript there is a clear multiplication of MCA2 signal between trophozoite and schizont. How do the authors explain this discrepancy? Could expression of the truncated MCA2 be different than the full length? This cannot be assessed as expression and localisation of the full-length HA tag MCA2 is not shown in Schizonts.
The key difference lies in transcription vs protein expression (usually protein levels peak after mRNA levels peak and - depending on turnover - protein levels can stay high even after mRNA levels have declined). Figure 4 of the Birnbaum et al paper presents transcriptomic data, but with a peak in trophozoites (The axis label in Fig. 4I of that publication is a bit confusing, as hour 0 is at the top, 48 h at the bottom; it is clearer in Fig. S13 of that paper) which would fit very well with the multiplication of the signal between trophozoites and schizonts mentioned by the reviewer. So, overall, the temporal peaks of transcripts and protein of that protein fit well.
For the signal in rings: Likely the protein has a turnover rate that is sufficiently low for some protein to be taken into the new cycle after re-invasion. Also different transcriptomic datasets e.g. (Otto *et al.*, 2010; Wichers *et al.*, 2019; Subudhi *et al.*, 2020) available on plasmoDB show some mRNA present across the complete asexual development cycle, with each dataset showing maximum peak at a slightly different stage.
Even when located in foci and hence aiding detection of small amounts of protein (as is the case for MCA2-Y1344-GFP), the MCA2 signal in rings is not strong. For MCA2-TGD, the GFP

signal is dispersed and therefore likely below our detection limit, while the same amount of protein concentrated at the K13 compartment is visible as foci in the MCA2-Y1344 cell line. Please note that MCA2-TGD has only 2.8% of the protein left whereas MCA2-Y1344 has 66.5% left and based on our manuscript is almost fully functional, hence fitting the different locations between the two versions.
Overall we believe this shows that there are actually no significant discrepancies of the expression of the different MCA2 versions.

15) Line 158: would it not have been more useful for the authors to have episomally expressed MCA2-3xHA in their MCA2Y1344STOP-GFPENDO line to make sure that the truncated protein is indeed going to the correct compartment? The experiments done by the authors suggests that the MCA2Y1344STOP goes to the right location but does not really confirm it.
We appreciate the reviewers caution here. However, considering that MCA2Y1344STOP-GFP^{endo} co-locates with mCherryK13 and endogenously HA-tagged full length MCA2 does the same to a similar extent, there is in our opinion little doubt that MCA2 is found at the K13 compartment and that this is similar with both constructs. If there are minor differences, these might as well occur if MCA2 is episomally (as suggested in the comment) instead of endogenously expressed. Given the limited insight, we therefore decided against the episomal overexpression (which due to its size of > 6000bp may also be somewhat less straight forward than it may sound).

16) Line 191: it is stated that MCA2 confers resistance independently of the MCA domain, however in both the MCA2-TGD and MCA2Y1344STOP-GFPENDO parasites, the MCA domain is deleted, and for both parasites, there is resistance (albeit to a lower level in the MCA2Y1344STOP-GFPENDO line). Therefore, how can the authors state that the ART resistance is independent of the MCA domain? This statement should be that resistance is dependent on the loss of the MCA domain.
We agree that this can't be categorically excluded. However, a ~5 fold difference in ART sensitivity was observed between the parasites with MCA2 truncated at amino acid 57 compared to those with MCA at amino acid 1344 even though both do not contain the MCA2 domain. Hence, at least this difference is not dependent on the MCA2 domain. The larger construct missing the MCA domain shows only a very moderate reduction in RSA survival, again suggesting the MCA domain is not the main factor. We amended our statement in an attempt to more accurately reflect the data (line 487): *"This considerable reduction in ART susceptibility in the parasites with the truncation at MCA2 position 57 compared to the parasites still expressing 1344 amino acids of MCA2, despite both versions of the protein lacking the MCA domain, indicates that the influence on ART resistance is not, or only partially due to the MCA domain."* We would be hesitant to state the reviewer's conclusion that "resistance is dependent on the loss of the MCA domain", as the larger construct missing the MCA2 domain has a milder RSA effect compared to MCA2-TGD, which suggests the reduction in ART susceptibility is independent of the MCA domain. These considerations also agree with the fact that the parasites with the longer MCA2 version (in contrast to the MCA2-TGD) do not have any detectable growth defect which indicates that the protein can fulfil its function without the MCA2 domain.

17) Line 192: Why did the authors not check if MCA2 is involved in endocytosis? They state later on in the manuscript that they did not do endocytosis assays with TGD lines, however if the authors include the correct controls, this could be easily done. It would also be really

interesting to see whether endocytosis gets progressively worse going from WT to MCA2Y1344STOP to MAC2TGD. This experiment (as well as doing endocytosis assays for KIC4 and KIC5 TGD lines) would drastically increase the impact of this study. These experiments would not take more than 3 weeks to perform, and would not require the generation of new lines.

So far were very hesitant to do bloated FV assays with TGDs (even though TGDs were available for the genes encoding MCA2 and KIC4 and KIC5). The reason for this was:

1. the fact that these proteins could be disrupted indicated either redundancy or only a partial effect on endocytosis which might lead to only small effects that likely are difficult to pick up in an assay scoring for the rather absolute phenotype of bloated vs non-bloated. Using the refined assay measuring FV size could partly amend this but we note that also FV without hemoglobin have a certain size, reducing the relative effect if there are smaller differences.

2. a TGD line does not permit tightly controlled inactivation of the target which makes comparing the outcome of bloated food vacuole assays difficult if there are smaller growth and stage differences to the 3D7 control.

3. in contrast to conditional inactivation parasites, the TGD lines had ample times to adapt to loss of the target protein (compensatory mechanisms are well known for endocytosis, for instance in clathrin mediated endocytosis loss of individual components can be compensated (Chen and Schmid, 2020)).

We nevertheless see the reviewer's point that this should at least be attempted and now conducted these assays (see also major point 40). For MCA2 (as requested in this point), the data is shown in Figure S5C-E. This assay showed that in MCA2-TGD, MCA2Y1344STOP-GFP^{endo} (similar to the 3D7 control) >95% of parasites developed bloated food vacuoles. Additionally, we also measured the parasite and food vacuole size of individual cells in an attempt to solve some of the problems with TGDs with such assays. In order to specifically solve problem 2 mentioned above, we analysed the food vacuoles of similarly sized parasites, however, they were non-distinguishable between the three lines. Of note, in agreement with the reduced parasite proliferation rate (Birnbaum *et al.*, 2020) a general effect on parasite and food vacuole size was observed for MCA2-TGD parasites, indicating reduced development speed in these parasites. Hence, it is possible that a potential endocytosis reduction was accompanied by a slowed growth, and the comparison of similarly sized parasites may have obscured the effect. It is therefore not sure if there indeed is no endocytosis phenotype, although we can exclude a strong effect in trophozoites.

Based on the RSA results at least rings can be expected to have a reduced endocytosis in the MCA2-TGD. Apart from options 1-3 mentioned above, it is therefore possible there is an effect restricted to rings, although in that case the reduced growth in trophozoites would be due to other functions of MCA2. Overall, we can conclude that the MCA2-TGD parasites do not have a strongly reduced endocytosis, but given the fact that the parasites are viable, this is not surprising. Whether the MCA2-TGD has no effect at all on endocytosis we would be very hesitant to postulate based on these results.

18) The authors should consider re-organising the MCA2 section, first showing that the 3xHA tagged line colocalises with K13, then performing the new truncation.

We attempted to re-organise as suggested but because we now included additional fluorescence microscopy images of schizont and merozoites (in response to reviewer 2 major comment 3) the main figure would become even larger. To prevent this, we kept the 3xHA data in the supplement.

19) Line 197: Once again ref 43 is not correct to illustrate that actin/myosin is involved in endocytosis

We thank the reviewer for pointing this out – we removed Ref 43.

20) Line 202: the authors state that MyoF localises near the food vacuole from ring stage/trophs onwards. However, how can this statement be made in schizonts based on these images (Fig. 2A), where it doesn't look like MyoF is anywhere near the FV? This statement can only be made for schizonts if co-localised with a FV marker (which is done in Fig. 2B), however, based on the number of MyoF foci, it appears that this was not done for schizonts. Please either remove the statement that MyoF is near the food vacuole from trophs onwards (because it is only seen near the FV up until trophs) or show the data in Fig. 2B of schizonts to substantiate these claims.

This is a valid point. We originally did not focus on schizonts because most markers end up in some focal area in the forming merozoite but other proteins (such as e.g. K13) also have one or more additional foci at the FV, making interpretation unclear, particularly if the schizont is still organizing to become fully segmented. This is why we generally focused the K13 co-localisations on the trophozoite stage to obtain the clearest information on endocytosis. However, given the fact that this manuscript gives the first localization of MyoF in *P. falciparum* parasites, we now provide a comprehensive time course (Figure 1C, S1A) including schizonts, which show quite a complex pattern: while the MyoF-GFP localization in trophozoites appeared as multiple foci close to K13 and also the FV, the MyoF-GFP pattern changes in late schizonts (fully segmented) and merozoites, appearing as elongated foci no longer close to K13 or the FV. Of note, this pattern has been previously reported for MyoE in *P. berghei* (Wall *et al.*, 2019).

We therefore revised the statement about MyoF localization in schizont to better reflect the observed localization: (line 175): *"In late schizonts and merozoite the MyoF-GFP signal was not associated with K13, but showed elongated GFP foci (Figure 1C, S2A) reminiscent of the MyoE signal previously reported in P. berghei schizonts* (Wall *et al.*, 2019)*."*

21) Line 204-206: what does this statement bring to the paper? Is it to show that it is the real localisation of MyoF because 2 tag cell line show the same localisation? I don't think this is needed, especially as later in the manuscript an HA-tag MyoF line is used and show similar localisation.

We see the reviewers point, but prefer to keep this data included in the supplement, particularly because potential differences in the location of tagged MyoF were a major concern.

Related to the tag issue: in order to get a better understanding of the effect of C-terminally tagging with different sized tags we now performed a more detailed analysis of the MyoF-3xHA cell line (Figure S2F-G), showing that this cell line shows a growth rate similar to the 3D7 wild type parasites, and has less vesicles than the 2x-FKBP-GFP-2xFKBP cell line, but still slightly, but significantly more than 3D7 parasites. Overall, this indicates that the smaller 3xHA tag has less effect on the parasite, than the larger 2x-FKBP-GFP-2xFKBP tag (see also new Figure 1L, showing a correlation of level of inactivation and the endocytosis phenotype for MyoF).

22) Line 212: The overlap of K13 with MyoF in Figure 2C 3rd panel (1st trophozoite panel) is not obvious, especially as the MyoF signal seems inexistant. I would advise the authors to replace with a better image. Also, why are there no images of schizonts shown in Figure 2C?

As suggested we exchanged the trophozoite image of panel Figure 2 C (now Figure 1C) and expanded this panel with images covering the complete asexual development cycle including schizonts in response to this and the previous points. As indicated above (point 20), schizont stages are complex to interpret. While late schizonts likely are not very relevant for endocytosis this is the first description of the location of the protein in this parasite and we therefore now provide a more thorough representation of the MyoF location across asexual stages in Figure1C and S2A.

23) Line 217: the spatial association of MyoF with K13 is very different when it is tagged with GFP and when it is tagged with 3xHA. The way the authors word it here, it seems that there is agreement with the two datasets, when this is not in fact the case (59% overlap for MyoF-GFP and only 16% overlap with MyoF-3xHA). These data suggest that the GFP and the multiple FKBP tags are doing something to the protein and therefore maybe the ensuing results using this line should not be trusted or be taken with a pinch of salt.
We agree with the reviewer that the location of this MyoF-GFP in the cell might differ due to the partial inactivation but in contrast to this comment, the data does not indicate any large differences. It seems the reviewer mixed something up (the 59% mentioned might come from the MCA2 figure?). The data with the two lines with differently tagged MyoF co-localised with K13 are actually quite comparable: GFP-tagged vs HA-tagged MyoF overlapping with K13 was 8% vs 16% full overlap, 12% vs 19% partially overlapping foci, 36% vs 63% foci that were touching but not overlapping (compare what now is Figure 1D and Figure S2C). Only in the 'no overlap' there is a much smaller proportion in the HA-tagged line. However, given that these are IFAs which on the one hand are more sensitive to see small protein pools but on the other hand also have pitfalls due to fixing of the cells (e.g. tiny increase in focus size due to fixing could increase the number of touching foci that in live cells might be close but did not touch), some variation can be expected to the live cells. We agree though that the partly reduced functionality of MyoF might be the reason for the consistent tendency of a lower overlap even though the difference is much less than indicated in the comment. We added *"with a tendency for higher overlap with K13 which might be due to the partial inactivation of the GFP-tagged MyoF"* to the sentence "IFA confirmed the focal localisation of MyoF and its spatial association with mCherry-K13 foci"
While we expect the fact that the difference between these parasites is only small somewhat reduces the "pinch of salt" with the MyoF line, we do agree that the partial functional inactivation of the GFP-tagged MyoF line may have some impact. However, we do not think that this means the results with the MyoF-GFP line are untrustworthy. On the contrary, it provides insights into its function that in some ways is equivalent to a knock down or TGD. Overall all the MyoF lines show: few vesicles occur in the MyoF-HA-line, more in the MyoF-GFP line and even more after knock sideways of MyoF-GFP. Importantly the severity of this phenotype correlates with the growth rates in these lines. Hence, together with the bloated food vacuole assays, this provides consistent data indicating that MyoF has a role in the transport of HCC to the FV and its level of activity correlates with the number of vesicles and growth. To better highlight this, it is now summarised in Figure 1M.

24) Line 219: the authors state here that they could not detect MyoF-GFP in rings, when in Figure 2C they show MyoF-GFP in rings, and also show that they could detect MyoF in Sup Fig. 3B with the 3xHA tagged line. Is this a labelling mistake in Figure 2C? If the authors could indeed not see MoyF-GFP in rings, this statement should have been made when Figure 2A was presented, and not so late in the manuscript, which causes confusion.

We thank the reviewer for pointing this out. We now provide a detailed time course (see also previous points) which shows that there is no detectable MyoF-GFP signal during ring stage development until the stage where the parasites starts the transition to trophozoites (i.e. MyoF-GFP signal could only be observed in parasites already containing hemozoin). In addition to the extended time course in Figure 1C (previously 2C) we included a panel of example ring stage images below to further highlight this. We also changed the labelling of the parasite with MyoF-GFP signal the reviewer mentions in Figure 1C to "late ring stage" (it already contains hemozoin) to clarify this.

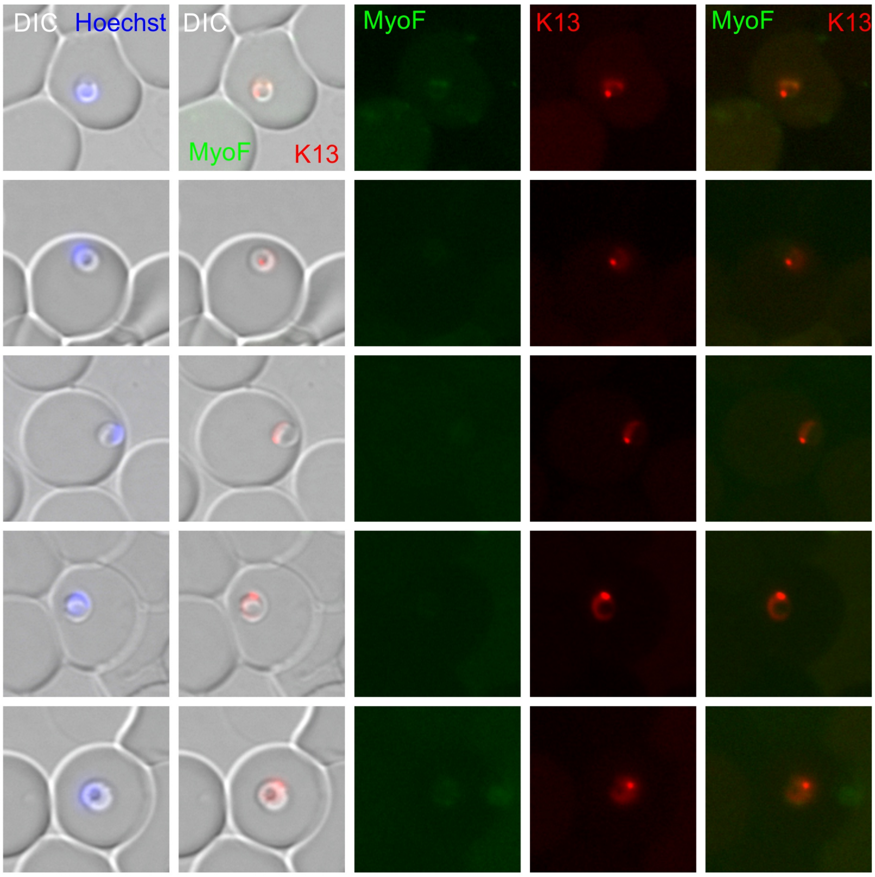

The description of Figure 1A is now changed to: (line 153) *"The tagged MyoF was detectable as foci close to the food vacuole from the stage parasites turned from late rings to young trophozoite stage onwards, while in schizonts multiple MyoF foci were visible (Figure 1A, S2A)."* Please see our answer to major comment #45 where we provide an explanation for the difference between MyoF-3xHA and MyoF-GFP signal in ring stage parasites.

25) Line 237: Showing a DNA marker (DAPI, Hoecht) for Figure 2E, and subsequent figures using mislocalisation to the nucleus, would help the reader assess efficiency of the mislocalisation.
Please see response to major comment #64 for a detailed answer on why we did not include DNA staining in the imaging used to assess mislocalization upon knock-sideways.

26) Line 254-256: authors should show the results of the bloating assay for parental 3D7 parasites (+ and - rapalog) to see whether the MyoF line - rapalog has increased baseline bloating. This applies to all subsequent FV bloating assays.
We did do several controls for bloated assays (including +/- rapalog of an irrelevant knock sideways line as well as using a chemical insult for which the control was 3D7 without treatment) in previous work (Birnbaum *et al.*, 2020), which indicated that there is no effect of rapalog to reduce bloating. Although these controls are more stringent, we nevertheless did a 3D7 +/- rapalog control and added this to the manuscript (Figure S2I). As it is not possible to

do this side by side with the assays that are already in the manuscript and the +/- rapalog 3D7 cells consistently showed no or very low numbers of cells without bloating (and stringent controls in the past equally did not show an effect), we believe adding this control once suffices.

27) Line 254-257: The authors say that because fewer parasites show a bloated food vacuole upon inactivation of MyoF it means that less hemoglobin reached the food vacuole. I understand the authors statement, however, shouldn't they look at the size of the food vacuole, instead of the number of parasites with bloated FV, to make such a statement? This has been done for KIC12 so why not doing it for MyoF?
This was now done and is provided as Figure 1J-K, S2J. The results confirm the assessment scoring bloated vs non-boated food vacuoles.

28) Line 259-261: these results would be difficult to interpret namely because the authors have dying parasites, which is exacerbated with the protein being knocked sideways. The authors should mention the pitfalls their knock sideways and tagging design here. Line 260-261: RSA is an assay relying on measuring parasite growth 1 cycle after a challenge with ART for 6 hours.
Fortunately, this concern is unfounded, as the survival (measured by parasitemia after one cycle) of the same sample + and - DHA is assessed, isolating the DHA effect independent of potential growth defects which are cancelled out. Hence, if there were parasites dying in the MyoF line (please note that they might not actually die, but simply grow more slowly), this factor applies for both the + and - ART condition. As we are testing for a decreased susceptibility to ART which would manifest as an increased survival in RSA surfacing above 1%, antagonistic effects of reduced MyoF function and ART treatment would not result in detectable differences as without effect, the RSA survival is always close to zero.
The same applies for the knock sideways where we assess the survival of +rapalog between +ART and -ART. If the reduced MyoF activity of the knock sideways leads to a decreased survival, this applies to both +ART and -ART. Please also note that rapalog was lifted after the DHA pulse (see e.g. Figure S2K).
That effects on growth are cancelled out is nicely illustrated for proteins where there is a stronger and more rapid effect on growth upon their conditional inactivation. For instance when KIC7 is knocked aside, there is a considerable increased of RSA survival, even though continued inactivation of KIC7 would have a severe growth defect (Birnbaum *et al.*, 2020). Vice versa, a growth defect alone does not result in reduced RSA susceptibility as evident from knock sideways of an unrelated protein or using a chemical insult (Figure 4H in (Birnbaum *et al.*, 2020) or simply slowing the ring stage by e.g. reducing EXP1 levels (Mesén-Ramírez *et al.*, 2019). Hence, a growth reduction is not expected to alter the RSA outcome. And even if it did, it would only lead to an underestimation of the readout if growth is too severely affected (which would be obvious in the + rapalog without DHA sample, which was not the case).
In that respect it is valuable to have the rapid kinetics of knock sideways which permit inactivation of a protein before severe growth defects occur (although the only partial responsiveness of MyoF clearly is not the most optimal). In contrast, the absolute loss of a gene (as is the case if diCre is used) prevents (or at least makes it extremely difficult as the timing would need to exactly hit sufficient protein reduction without killing the parasite until the end of the RSA) using this system in these experiments (again see (Mesén-Ramírez *et al.*, 2021) where in a EXP1 diCre based knock out RSA was only possible because we

complemented with a lowly, episomally expressed EXP1 copy to have parasites with only a partial phenotype to do this assay).

29) Line 261-263: the authors sate that MyoF has a function in endocytosis but at a different step compared to K13 compartment proteins. I am not sure what they mean here. Can this be clarified?

The different steps in endocytosis are explained in the introduction and we now tried to further clarify this (line 98). *"So far VPS45* (Jonscher *et al.*, 2019)*, Rbsn5* (Sabitzki *et al.*, 2023)*, Rab5b* (Sabitzki *et al.*, 2023)*, the phosphoinositide-binding protein PX1* (Mukherjee *et al.*, 2022)*, the host enzyme peroxiredoxin 6* (Wagner *et al.*, 2022) *and K13 and some of its compartment proteins (Eps15, AP2µ, KIC7, UBP1)* (Birnbaum *et al.*, 2020) *have been reported to act at different steps in the endocytic uptake pathway of hemoglobin. While inactivation of VPS45, Rbsn5, Rab5b, PX1 or actin resulted in an accumulation of hemoglobin filled vesicles* (Lazarus *et al.*, 2008; Jonscher *et al.*, 2019; Mukherjee *et al.*, 2022; Sabitzki *et al.*, 2023)*, indicative of a block during endosomal transport (late steps in endocytosis), no such vesicles were observed upon inactivation of K13 and its compartment proteins* (Birnbaum *et al.*, 2020)*, suggesting a role of these proteins during initiation of endocytosis (early steps in endocytosis).*"

VPS45 has not apparent spatial connection to the K13 compartment but the fact that MyoF does - and its inactivation also results in vesicle accumulation - indicates that it is downstream of vesicle initiation, providing the first connection from the initiation phase to the transport phase. More evidence for these different steps of endocytosis has been published in a recent preprint from our lab, where we simultaneously inactivated a protein of both "endocytosis steps" (Sabitzki *et al.*, 2023).

To clarify this in the results as requested, we changed the statement to: (line 256) *"Overall, our results indicate a close association of MyoF foci with the K13 compartment and a role of MyoF in endocytosis albeit not in rings and at a step in the endocytosis pathway when hemoglobin-filled vesicles had already formed and hence is subsequent to the function of the other so far known K13 compartment proteins."*

30) Do the authors mean that it is involved in endocytosis but not in ART resistance? If so, this is a very difficult statement to make since the parasites are dying. Is there any evidence of point mutations in MyoF in the field?

We split this point to address all issues raised here. Please see response to point 29 which clarifies that this was meant in a different way and our response to point 28 which explains why the dying parasite issue is not expected to affect the RSA (please also note that we do not have evidence of actually dying parasites in the MyoF-2xFKBP-GFP-2xFKBP line, most likely the growth is slowed).

The mutation issue is interesting. In fact evidence exists that MyoF mutations may be associated with resistance (Cerqueira *et al.*, 2017) (please note that there it is still called MyoC) but in a recent preprint from our lab we did not find any evidence for a significantly changed RSA survival in 12 tested mutations in the corresponding gene (Behrens *et al.*, 2023).

To clarify this we added the following statement to the discussion (line 709): *"Of note, mutations in myoF have previously been found to be associated with reduced ART susceptibility* (Cerqueira *et al.*, 2017)*, but 12 mutations tested in the laboratory strain 3D7 did not result in increased RSA survival* (Behrens *et al.*, 2023).

31) Line 298: the authors state that there is no growth defect in the first cycle when rapalog is added to the KIC11 line, however based on Figure 3D, there is evidently a 25% reduction in

growth compared to - rapalog at day 1 post treatment, and a 60% reduction by day 2, which is still within the 1st growth cycle. The authors should either revise their statement or provide an explanation for these findings. The authors should also explain why their Giemsa data in Fig. 3E is not in accordance with their FACS data.
We think there is a misunderstanding here, as our figure legend was not detailed enough and we apologise if this had been misleading. The growth effect is restricted to invasion or possibly the first hours of ring stage development (see point 4&5, reviewer 2), which in asynchronous cultures more rapidly takes effect as the culture also contains schizonts that immediately generate cells that re-invade but can't due to inactivation of KIC11 (due to the rapid action of the knock sideways, KIC11 is already inactivated). In contrast, in highly synchronous cultures, this effect can only be evident once the parasites reached the schizont stage (starting with rings this takes close to 2 days). We now clarify that Figure 2E (previously Figure 3D) shows growth data obtained with an asynchronous parasite culture, while in Figure 2F the growth assay is performed with tightly synchronized (4h window) parasites as stated in the Figure legend.

We now explicitly state in each Figure legend and for each growth experiment throughout the manuscript whether we used asynchronous or synchronized parasites for growth assays.

Related to this, the incorrect y-axis label of what is now Figure 2E mentioned in major comment #58 is now corrected.

32) Line 301: KIC11 could also be important very early for establishment of the ring stage for example for establishment of the PV. Also, was mislocalisation assessed in rapalog-treated parasites at 72 hours or in cycle 3?
This is a valid point and this has now been addressed. We performed an invasion/egress assay revealing similar schizont rupture rates, but significantly reduced numbers of newly formed ring stage parasites (Figure 2H, S3G), indicating an effect of KIC11 inactivation either on invasion or possibly the first hours of ring stage development. A very similar point was raised by Reviewer 2, please see reviewer 2; major comment #4. This is now also reflected in line 302, which now reads: *"… indicating an invasion defect or an effect on parasite viability in merozoites or early rings but no effect on other parasite stages (Figure 2F-H, Figure S3F-G)."*

We further included an assessment of mislocalization 80 hours after the induction of knock-sideways by addition of rapalog in Figure S3E which showed mislocalization of KIC11 to the nucleus.

33) Line 311: the authors should change the sentence from 'not related to endocytosis' to 'not related to endocytosis or ART resistance'.
Done as suggested.

34) Line 323-325: Authors say that a nuclear GFP signal can be observed in early schizonts for KIC12. According to the pictures provided in Figure 4A and Figure S5A it is not very obvious. Also faint cytoplasmic GFP signal could only be background as we can see that exposure is higher for schizont pictures
We changed the sentence (line 339) to: *"…nuclear signal and a faint uniform cytoplasmic GFP signal was detected in late trophozoites and early schizonts and these signals were absent in*

later schizonts and merozoites (Figure 3A, Figure S4A,B)." in order to emphasize that the nuclear signal disappears early during schizont development.

35) Line 326-328: The authors say that kic12 transcriptional profile indicate mRNA levels peak (no s at peak) in merozoites. Should they show live cell imaging of merozoites then? Because from the Figure 4A schizont pictures where schizonts are almost fully segmented no signal can be observed.

The observation that mRNA levels of early ring stage expressed proteins tend to increase already in mature schizonts and merozoites is well established (e.g. (Bozdech *et al.*, 2003)). A very good example for this are exported proteins of which most show a transcription peak in schizonts but the proteins are only detected in rings see e.g. (Marti *et al.*, 2004). Hence, our observation for KIC12 is quite typical.

We originally did not include merozoites, as in the last row of Figure 3B fully developed merozoites within a schizont with already ruptured PVM are shown and no GFP signal can be detected in these parasites. We now provide images of free merozoites in Figure S4A-B showing again no detectable GFP signal.

We thank the reviewer for pointing out the typo, "peak" has been corrected.

36) Line 347: The authors state that using the Lyn mislocaliser the nuclear pool of KIC12 is inactivated by mislocalisation to the PPM. This tends to suggest that only the nuclear pool of KIC12 is mislocalised. How is it possible that only the nuclear pool is mislocalised?

The Lyn mislocaliser is at the PPM which is continuous with the cytostomal neck where the K13 compartment likely is found. The effect of the Lyn mislocalizer on the KIC12 protein pool localizing at the K13 compartment is therefore somewhat unclear. For this reason we already had the following statement in the original submission (line 400): "*Foci were still detected in the parasite periphery and it is unclear whether these remained with the K13 compartment or were also in some way affected by the Lyn-mislocaliser."* We would like to stress here that the same does not apply to the nuclear mislocaliser, which is only a trafficking signal delivering KIC12 to the nucleus and hence likely does not affect the nuclear pool of KIC12, only the K13 compartment pool (the main interest of this manuscript).

We realised that the statement towards the end of this paragraph was unnecessarily ambiguous in regards to the K13 compartment pool of KIC12 which might have caused some confusion about the function of this pool of KIC12 and therefore modified it to (line 374): *"Due to the possible influence on the K13 compartment located foci of KIC12 with the Lyn mislocaliser, a clear interpretation in regard to the functional importance of the nuclear pool of KIC12 other than that it confirms the importance of this protein for asexual blood stages is not possible. In contrast, the results with the nuclear mislocaliser indicate that the K13 located pool of KIC12 is important for efficient parasite growth.".* It is also important to note that this limitation does not apply to the NLS knock sideways in regard to the K13 compartment and that the endocytosis function of this pool of KIC12 seems solid which with this statement is enforced.

37) Line 368-369: Effect was also only partial for MyoF. Why didn't you measure the same metrics for MyoF?

This was now done and is provided as Figure 1J-K, S2J, confirming our previous interpretation, see also point #27 which raises the same point.

38) Line 379: you don't know if all proteins acting later in endocytosis will have an increased number of vesicles as a phenotype
This is based on our current definition as stated in the introduction. It assumes a directional vesicular transport of hemoglobin to the food vacuole where inhibition of early stages will prevent transport before HCC-filled autonomous vesicular containers have formed and entered the cell. In contrast later inhibition stops such containers from further transport, leading to their accumulation. Such an accumulation is visible after VPS45-inactivation and other proteins (Jonscher *et al.*, 2019; Mukherjee *et al.*, 2022; Sabitzki *et al.*, 2023) or treatment with cytochalasin D (Lazarus *et al.*, 2008). While it is possible that there may be smaller intermediates formed at the K13 compartment that later on unite or fuse with the compartment evident after VPS45 inactivation and these might be missed due to small size (i.e. inhibition of a step between K13 compartment and an early endosome or equivalent), this would still be upstream of the VPS45 induced containers and hence would be earlier. We therefore believe that based on the framework given in the introduction (see also (Spielmann *et al.*, 2020)) to assume that a phenotype manifesting as reduced food vacuole bloating without formation of detectable vesicles likely signifies inhibition of the process early whereas reduced bloating but with vesicles signifies inhibition later in the process.

39) Line 413-414: The authors state that no growth defect was observed upon KS of 1365800. Is growth alone enough to say that there is no impact on endocytosis?
This is an interesting point. The endocytosis proteins we studied so far indicate that efficient impairment of endocytosis manifests as a severe growth defect. Hence, lack of a growth defect can be assumed to be an indicator for absence of an important role for endocytosis (or any other growth relevant process). Clearly there is a gradual response, such as seen in the different MyoF versions resulting in proportional growth and vesicle appearance phenotypes. Hence, a protein with a minor role might have slipped our attention but then it probably is also not a very important protein in endocytosis.
To further strengthen our assessment of PF3D7_1365800 importance for asexual blood stage development, we now also generated a cell line expressing the PPM Mislocalizer, enabling knock sideways to the PPM. This was done because this protein consistently has a focus at the nucleus that may be within the nucleus. Again this revealed no growth defect upon inactivation (Figure S7D).

40) Line 432: in this section, the authors state that KIC4 and KIC5 seem to have domains that may suggest these proteins are involved in endocytosis, based on the alpha fold data that is publicly available. Considering the authors have TGD-SLI versions of these lines (Birnbaum et al. 2020) and have already confirmed in this previous publication that they confer resistance to ART; it would make sense to look at endocytosis for these genes. This would be a relatively simple and straightforward experiment, taking no longer than two to three weeks, and would require no additional reagents or line generation. Doing these experiments would add a lot more weight to this final section. The authors later state that KIC4 and 5 are TGD lines, so not the best for endocytosis assays. It is unclear why this would be difficult to do if an adequate control is contained in the experiment (such as parental 3D7). It explains why they did not perform the MCA2 endocytosis assays further up, but in my opinion, an attempt at doing these

assays is important and would significantly increase the impact of this paper. Identical as major comment #17.
As stated in the manuscript and above, we were originally hesitant to do these assays due to the fact that we can't induce inactivation which is less ideal than comparing the identical parasite population split into plus and minus and is further complicated by the likely smaller effect as the TGDs still permitted growth. However, we see the point of the reviewer and now performed these assays using 3D7 as controls and taking extra care to account for stage differences between the TGD lines and 3D7. However, there was no significant difference in the bloated food vacuole assays with these cell lines. Due to the reasons mentioned in major point 17, we are not sure this indeed means these proteins have no role in endocytosis. One possible reason why we were able to obtain these TGDs may have been because the effect on endocytosis is less than in the essential proteins (or is ring stage specific) and in a TGD an endocytosis defect may therefore not be detectable with our assays (see details and further possible explanations in response to point 17).
In an attempt to address the TGD issue, we generated knock sideways cell lines for KIC4 and KIC5. Unfortunately, the mislocalization of KIC5 to the nucleus was inefficient (see figure below). As this did not result in a growth defect (in contrast to the clear KIC5-TGD growth defect (Birnbaum *et al.*, 2020)), this line is not suitable to study a potential role of this protein in endocytosis. Therefore, we performed the bloated food vacuole assay only with KIC4-2xFKBP-GFP-2xFKBP^{endo}+1xNLSmislocaliser parasites. However, this revealed no effect on HHC uptake, which is in line with the normal growth of KIC4-TGD parasites (Birnbaum *et al.*, 2020) and suggests that this protein could only have a minor or redundant role in endocytosis (it is the line that shows the smallest effect in RSA). As the KIC4 and KIC5 knock sideway lines did not permit any conclusions, we did not include them into the revised manuscript but they can be found here:

Review COMMONS

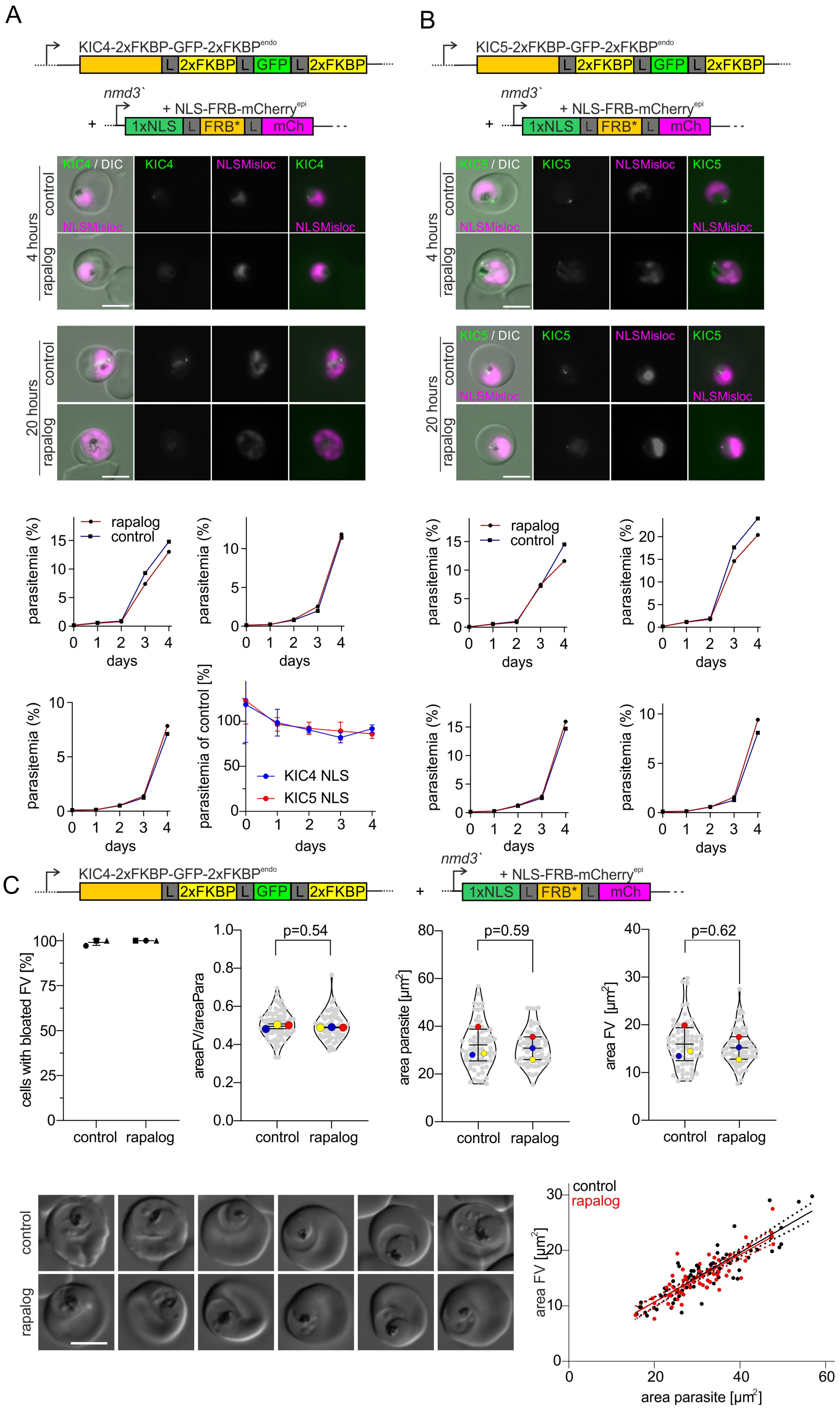

Figure legend: (A) Live-cell microscopy of knock sideways (+ rapalog) and control (without rapalog) KIC4-2xFKBP-GFP-2xFKBP^{endo}+ 1xNLS mislocaliser parasites 4 and 20 hours after the induction of knock-sideways by addition of rapalog. Scale bar, 5 μm. Relative growth of asynchronous KIC4-2xFKBP-GFP-2xFKBP^{endo}+1xNLSmislocaliser plus rapalog compared with control parasites over five days. Three independent experiments were performed. Growth of knock sideways (+ rapalog) compared to control (without rapalog) KIC4-2xFKBP-GFP-2xFKBP^{endo}+1xNLSmislocaliser (blue) or KIC5-2xFKBP-GFP-2xFKBP^{endo}+1xNLSmislocaliser (red) parasites over five days. Mean relative parasitemia ± SD is shown. **(B)** Live-cell microscopy of knock sideways (+ rapalog) and control (without rapalog) KIC5-2xFKBP-GFP-2xFKBP^{endo}+1xNLSmislocaliser parasites 4 and 20 hours after the induction of knock-sideways by addition of rapalog. Scale bar, 5 μm. Growth of asynchronous KIC5-2xFKBP-GFP-2xFKBP^{endo}+ 1xNLSmislocaliser plus rapalog compared with control parasites over five days. Four independent experiments were performed. **(C)** Bloated food vacuole assay with KIC4-2xFKBP-GFP-2xFKBP^{endo}+1xNLSmislocaliser parasites 8 hours after inactivation of KIC4 (+rapalog). Cells were categorized as with 'bloated FV' or 'non-bloated FV' and percentage of cells with bloated FV is displayed; n = 3 independent experiments with each n=19-30 (mean 21.4) parasites analysed per condition. Representative DIC are displayed. Area of the FV, area of the parasite and area of FV divided by area of the corresponding parasites were determined. Mean of each independent experiment indicated by coloured symbols, individual datapoints by grey dots. Data presented according to SuperPlot guidelines (Lord *et al.*, 2020); Error bars represent mean ± SD. P-value determined by paired t-test. Area of FV of individual cells plotted versus the area of the corresponding parasite. Line represents linear regression with error indicated by dashed line.

41) Line 490-493: the authors state that the K13 compartment proteins fall in two groups, some that are involved in ART resistance AND endocytosis, and some that have different functions. However, in this manuscript the authors have demonstrated 3 flavours that K13 compartment proteins can come in:
• Some that confer ART resistance and are involved in HCCU (MCA2)
• Some that are involved in HCCU but not ART resistance (MyoF & KIC12)
• Some that are involved in neither (KIC11)
The authors should therefore revise this statement.

We agree that this was not well phrased. To account for the fact that not all endocytosis proteins confer increased RSA survival to the parasites when inactivated we changed this statement (line 604): *"This analysis suggests that proteins detected at the K13 compartment can be classified into at least two groups of which one comprises proteins involved in endocytosis or in vitro ART resistance whereas the other group might have different functions yet to be discovered."*

Generally, we believe that endocytosis is the overarching criterion and we therefore would like to keep the definitions of the main groups (endocytosis or not). As indicated by the title, the focus of the manuscript is on the K13 compartment for which so far endocytosis is the only experimentally associated function. That this group contains proteins that do not confer reduced ART susceptibility when conditionally inactivated (KIC12 and MyoF) is explained by their stage-specificity, making this a subgroup of the overarching endocytosis group.

We realise that with the endocytosis data on the KIC4, KIC5 and MCA2 TGD there is now also a subgroup we were unable to demonstrate an endocytosis effect in trophozoites although they show changes in RSA survival. However, as indicated above, we would be hesitant to fully exclude some role of these proteins in endocytosis in rings. Particularly as a comparably small reduction in endocytosis protein activity or abundance is sufficient to increase RSA survival (Behrens *et al.*, 2023). A principal classification of "endocytosis or ART resistance" or "neither endocytosis nor ART resistance" still accounts for this and therefore seems to us to

be the most useful, particularly also in light of our domain identification that then can be linked with one or the other group.

42) Line 508: the authors state that they expanded the repertoire of K13 compartments, when in fact they functionally analysed them - they did not do another BioID to identify more candidates.
We respectfully disagree with the reviewer in this point, we did expand the repertoire of known K13 compartment proteins. Only independently experimentally validated proteins from proximity biotinylation experiments can be considered part of the K13 compartment (or any other cellular site or complex). Without validation of the location, the identified proteins can only be considered candidates. This is highlighted in this manuscript by the finding that several proteins of the list did not localize at the K13 compartment.

43) Line 570-572: has anyone ever tested whether CytoD or JAS treatment in rings, is sufficient to mediate ART resistance? Something similar to what was done in PMID 21709259 with protease inhibitors. If not this would be a pretty interesting experiment for the authors to do that could shed more light on the MyoF data. It would take maybe 2 weeks to do and not require the generation of any new lines. This would clarify whether other Myosins other than MyoF are involved in endocytosis, as is suggested by previous publications (PMID: 17944961).
We now included this experiment. In agreement with a lacking need of MyoF in rings and no effect on RSA survival, there was no increased survival of the parasites in RSA (neither on 3D7 nor on K13 C580Y parasites) after cytD treatment (new part in Figure 1M). We thank the reviewer for pointing out that this experiment might also inform on whether other myosins influence endocytosis in ring stages. We added (line 250): *"Similarly, also incubation with the actin destabilising agent Cytochalasin D* (Casella *et al.*, 1981)*, had no effect on RSA survival in 3D7 or K13^{C580Y}* (Birnbaum *et al.*, 2020) *parasites, indicating an actin/myosin independent endocytosis pathway in ring stage parasites (Figure 1M) and speaking against other myosins taking over the MyoF endocytosis function in rings."*

44) Line 608: inhibitors targeting the metacaspase domain of MCA2 may inadvertently inactivate other essential parts of the protein. They authors should acknowledge this possibility in the text.
The inhibitors used in the cited studies (Kumari *et al.*, 2018) are validated metacaspase inhibitors, such as Z-FA-FMK (Lopez-Hernandez *et al.*, 2003). Activity against the other parts of PfMCA2 - which apart from the MCA domain shows no homology to other proteins - is therefore unlikely.

45) Line 624-625: the authors state that MyoF is 'lowly expressed in rings' - indeed this is the case in their MyoF-2xFKBP-GFP-2xFKBP line which the authors established has defects due to the tag, but it appears from their MyoF-3xHA tagged line that it is expressed in rings. The authors should therefore revise their statement, and be careful of making claims based on their defective line and using fluorescence imaging as their only metric. If they do want to make the statement that it is not there in rings, they should also do a western blot, which is much more sensitive since it amplifies the signal compared to an image of one parasite.

This comment is related to major point #24. We also would like to stress that while the MyoF-GFP line already shows a phenotype, the impression of defectiveness based on its location is due to a mix up (see major point #23).

We now provide a comprehensive time course of the MyoF-GFP signal (Figure 1C, S2A) showing that there is no detectable MyoF-GFP signal until the transition from ring to trophozoite stage. As this is all under the endogenous promoter, we do not think the partial functional inactivation of the tagging is the reason for the absence of the signal. If anything, we would have expected adding a stably folded structure such as GFP to increase the stability of the protein. The main reason for the discrepancy of MyoF signal in rings between the GFP-tagged line (of note there is also no detectable MyoF-GFP signal in MyoF-2xFKBP-GFP ring stage parasites (Figure S2B)) and the HA-tagged line likely is that IFA is much more sensitive than live GFP detection (similar to the high sensitivity the reviewer mentions in regards to WB). This discrepancy therefore is likely due to the fact that the lowly expressed MyoF only become apparent with the HA-tagged line due to the IFA. We therefore believe that MyoF is 'lowly expressed in rings' is an appropriate description of our results obtained with three different cell lines (MyoF-2xFKBP-GFP-2xFKBP, MyoF-2xFKBP-GFP and MyoF-3xHA). We hope this is sufficiently well reflected in the manuscript where we write ‘a low level of expression of MyoF in ring stage parasites.’ not that it is ‘not there in rings’ (line 174).

46) Line 635: arguably this is the 3rd variety and not the 2nd (the authors already mentioned 2 types - ones that are involved in HCCU AND ART and those involved in HCCU only). See comment for line 490-493 above.

See response for major comment #41, we now consistently used "or" instead of "and". See line 490-493 how this was resolved for what previously was line 635.

47) Line 785: Bloated food vacuole assay/E64 hemoglobin uptake assay method specify that a concentration of 33mM E64protease inhibitor was used. However, in reference 44, cited in the manuscript, a concentration of 33μM E64 was used. Please confirmed if this is just a typo or if 1000x E64 concentration was used which renders the experiment invalid.

We thank the reviewer for pointing this out, we corrected this typo and will look out for symbol font conversion errors for the resubmission.

48) Line 788: it is unclear from this section what is considered a bloated food vacuole - is there an area above which the FV is considered bloated? Do the authors do these measurements manually or use an addon in FIJI/ImageJ? What is the cutoff for if a FV is bloated? Please clarify. Additionally, for the representative images + rapalog for Figures 2H and 4H, it would be useful to see where the authors delineate the FV (add a white circle showing what is actually measured).

The bloated FV assay is well established (Jonscher *et al.*, 2019; Birnbaum *et al.*, 2020; Sabitzki *et al.*, 2023). Although the bloating of the FV is a human judgment call, it is actually quite obvious: bloating appears as an easily spotted bulging of the FV in DIC. As also minor bloating is scored as 'bloated', it is a very conservative assay. Using an-add on to measure this is not straight forward. It is unclear how this bulging effect of the FV in DIC could be spotted by a software and due to the obviousness to human operators, potentially lengthy and complicated efforts to design appropriate machine learning options were not undertaken. The situation faced by the scorer of the assay is evident from Figure S4F-G which contains close to 50 "on rapalog" cells and close to 50 control cells, giving representative cells from all replicas of bloated FV assays with KIC12. Please note that these images shows the most

complicated situation as far as bloated assays go, because the phenotype is not 100% (see Figure 3F) compared to e.g. KIC7 inactivation which leads to lack of bloating in almost all cells (see (Birnbaum *et al.*, 2020) Figure 3E) but nevertheless the difference is still obvious. We are aware that in such situations (less than absolute inhibition) this assay scoring of "yes" or "no" is a surrogate for the actual level of inhibition and may be more subjective. This is why in this case we also did the FV size measurements (which are less dependent on human judgment) to further support this and give a better quantifiable measure. Of note, the bloated food vacuole judgments are done "blinded", i.e. the examiner does not know which sample they are looking at.
In response to this reviewer's point we now also added the FV size refinement of the assay for MyoF inactivation which is one of the cases where inhibition of bloating is not in 100% of the cells (see major comment #27). Please also note here the advantage of the rapidly acting knock sideways technique for these assays which shows the sum of effect 8 h after initiating inactivation and for which we carefully control size of the cells which shows that there is no significant growth reduction over the assay time, excluding secondary effects due to a generally reduced viability. Compared to slower acting systems suggested to have been used instead (see introductory part and significance of this review), the rapid speed of knock sideways reduces the risk of potential pleiotropic or compensatory effects due to the time needed for proteins to be depleted if the gene or mRNA is targeted instead.

The suggestion to include a 'white circle' (raised also as minor comment#27) is useful as an aid to see the food vacuole. However, in contrast to the Figures in (Birnbaum *et al.*, 2020) (where we did add such a circle), we here included the DHE staining images in the figure, labelling the parasite cytosol which readily shows the FV (the FV corresponds to the region where there is no DHE staining). As this shows the position of the FV we would prefer to not obscure the DIC images with additional features to permit the reader to see the difference between bloated or non-bloated food vacuoles and keeping the image as natural as possible.

49) Line 863-864: this sentence seems to be out of place.
We thank the reviewer for pointing this out, the details of nucleus staining were moved to the correct part.

50) Line 875: the authors state that there is a light blue wedge, when the circle consists of grey and black wedges. Please revise this.
This has been corrected.

51) Line 1059-1061: it is unclear whether the individual growth curves are different clones or whether they are just the same experiment repeated? If it is the latter, then why are they not combined, as is traditionally done?
These are the individual replicates of the growth curves shown in Figure 1G of the same cell lines done on a different occasion. We always try to show as much of the primary data as possible and believe that showing individual data points from the different experiments is better than only the combined values which obscure the actual course of each experiment.

52) Line 919-924: the authors mention a blue and red line, but there is only a black line in figure 3D. Moreover, the experiment of using the LYN mislocaliser was only done for KIC12 according to the manuscript. Additionally, the y axis of the figure states relative growth day 4[%] compared to rapalog, but then on the x axis there are several days. In the text it says

there is no growth defect until the second cycle, but from this graph it appears the growth defect is evident as early as 1 day post rapalog treatment. Can the authors please clarify and correct the issues pointed out.

We thank the reviewer for pointing this out, this was due to a copy & paste error in the figure legend that was now amended. We also fixed the incorrect axis label. For the last part (growth defect) please see detailed answer to Major comment#31 raising the same concern for KIC11 (in synchronous parasites the defect only takes effect once the cells reached the relevant stage whereas in asynchronous cultures there are always cells in the relevant stage that due to the rapid effect of the knock sideways already have a growth phenotype).

53) Figure 1 panel B & C: the label of the figure where the signal from MCA2Y1344STOP-GFP is shown with the DAPI signal overlayed is deceptive since it suggests that this is the signal of full length MCA2. Please change the label of this panel from MAC2/DAPI to MCA2Y1344STOP/DAPI. The same is true for Panel C for the image labeled MCA2/K13 - please change this to MCA2Y1344STOP/K13.

Done as requested.

54) Figure 2B: what stages are these parasites? Please state this in the figure. Based on the MyoF pattern, it looks like rings in the upper panel and trophs in the bottom pannel. Why were schizonts not shown?

Both are trophozoites (early trophozoite in top panel and late trophozoite in bottom panel). This is now labelled in what now is figure 1B. As stated above, schizont stages are less relevant for the topic of this manuscript and in order to prevent the manuscript from getting more disjointed and keeping it more focussed on the main topic, we decided to not include a schizont in the manuscript. Nevertheless, we included an example image below.

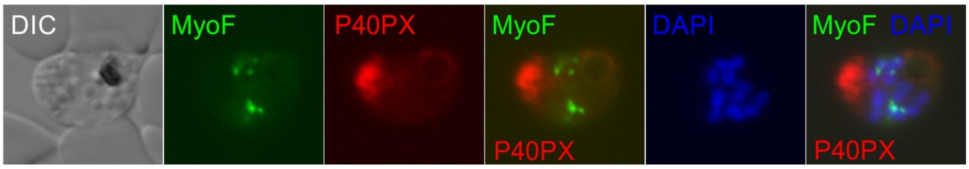

55) Figure 2D&F: it is not very meaningful when growth assays are shown as a final bar after 4 days of growth. It is much more useful and informative to see a growth curve instead (as is shown in the supplementary), since it shows if the defect is apparent in the first growth cycle or later. With the way the data is currently shown, this is not apparent. I would advise the authors to switch the graph in 2F out of a combined graph of all the biological replicates growth curves for S3D - showing error bars.

While we in principle fully agree with the reviewer in showing the course of the full experiment (which is available in Figure S2E), the key here is to show the overall difference. Hence, we would like to keep this comparison of the overall effect on growth in what now is Figure 1E and G. It is part of the argument to the doubts this reviewer raises to the function of MyoF (mainly in the overall assessment and the significance statement) to show that the phenotype is actually very consistent (partial inactivation through tagging or further inactivation using knock sideways increases endocytosis phenotypes, correlating with parasite viability).

Please also note, that the growth curves upon knock sideways shown in Figure 1G, S2E are performed with asynchronous parasite cultures, which doesn't allow us to draw direct conclusions about growth cycle effects.

Nevertheless, we now also included the suggested combined data representation in Figure S2E.

56) Figure 3: why were the calculation of FV area, parasite area and FV/parasite area only done for KIC12 and not done for MyoF? It would be interesting to see if any of these values are different for MyoF - whether the parasites are smaller in area and therefore FV smaller. Please present them Figure 2. Images should be already available and would not require further experiments to be done, only the analysis.
This now has been done (confirming our results) and is included as Figure 1J-K, S2J. This point was also raised as major comment #37, please also see detailed answer there.

57) Figure 3B: why is there no spatial association assessment for KIC11 and K13 as was done for the MCA2 and MyoF? The authors should show a pie chart showing the degree of association here as was done for the other proteins.
This is now included in Figure 2C.

58) Figure 3D: The y axis of the figure states relative growth day 4[%] compared to rapalog, but then on the x axis the experiment takes place over several days. Is this a typo in the y axis? Additionally, the authors state in line 287-290 that the growth defect upon addition of rapalog is only seen in the second cycle, but from this graph it appears the growth defect is already evident 1 day post rapalog addition. The figure legend also does not make sense for this figure since it mentions a blue and a red line, when there is only a black line present. The legend also mentions the LYN mislocaliser which was used for KIC12 not KIC 11 (see above).
We apologise for the inadequate legend and colour issues, this was amended. This point was also raised in major comment #31 and #52, please find detailed answer there.

59) Figure 3E: the colour for Control and Rapalog 4 hpi are very similar and very hard to discern. Please choose an alternative colour or add a pattern to one of the samples. The y axis is also missing a label. Is this supposed to be parasitemia (%)?
We thank the reviewer for pointing this out, the missing label is now included and the colour has been adapted to make them better distinguishable.

60) Figure 4A: the ring shown in this figure does not appear to be a ring (it is far too large and appears to have multiple nuclei?). Do the authors have any other representative images to show instead?
This is in fact a ring, but we realize that we accidentally included an incorrect size bar in the ring image of Figure 4A (now Figure 3A) (size bar for 63x objective instead of the correct one for the 100x objective), we apologise for this oversight. We don't think this parasite has multiple nuclei, instead the Hoechst signal shows the often elongated nucleus seen in rings that can appear as two foci in Giemsa stained smears which leads to the typical diagnostic feature of *P. falciparum* rings in diagnostics. In order to exclude any doubts about the nuclear localization of KIC12 in rings, we here attached a panel with more examples of KIC12-2xFKBP-GFP-2xFKBP ring stage parasites.

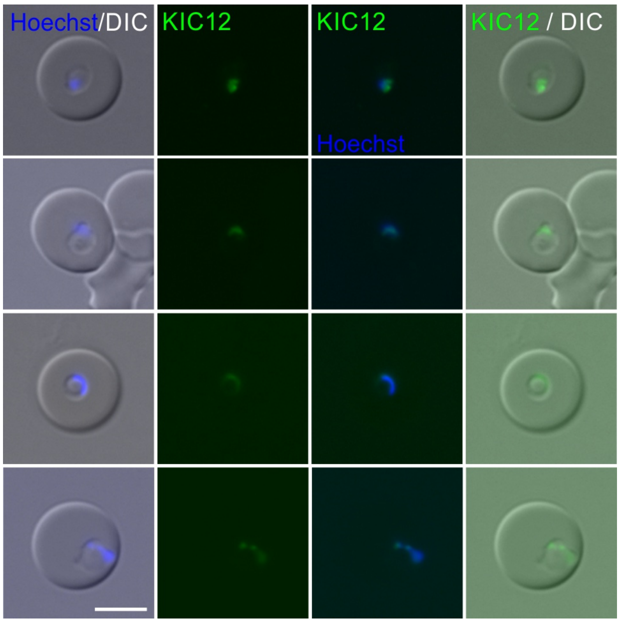

61) Figure 4B: why is there no spatial association assessment for KIC12 and K13 as was done for the MCA2 and MyoF? The authors should show a pie chart showing the degree of association here as was done for the other proteins. This should be done for the different life cycle stages considering the changing localisation of KIC12.
This is now provided in Figure S4A. As suggested by the reviewer, we independently quantified the association for ring stage, early trophozoite and late trophozoites stage. As there is no KI12 signal in schizonts, we did not include a quantification for this stage.

62) Figures 4C&E: it is extremely important to show the DNA stain in both these samples considering that a portion of KIC12 is in the nucleus! Please add the DAPI signal for these figures (as for all other figures!).
Please see major comment #64 for a detailed answer why we did not include DNA staining in the imaging used to assess mislocalization upon knock-sideways.

63) Figure 4E: this figure should be presented before 4D (considering the line being presented in 4E is used in an experiment in 4D). The authors should switch the order of these two.
We see the point the reviewer is raising here, Figure 4D (now Figure 3D) also contains the data with the Lyn mislocaliser while we first talk about the NLS mislocaliser. This permits a better comparison between the two mislocaliser lines. However, first explaining the Lyn-mislocaliser and then going back to the NLS would make it rather complicated for the reader to follow the storyline and therefore we would like to keep the order as it is. We realise that this means the reader has to go back one figure part for seeing the Lyn growth data, but believe this is worth the benefit that the data is there compared to the NLS result.

64) It is unclear why in many of the fluorescence images the authors do not show the DAPI signal - particularly when colocalising with K13 and when doing the knock sideways experiments. Please add these images to the figures - I would assume they have already been taken, so would simply involved adding the images to the panel.
We did not include DNA staining (DAPI or Hoechst) for any of the images used to assess the efficacy of mislocalization, as we would prefer to keep the parasites as representative of a viable parasites in culture as possible. Hence they were imaged without DNA stain (these stains are toxic). We would like to point out that a DNA stain is not necessary, as the mislocaliser already marks the nucleus (in the case of the NLS mislocaliser), actually even somewhat more accurately, as it fills the entire nuclear space rather than only the DNA which is marked by DAPI or Hoechst.
For LYN this admittedly is not the case, there the mislocaliser marks the plasma membrane. However, we think the proper control for efficient mislocalisation is the comparison between the GFP-tagged protein of interest and the mCherry mislocaliser to show mislocalisation, as

previously done in our lab (e.g. (Birnbaum *et al.*, 2017; Jonscher *et al.*, 2019; Birnbaum *et al.*, 2020)).
Due to their toxicity, we also avoided nuclear staining in some other parts of the manuscript when we were of the opinion that a nucleus signal was not necessary.

65) Throughout the manuscript, there is no western blot confirming the correct size of their modified proteins. This should be provided.
We did perform Western blot analysis for both MCA2 cell lines. MCA2 is the only gene-product for which we generated a disruption for this work, and together with the severe truncation from previous work, we provided a Western blot-based confirmation of the correct size.

The MCA2 disruptions are at least partially dispensable for *in vitro* parasite growth, hence if degradation occurred, this might not have been noticed. In that case we considered it relevant to show that the truncations were of the expected size. The other proteins in the main figures are essential for growth. Hence, if the tagging approach would lead to unexpected changes in protein integrity (which we assume is what was intended by this concern to be assessed with a Western blot), the parasites expressing the tagged MyoF, KIC11 and KIC12 would - due to their importance for asexual blood stage development - not have been obtained. Hence, we can assume the integrity of the tagged protein is very unlikely to have been affected in a functionally relevant way.

66) None of the figures are appropriate for individuals with colour blindness, limiting their accessibility to the paper. Please change the colour schemes for all fluorescent images using magenta/green or an alternative colour combination appropriate for colourblind individuals.
We thank the reviewer for this comment. This has now been amended, individual channels of fluorescence microscopy images are now shown in greyscale, while the overlay was changed to green/magenta.

Minor Comments

1) line 29: remove 'are'.
Done.

2) Line 29: the text says "HCCU is critical for parasite survival but is poorly understood, with the K13 compartment proteins are among the few proteins so far functionally linked to this process." The sentence should be: 'HCCU is critical for parasite survival but is poorly understood, with the K13 compartment proteins among the few proteins so far functionally linked to this process."
Done.

3) line 44: remove 'the'
Done.

4) Line 48: consider mentioning here that malaria is caused by the parasite Plasmodium - otherwise the first mention of parasite in line 52 is confusing for the non-specialist reader.
Done.

5) Line 49: estimated malaria-related death and case numbers are from the 2021 WHO World malaria report. You cite the 2020 WHO World malaria report.
We now cite the newest WHO report.

6) Line 53: please insert the word 'have' between now and also.
Done.

7) Line 54: please change 'was linked' to is linked
Done

8) Line 72: I would specify that free heme is toxic to the parasite. Especially as you mention that hemozoin is nontoxic.
Sentence would be "where digestion results in the generation of free heme, toxic to the parasite, which is further converted into nontoxic hemozoin"
Done.

9) Line 90: authors should either say "in previous works" or "in a previous work"
The text has been altered to say: “ in a previous work”.

10) Line 91: "We designated these proteins as K13 interaction candidates (KICs)"
Done.

11) Line 95: please change 'rate' to number
Done.

12) Line 109: Please include a coma before (ii).
Done.

13) Line 112: as shown by Rudlaff et al in the paper you are citing, PPP8 is actually associated with the basal complex. You can say that "(ii) were either linked or had been shown to localise to the inner membrane complex (IMC) or the basal complex (PF3D7...).
Done.

14) Line 114: Protein PF3D7_1141300 is called APR1 in the manuscript but ARP1 in Supplementary Table 1. Please correct.
Done.

15) Line 131: please define SNP - this is the first use of the acronym.
Done.

16) Line 133-134: South-East Asia instead of "South Asia"
Done.

17) Line 135: please explain what TGD is - it is referred to over and over again in the manuscript without ever being explained.
We apologise for this oversight. We now explain what is meant with TGD at the suggested point of the manuscript.

18) Line 145: change 'Western blot' to western blot - only Southern blot is capitalised since it is named after an individual, while the other techniques are not.
To the best of our knowledge this issue has not been resolved, some Journals capitalize the "W" (e.g. Science), while others don't (e.g. Nature). We would prefer to continue to capitalize the "W", as this is consistent with the original publication from (Burnette, 1981), but if there are strong objections, we would be happy to change this.

19) Line 152: add "the" between 'and spatial'
Done.

20) Line 158: please define SLI as selected linked integration, since it is the first use of the acronym.
Done.

21) Line 178: introduce a coma after protein. Sentence should be "Proliferation assays with the MCAY1344STOP-GFPendo parasites which express a larger portion of this protein, yet still lacking the MCA domain (Figure 1), indicated no growth ...
Done.

22) Line 195: the authors could mention that MyoF was previously called MyoC in the Birnbaum 2020 paper. I wanted to check back in the Birnbaum 2020 paper and could not find MyoF
Good point, this was done.

23) Line 200: "Expression and localisation of the fusion protein was analysed by fluorescent microscopy". Why expression was not analysed also by western Blot same as for MCA2?
Please see major comment #64 for a detailed answer.

24) Line 204: I could not find any mention of MyoF (Pf3D7_1329100) in reference 65. Please remove reference 65 if not correct. Also reference 66 looks at Plasmodium chabaudii transcriptomes so I would specify that "This expression pattern is in agreement with the transcriptional profile of its Plasmodium chabaudii orthologue"
Reference 65 (Wichers *et al.*, 2019) provides an RNAseq transcriptome dataset for asexual blood stage development of 3D7 (originating from the same source as the 3D7 used in this study). While Ref 66 (Subudhi *et al.*, 2020) indeed contain transcriptomic data from *P. chabaudi*, the authors also provide a nice 2h window RNAseq transcriptome dataset for asexual blood stage development of *Plasmodium falciparum*. Both datasets are therefore suitable as reference for the statement about *myoF* transcription pattern. Both datasets are also easily accessible and show the pattern in a graph in PlasmoDB.

25) Line 208: Please indicate a reference for P40 being a marker of the food vacuole
Done.

26) Line 220-224: The authors should consider changing to " Taken together these results show that MyoF is in foci that are mainly close to K13 and, at times, overlapping, indicating that MyoF is found in a regular close spatial association with the K13 compartment."

The suggested wording introduces "mainly" for "frequently" and likely was in part motivated by the discrepancy in location between cell lines that we hope we now could clarify to be only minor (see major point #23). We therefore think the original wording appropriately summarises the findings (line 178): "*Taken together these results show that MyoF is in foci that are frequently close or overlapping with K13, indicating that MyoF is found in a regular close spatial association with the K13 compartment and at times overlaps with that compartment.*"

27) Line 255: In Figure 2H, and subsequent figures showing bloated FV assay, I would delineate the food vacuole with dashed line as in Birnbaum et al. 2020 to help the reader understanding where the food vacuole is.
In contrast to the Figures in Birnbaum et al. 2020, we here included the DHE staining (parasite cytosol) in images of bloated FV assays which visualizes the FV. We therefore decided to avoid any further marking, to keep the image as unprocessed as possible (see also major point 48).

28) Line 265-266: Here the title says that KIC11 is a K13 compartment associated protein, but the title of Figure 3 says KIC11 is a K13 compartment protein. I noticed that you make the difference between K13 compartment protein et K13 compartment associated protein for MyoF for example which is not clearly associated with the K13 compartment. Which one is it for KIC11?
The interpretation of the reviewer is correct, we indeed graded this subconsciously based on level of overlap. Based on the newly added quantification shown in Figure 2C, we describe KIC11 now as K13 compartment protein.

29) Line 309-310: indicate a reference for your statement "which is in contrast to previously characterised essential K13 compartment proteins".
Done, we now included Birnbaum et al. 2020 as reference for this.

30) Line 377: Figure 4I, please correct 1st panel Y axis legend
Done.

31) Line 404: replace "dispensability" with dispensable
Done.

32) Line 416: can the authors provide any speculation as to why they observed these proteins as hits in the BioID experiments?
As some of these proteins were less well or less consistently enriched, they could be background of the experiment. Alternatively, some could be proteins that only transiently interact with the K13 compartment.

33) Line 451: Where the "97% of proteins containing these domains also contain an Adaptin_N domain and function in vesicle adaptor complexes as subunit α" come from. Do you have a reference?
The statement now includes references and reads (with small changes to original submission): *"More than 97% of proteins containing these domains also contain an Adaptin_N (IPR002553) domain (Blum et al., 2021) and in this combination typically function in vesicle adaptor complexes as subunit α (Hirst and Robinson, 1998; Traub et al., 1999) (Figure 5D) but no such domain was detectable in KIC5."*

34) Line 465-467: the same could be said for KIC4 as it also has a VHS domain.

The critical issue is the combination of domains and their position within the protein. While KIC4 also contains a VHS domain, the VHS domain in KIC4 is N-terminal, not in a central position and it is also not the first structural domain to be identified in KIC4. The similarity to adaptin domains was already described ((Birnbaum *et al.*, 2020) and annotated in PlasmoDB) and these domains are also involved in vesicle formation and trafficking. These aspects of the statement can therefore not be extended to KIC4. With regards to VHS domains being involved in vesicle trafficking, this is already stated in line 538: *«KIC4 contained an N-terminal VHS domain (IPR002014), followed by a GAT domain (IPR004152) and an Ig-like clathrin adaptor α/β/γ adaptin appendage domain (IPR008152) (Figure 5A-C, Figure S8). This is an arrangement typical for GGAs (Golgi-localised gamma ear-containing Arf-binding proteins) which are vesicle adaptors first found to function at the trans-Golgi (Dell'Angelica et al., 2000; Hirst et al., 2000).»*

35) Line 477-479: Can be rephrased to "However, we found this protein as being likely dispensable for intra-erythrocytic parasite development and no colocalisation with K13 could be demonstrated, suggesting a limited role for PF3D7_1365800 in endocytosis. Or something like that. Makes it clearer.

We rephrased this sentence and it now reads (line 592): *"However, we found this protein as being likely dispensable for intra-erythrocytic parasite development and no colocalisation with K13 was observed, suggesting PF3D7_1365800 is not needed for endocytosis".*

36) Line 535: Have AP-2α or AP-2β been shown to be at the K13 compartment?

AP2μ is at the K13 compartment (Birnbaum *et al.*, 2020). Adaptor complexes are heterotetramers and their subunits do not typically function on their own and this is conserved across evolutionarily distant organisms. In agreement that this is also the case in *P. falciparum*, Henrici et al. (Henrici *et al.*, 2020a) showed that both, AP-2α and AP-2β, were present in an AP2*μ* Co-IP, indicating that the AP2 complex consist of the 'classical' subunits in *P. falciparum*. Therefore, the presence of all subunits at the K13 compartment is very likely, although this has only been experimentally confirmed for AP2*μ*. Of note, for *Toxoplasma gondii* the presence of AP-2α and AP-2β at the micropore has been experimentally confirmed (Wan *et al.*, 2023; Koreny *et al.*, 2023) and interaction suggested by presence in the same IP as DRPC (Heredero-Bermejo *et al.*, 2019).

37) Line 569: reference 43 is wrong

We thanks the reviewer for pointing this out – we removed Ref 43.

38) Line 746: typo "ot" instead of or.

Changed.

39) Line 801: method for Domain Identification using AlphaFold specify that RMSDs of under 5Å over more than 60 amino acids are listed in the results. However, there is a typo in Figure 5B for KIC5 where it says "RMSD 4.0 Å over 8 aa". Please correct.

Done. In addition, we have now applied a more stringent cut-off of 4Å over more than 60 amino acids to ensure a higher reliability of our hits. This decision was based on results from our preprint (Behrens and Spielmann, 2023). Because of this the phosphatase domain in KIC12 is no longer included in this manuscript and accordingly the following sentence has been deleted. *"In KIC12 we identified a potential purple acid phosphatase (PAP) domain. However,*

with the high RMSD of 4.9 Å, the domain might also be a divergent similar fold, such as a C2 domain, which targets proteins to membranes."

40) Line 856: In Figure 1E, please use the same Y axis legend as in Figure 2D "relative growth at day 4 [%] compared with 3D7"
Done.

41) Figure S1: Some PCR gels check for integration are presented as 5', 3' and ori whereas other gels are presented as ori, 5' and 3'. This is confusing.
We agree that ideally the order of sample loading should be consistent and we apologise for this. The explanation for this is that these gels were run by different people at different times before we were able to better standardize the loading scheme. However, in the interest of not unnecessarily using resources for something that has a similar meaning, we would prefer not to repeat these PCRs and re-run them only for consistency reasons (as the conclusion is not affected by the different loading schemes).

42) Figure S1: Why was the expression of only MCA2 was verified by Western blot? What about the other proteins?
See response to major comment 56.

43) Line 493: Considering KIC11 was not involved in HCCU or ART resistance it might be worth mentioning in this section that it is of note that there are no domains detected that would be involved in endocytosis.
We agree that this is the case, however it is also the case for all other proteins that either are not involved in endocytosis and/or lowered susceptibility to ART. We therefore now added a summary statement addressing this in line 602: *"In contrast, the K13 compartment proteins where no role in ART resistance (based on RSA) or endocytosis was detected, KIC1, KIC2, KIC6, KIC8, KIC9 and KIC11, do not contain such domains (Figure 5E)."* We did not add this at the suggested part of the manuscript as at that point the domain search results are not yet introduced and doing this each time for all the individual proteins would disconnect the flow of the manuscript.

44) Line 503-506: is it wise to generate more drugs that target a pathway that is already highly susceptible to mutations? The authors should add a statement explaining how this might be avoided.
The only protein for which mutations do not have a large fitness cost is K13 (see also our preprint on fitness cost of *ubp1* mutation (Behrens *et al.*, 2023) and even with K13 the level of resistance seems to be limited by amino acid deprivation when endocytosis is reduced (Mesén-Ramírez *et al.*, 2021). We therefore do not think that this pathway is particularly prone for mutations. Further, the number of commercial drugs targeting the "endproduct" of endocytosis (hemoglobin digestion and detoxification of heme) highlight it as the most prominent vulnerability for drug-based intervention if we go by number of commercially available drugs acting on things associated with a single process.

45) Throughout, scale bars are stated in the figure legends at the end of the legend. This is a slightly confusing format. The authors should consider stating the scale bar for each sub-legend where a fluorescence image is taken.

Done.

** Referees cross-commenting**
After reading reviewer 2 and 3's comments, I think there are significant overlaps in the key points raised in terms of questions about fusion proteins and their potential partial mis-localisation, better descripton of results and target selection. Overall I think we agree that the work has potential, but in its current form does not represent a major advance. It would be immensely helpful if the manuscript would be carefully edited for a better flow and linear description of results.
We now rearranged the manuscript for better flow but would like to highlight that the many requests for smaller experimental issues (and "better description of results") worked somewhat in the opposite way of a more linear description. We hope the rearranged version acceptably balances these two issues. The issues raised in regards to target selection and potential partial mis-localisation are addressed in our responses mainly to this reviewer. Please also see comments on systems used at the end of the rebuttal.

Reviewer #1 (Significance (Required)):

The authors set out to test whether other proteins that are in the vicinity of K13 are involved in mediating ART resistance and endocytosis. This is an interesting question. However, other than MCA2 which was already known to be involved in mediating ART resistance (and was not tested for its involvement in endocytosis), none of their candidate proteins seem to be involved in mediating both these functions. The authors show that the other proteins tested appear important for parasite growth, with KIC12 and MyoF involved in mediating endocytosis. While these findings are novel, the KS approach used by the authors casts some doubt over the findings, and would mean that these findings would have to be re-tested with a more reliable approach, such as the GlmS system or generating a conditional knockout using the DiCre system. Despite not advancing our understanding of ART resistance, or identifying further players involved in this process, this manuscripts provides two candidates that are involved in mediating endocytosis and a further candidate that appears to be important for parasite growth. Further work on these proteins will be required to understand their exact roles. As stated above, there is currently limited interest for these results (limited to researchers working on endocytosis in apicomplexan parasites and possibly the wider endocytosis field from an evolutionary perspective), however with further work, this could increase the impact and interest of this work substantially.
The authors do not describe any novel methods/approaches within this work.
In the significance statement the reviewer indicates that other systems would have been more reliable for the work here. This is addressed in our response above and in a detailed considerations on the properties of conditional inactivation systems at the end of the rebuttal. The systems used in this work were not only chosen because they permit rapid targeting of many different proteins, but because they have merits that are beneficial for our assays. In fact many of the functional assays in this manuscript are difficult or impossible to carry with the suggested conditional inactivation systems (please note that we have extensive experience with the systems considered preferable:
- DiCre (Birnbaum *et al.*, 2017; Mesén-Ramírez *et al.*, 2019; Mesén-Ramírez *et al.*, 2021; Wichers *et al.*, 2022; Kimmel *et al.*, 2023)
- glmS (Wichers *et al.*, 2021c; Wichers *et al.*, 2021a; Wichers *et al.*, 2022; Wichers-Misterek *et al.*, 2023)).

Reviewer #2 (Evidence, reproducibility and clarity (Required)):
In a previous publication the Spielmann lab identified the molecular mechanism of ART resistance in P. falciparum by connecting reduced levels of the protein K13 to decreased endocytosis (uptake of hemoglobin from the RBC cytosol), which results in reduced ART susceptibility. Using quantitative BioID the authors further identified proteins belonging to a K13 compartment, highlighting an unusual endocytosis mechanism.
In the present manuscript the authors follow up on this work and closely examine ten more proteins of the K13/Eps15-related "proxiome". They successfully link MCA2 to ART resistance in vitro, while the proteins MyoF and KIC12 are involved in endocytosis but do not confer in vitro ART resistance when impaired. They further characterize one candidate (KIC11) that partially colocalizes with K13 in trophozoites but to a lesser degree in schizonts. Growth assays suggest an important function for KIC11 in late stages of the intraerythrocytic developmental cycle. Five analyzed proteins however do not colocalize with the K13 compartment, while a sixth was refractory to endogenous tagging.
Using AlphaFold predictions of the KIC protein structures the author identify domains in most constituents of the K13 compartment, highlighting vesicle trafficking-related features that were not identified on primary sequence level before.
The combination of functional data together with structure predictions leads them to propose a refinement of the K13 compartment as being divided into proteins participating in endocytosis and proteins that have an unknown function.
We thank the reviewer for the assessment of the manuscript and the constructive comments.

Major comments:

1) -Table 1 is missing
We apologise for this mistake; Table 1 is now included.

2) -Lines 117-123: Given the total list of uncharacterized candidates encompasses 13 proteins, can the author gives the reason why only the top 10 and not all 13 were characterized in this study?
A similar point has been raised by Reviewer 1 in major comment #12, please see our response there for an explanation why we chose which targets.

3) -Line 174: 20% of observed MCA2 foci show no overlap with K13 and 21% only partially overlap, can the author confirm that the observed MCA2 foci in schizonts are the ones that co-localize with K13. (Addition of a schizont stage image in Fig 1C would be sufficient).
We now extended Figure 4C with images of MCA2-Y1344^{STOP}-GFP+mCherryK13 parasites covering the schizont and merozoite stage, showing that the majority of the MCA2 foci in schizonts are also mCherry-K13 positive.

4) -The localization and observed phenotype of KIC11 is interesting but unfortunately the authors do not explore it further. Does KIC11 localize with markers of e.g. the secretory organelles (micronemes or rhoptries) in schizonts and could therefore be involved in RBC invasion?
While we intended to focus mainly on the endocytosis aspect of these proteins, we see the reviewer's point and now generated new cell lines enabling assessment of spatial association of KIC11 with markers for rhoptry (ARO), micronemes (AMA1), and inner membrane complex

(IMC1c). This revealed that the KIC11-GFP signal in schizonts does not overlap with apical organelle markers and the signal does not resemble a typical apical localization. In addition, we assessed all three organelle markers after inactivating KIC11 by knock sideways which showed that KIC11 inactivation has no apparent effect on the appearance of these markers, suggesting no major alterations in schizont morphology in respect to apical markers. These results are now presented as Figure S3A and in line 304 of the results.

5) Can the author distinguish if KIC11 is involved in RBC invasion or in establishment of the ring-stage parasite?
In order to look into this, we performed egress/invasion assays, quantifying schizont and ring stage parasites in tightly synchronized parasites at two different time points (pre-egress: 38-42 hpi & post-egress: 46-50 hpi). This revealed a significant decrease in newly formed ring stage parasite per ruptured schizont in parasites with inactivated KIC11, while the egress efficacy remained unaffected. This indicated an invasion or very early ring stage development defect (new Figure 2H, Figure S3G). To further determine at which point exactly the phenotype occurs (ie during invasion or early after invasion) would require extensive experimentation that goes beyond the scope of this study (e.g. invasion assays using video microscopy with a representative number of parasites or sophisticated flow based quantification assays). We hope by excluding egress and gross changes of apical organelles as well as no indication for similar number of early rings (indicating it is invasion or a very early ring-establishment phenotype) will sufficiently narrow down the phenotype for labs interested in invasion to more definitely answer this question.

Minor comments:

1) Table S1: Please add the criterion for the order of proteins (abundance in "proxiome"?) in the table as a separate column. I would also suggest adding a new column that highlights the 10 proteins investigated in this study as I found the color-coding slightly confusing.
Done as suggested: we now include the “average log2 Ratio normalized Kelch13” values from the four DiQ-BioID experiments performed with K13 in (Birnbaum *et al.*, 2020), as well as the suggested column to highlight the investigated proteins. Please also see reviewer 1 major point # 12 for additional information on the selection criteria and how this was added to the manuscript.

2) -154-155: There is a discrepancy between the text and Fig1C regarding the % of partial overlapping and non-overlapping foci.
We thank the reviewer for pointing this out, this was corrected.

3) -The y-axis label is missing in Fig 3E
Done.

4) -Fig 4I left graph, the superscript 2 is missing in µm2
We thank the reviewer for pointing this out, this is now changed.

5) -Did the author colocalize KIC11 in schizonts with other proteins found in the K13 compartment group of proteins not involved in endocytosis/ART resistance? This may help to further subgroup these proteins.

This is an interesting point but would actually be technically challenging to do. For this we would need to generate a KIC11^{endo} parasite line for each of these KICs and then do co-localisation in schizonts. However, the outcome of this likely would not be very clear. The reason for this is as follows. There are foci of KIC11 that do overlap with K13 in schizonts. One can expect that these foci show KIC11 at the K13 compartment and that the other KICs would overlap with KIC11 in these K13 foci in schizonts. Hence, we would also need to see K13 to find the non-K13 compartment KIC11 foci and see if these contained the KIC of interest. This is technically challenging because it would mean we would need a third fluorescent protein which is not that trivial to do. Due to the difficulty to do this and the large amount of work involved and the already considerable amount of data in this manuscript, we believe this will be better suited for a different study.

6) -As a general comment: to make the beautiful IFAs more accessible to a broader readership, I would encourage the authors to switch the color-coding to green/magenta/blue or an equivalent color system or add grayscale images.
This was done as suggested, all fluorescence images are now provided as greyscale images and the overlays are shown in magenta/green.

Reviewer #2 (Significance (Required)):

Characterizing the molecular components involved in Plasmodium endocytosis will not only reveal interesting biology in these highly adapted parasites, but will more importantly lead to a better understanding and potentially open new avenues for intervention of ART resistance. The here presented manuscript is a carefully executed follow-up on previous work done in Dr. Spielmann's lab focusing on the K13 compartment. The authors use established assays to characterize novel components and reveal three new players in endocytosis with one mediating ART resistance in vitro. The proposition that parts of the K13 compartment have a function other than endocytosis is interesting, but will have to await more data from future studies.
Taken together this manuscript adds significantly to our understanding of endocytosis in P. falciparum.

This work is of interest for cell and molecular biologists working on Apicomplexa, but especially for the Plasmodium community.
We thank the reviewer for this positive assessment.

I am a cell and molecular biologist working on Toxoplasma gondii

Reviewer #3 (Evidence, reproducibility and clarity (Required)):

Summary:
The authors characterized 4 proteins from P. falciparum via cellular (co-)localization, endocytosis, parasite growth, and artemisinin resistance assays. These proteins have been identified as candidates for Kelch13 compartment and a possible role in endocytosis in their previously work with quantitative BioID for potential proximity to K13 and Eps15 (Birnbaum et al. 2020). In the current work, additional 6 proteins were not confirmed as being associated to the K13 compartment. This experimental work was complemented by an in-silico analysis of protein domains based on AlphaFold algorithm. For this protein structure evaluation all

proteins were chosen, which were experimentally confirmed to be linked to the K13 compartment in the current publication and previous work. With the work 3 novel proteins linked to artemisinin resistance or endocytosis could be functionally described (KIC12, MCA2, and MyoF) and a number of hypotheses were generated.
We thank the reviewer for the assessment of the manuscript and the constructive comments.

Major comments:
The quality of the presented work is solid, the experimental design is adequate, and methods are presented clearly. The publication contains a lot of results both presented in text and in the figures and it is not always straight forward for the reader to follow the descriptions due to many details presented and a lack of context for some of these experiments.
We thank the reviewer for this overall positive assessment.

We now reordered the results section in an attempt to increase the flow of the manuscript. We also made changes to improve the context for the results. Given the further (very valid) requests for data on schizonts and invasion, there was an increased danger for a less linear manuscript that we hope to have acceptably managed with the re-arrange.

Specific suggestions for consideration by the authors to improve the manuscript.
Abstract:
1) R 31: Mention how the 4 proteins were identified as candidates, you need to refer to previous work to clarify this
To clarify this the sentence was changed to (line 31): *"Here we further defined the composition of the K13 compartment by analysing more hits from a previous BioID, showing that MyoF and MCA2 as well as Kelch13 interaction candidate (KIC) 11 and 12 are found at this site."*

2) R38: "Second group of proteins" is confusing - different from the 4 mentioned above? Significance to endocytosis unclear. Please unify terminology in the manuscript, see also comment below on proxiome.
We changed the wording to clarify the group issue in the abstract as follows line 34: *"Functional analyses, tests for ART susceptibility as well as comparisons of structural similarities using AlphaFold2 predictions of these and previously identified proteins showed that canonical vesicle trafficking and endocytosis domains were frequent in proteins involved in resistance or endocytosis (or both), comprising one group of K13 compartment proteins, While this strengthened the link of the K13 compartment to endocytosis, many proteins of this group showed unusual domain combinations and large parasite-specific regions, indicating a high level of taxon-specific adaptation of this process. Another group of K13 compartment proteins did not influence endocytosis or ART susceptibility and lacked detectable vesicle trafficking domains. We here identified the first protein of this group that is important for asexual blood stage development and showed that it likely is involved in invasion."*

3) Abstract can only be understood after reading the full publication
We attempted to amend this by expanding the abstract, particularly the changes highlighted in the previous two points.

Results:
4) Table 1 is missing from the submitted materials

We apologise for this mistake. Table 1 is now included.

5) Consider to shorten and stratify the result section to focus on the significant data
We rearranged the results in an attempt to streamline this section and are now starting with MyoF in the revised manuscript. However, as highlighted by the requests from reviewer 1, many details need to be available to support our conclusions. For instance the fact that GFP-tagging partially inactivated MyoF asked for further data to support our conclusion (HA-tagged version, showing that the location of the GFP-tagged version was consistent with the HA-tagged version, showing to what extent the different constructs affected growth and correlated with number of vesicles and bloating, see new figure 1M) or that KIC12 has two locations. Overall, we are therefore hesitant to remove data or description from the result part.

6) Unclear how the localization and functionalization assays might be impaired by the fusion proteins
Significance of ART resistance assay is not clear, in presence of strong growth effects due to inactivation or truncation of genes/proteins
As indicated also in the example given in the previous point (this reviewer #5), the use of different cell lines (GFP-tagged live cells and small epitope tag in IFA) for targets with an indication for an effect of the tagging confirm that the location we assigned is reasonable. In the case of MyoF, the HA-tagged line, the partial inactivation due to GFP and the further inactivation in the GFP-tagged line by knock sideways show plausible increase of phenotypes (vesicle accumulation and bloated FV assays). Thereby the GFP-tagged line can be seen as a partial inactivation line that further supports our conclusions and overall this paints a consistent picture of the function of this protein in endocytosis (see new Figure 1M better illustrating this). Please note that the difference in location shown by this line compared to the HA-tagged proteins is only small (see also reviewer 1 major point 23ff). See also general discussion on tags at the end of this rebuttal.

Significance of ART resistance assay: The ‘ART resistance assay’ is done comparing +/- ART (DHA) in identical parasites (originating from the same culture and the same condition). Hence, any growth effects are cancelled out and effects in reducing ART susceptibility would - if at all - be underestimated (see more detailed response to point 28, reviewer 1 and controls in Birnbaum et al., 2020 where we tested an unrelated essential protein, unrelated chemical insult and rapalog on 3D7 and did not detect any effect on RSA survival).

MCA
7) Stratify results, order by significance of findings, it appears to be described in chronological order, improve readability/flow, eg ART resistance if mentioned in r138, but only reported in r183ff
We attempted to stratify, but then the reason for generating the partial MCA2 disruption parasite line becomes very arbitrary and would leave the reader wondering why we at all truncated the protein at two thirds of the protein. Hence, we do not see a way around this chronological reporting. However, this part is now not at the start of the experimental results section anymore, possibly making it overall a bit more palatable.

MyoF
8) R195 to 197 - consider moving to discussion as it is distracting here

This was shortened and additional information (asked for by reviewer 1, major point 22) to clarify that MyoF was previously called MyoC, was added (line 147): *"The presence of MyosinF (MyoF; PF3D7_1329100 previously also MyoC), in the K13 proxiome could indicate an involvement of actin/myosin in endocytosis in malaria parasites. "*

9) Term proxiome is introduced above, but not used in result section - suggest to unify language, eg r195 uses "K13 compartment DiQ-BioIDs" instead, which is not very convenient for the reader
We carefully reviewed this and made this more consistent.

10) What is the enrichment factor? Please provide for this and the following proteins, eg in Table 1
The enrichment factor is log2 enrichment over control and this is now provided in table S1 (see also detailed answer for Reviewer 1 major point 12).

11) R225 to 243 - overall significance of the growth experiments with mislocaliser is not clear, consider removing from manuscript or explain relevance more clearly
See also point 28, reviewer 1: This experiment is actually quite important. It shows that if we conditionally inactivate the GFP-tagged MyoF, the growth is further reduced, as stated in line 208. It might have been confusing that the mislocalisation is only partial, but this is equivalent to a partial knock down and hence is useful. This becomes even more relevant with the specific assays following in the next paragraph: while the tagging of MyoF already resulted in vesicles, conditional inactivation with KS generated even more vesicles, showing that the same phenotype was rapidly increased when MyoF was further inactivated by a different means and this also correlated with growth. Hence, this is actually a very consistent phenotype that despite some shortcomings of the tools available to analyse this protein (due to the partial inactivation by the GFP tag) in our eyes looks very convincing. We now added a graph showing the correlation of growth and phenotypes to illustrate this (Figure 1L).
We also tried to make this clearer by changing line 200 to: *"Hence, conditional inactivation of MyoF further reduced growth despite the fact that the tag on MyoF already led to a substantial growth defect, indicating an important role for MyoF during asexual blood stage development."*
And line 208 to:" *This was even more pronounced upon conditional inactivation of MyoF by KS (Figure 1H), suggesting this is due to a reduced function of MyoF."*

12) KIC11/KIC12
Enrichment factor?
The enrichment ('average log2 Ratio normalized Kelch13 from Birnbaum et al. 2020') is 1.65 for KIC11 and 1.32 for KIC12, which is now also explicitly shown in column D of Table S1.

** Referees cross-commenting**

I would like to applaud reviewer #1 for a great, very thorough review and lots of detailed suggestions.
I agree with the conclusions mentioned in the significance evaluation from reviewer #1 and #2: the work presented does not contain novel methods and the scope is rather narrow with the current results. (I am working on clinical studies with novel antimalarial agents)

Reviewer #3 (Significance (Required)):

On the one hand side, the authors have wrapped up some of the remaining protein candidates of the K13 compartment and could verify 4 of 10 proteins. The work is of interest for the scientific community working on endocytosis and malaria drug resistance mechanisms. Overall, the conclusions and findings from the previous work, Birnbaum et al. 2020, could be confirmed and extended mainly using the methods previously described. On the other hand, the authors made use of progress in protein structure predictions and identified domains linking the K13 compartment proteins to putative functions. The overlaid protein folds of the newly identified domains in figure 5 look convincing, but I can't comment on the technical details or cut-off used for this in-silico analysis.

Extended general remarks on the systems used for this work:
Mainly reviewer 1 suggest (in the general comments and the significance statement) that other systems would have been better suited to use for this work, namely glmS and diCre and also has concerns about the large tag which is seconded by a comment of reviewer 3. In light of this we here provide some extended considerations on the properties for conditional systems and tagging in regards to the goals of this work.

We would like to point out that we do have experience with the systems considered better-suited by the reviewer (one of the first authors has extensively used glmS (Wichers *et al.*, 2021c; Wichers *et al.*, 2021a; Wichers *et al.*, 2022; Wichers-Misterek *et al.*, 2023) and our lab was one of the first to adopt the diCre system in *P. falciparum* parasites and we regularly us it (Birnbaum *et al.*, 2017; Mesén-Ramírez *et al.*, 2019; Kimmel *et al.*, 2023)). Clearly, these methods have a lot of strengths but there are a number of issues to be considered for the assays we use in this work (see the next section on conditional inactivation systems). In a nutshell, we believe diCre would give a more reliable readout of the absolute level of "essentiality" (i.e. importance for growth) but is unsuitable or at least difficult to use for the assays that reveal the function of our interest in this work. GlmS basically combines the drawbacks of diCre and knock sideways and hence for most targets is not expected to give a better readout of level of "essentiality" but is similarly difficult to use for our specific assays. The fact that both of these systems are possible to use without adding a tag to the target may be an advantage but without tag one loses some very important features that can be critical to understand the outcome with a given system (see considerations on the tag further below).

<u>Conditional inactivation systems:</u>
1. **speed of inactivation:** glms acts on mRNA and diCre on the gene level, which makes them slower than techniques acting directly on the protein such as DD or KS. With diCre, mRNA and protein is still left, even if the gene is very rapidly excised. For instance for Kelch13 it takes 3-4 days after excising the gene until protein levels have waned enough that this manifests in a reduced growth (Birnbaum *et al.*, 2017). While in some instances diCre permits same cycle analyses if the protein has a very rapid turn-over (e.g. Rab5a, (Birnbaum *et al.*, 2017)), control in a few hours is still difficult. For vesicle accumulation and bloated food vacuole assays, which are done over comparably short time frames and with specific stages, it is rather challenging to hit the correct time of induction to have all the cells at the correct stage with suitably (and uniformly, ie all cells) sufficiently reduced target protein levels during the assay time.

Slow acting systems are also more prone to secondary effects. The more immediate the inactivation, the closer it is to the core of the affected function. With vesicle trafficking processes this is particularly relevant as all vesicle trafficking in a cell is interconnected and there are always recycling pathways that maintain the membrane and protein homeostasis of individual compartments. Particularly for endocytosis there seem to be compensatory capacities at least in other organisms (see e.g. (Chen and Schmid, 2020)). One reason why knock sideways was developed is that it permitted to avoid compensatory changes when vesicle adaptors are inactivated (Robinson *et al.*, 2010).

The comparably short time frame for malaria parasites to go through different stages during blood stage development also is an issue relevant for inactivation speed. The advantage of speed and the danger of obscured phenotypes is highlighted by our work on VPS45 which showed that in trophozoites this protein is involved in the transport of hemoglobin to the FV whereas in late stages it also has a role in secretory processes. Both of these functions we were able to specifically assess in the same growth cycle using KS to rapidly inactivate the protein (Bisio *et al.*, 2020) but with a slower system would have been more complicated to dissect.

Speed of effect with glmS: unless the KS does not work well, glmS is slower acting than KS (it does not target the already synthesised protein which can remain in the cell) and also often suffers from only partial inactivation, hence the benefit of using it here is unclear. The option to have an untagged protein is a plus, however it also is a minus, as assessing efficiency (particularly in live cells e.g. for bloated assays etc a fluorescent tag is the only direct option to assess inactivation of target) is critical to ensure the phenotype manifests at the stage of interest.

2. **lethality/absolute phenotypic effects** are detrimental to some assays to study the functions we are interested in for this work: no RSA can be conducted, if the gene is lost and the parasites die. Again, with diCre, one could attempt to hit the point when the parasites have lost sufficient amounts of the target protein when they are placed under ART but then the parasites need to continue growing for ~3 days, which is not possible if the cKO is lethal except for very slowly turning over proteins. However, in that latter case, the parasites likely still had full functionality of the target protein at the beginning of the RSA, when the drug pulse happens and there would be no effect. Knock sideways solves these problems by permitting knock sideways inactivation only under ART (or with a few hours pre-incubation depending on the inactivation speed) to not yet affect growth in a severe manner but inhibiting the process the protein is involved in. It may be possible to use glmS for RSAs, but the slow speed would complicate it (it would not permit control of target protein levels in a matter of a few hours to inactivate the target protein and then re-install it).

None-absolute inactivation is also a strength for some functional assays. While we really like using diCre, in the case of EXP1 it made it necessary to complement the *exp1* cKO parasites with low levels of EXP1 to be able to do functional assays without killing the parasites (Mesén-Ramírez *et al.*, 2019; Mesén-Ramírez *et al.*, 2021). While the lethality issue does not apply to glmS (like knock sideways, it also can be tuned), it is unclear what would be gained over knock sideways. Knockdown levels with glmS vary from gene to gene and cannot be predicted, it is in most cases considerably slower than KS, it requires glucosamine which becomes toxic at

higher concentrations and might introduce off target effects and tracking protein levels during the assay would equally need GFP tagging.

Integration of properties of conditional systems

Given the above discussed properties, several factors have to be considered to be able to use a system for a given assay. Stage-specific transcription is one example. For diCre a protein not expressed in e.g. rings permits to remove the gene and the protein is never made in that parasite development cycle. We exploited this for instance for two proteins only expressed from the trophozoite stage onwards (Kimmel *et al.*, 2023). However, if lethal (absolute effect problem), this also means one can also only see the phenotype on onset of expression of the target (e.g. if in mitosis, the first nuclear division in case the protein is absolutely essential for the process). This is just one example of such issues. Expression timing, turnover of the protein and homogeneity of stage-specific loss of protein will all influence how clearly the phenotype can be determined. All this will decide the exact time of loss/inactivation of the target protein to levels generating a phenotype and ideally therefore can be monitored during an assay (see considerations on tagging).

For these reasons vesicle accumulation or bloated food vacuole assays are difficult with slow systems as ideally the target should rapidly be inactivated at the trophozoite stage and the result monitored before the cells have moved to the schizont stage. For this a well responding knock sideways is ideal as the protein can be rapidly taken away (sometimes within seconds) to visualise the immediate, direct effect in the cell.
As shown for KIC11, there is also no disadvantage of using KS for proteins with other assays or proteins that result in different phenotypes. It permits stage-specific same cycle inactivation without having to worry about the turnover of mRNA and protein (Fig. 2F,G). Thus, besides the advantages of knock sideways for endocytosis related assays and RSAs, we also see no disadvantage of using knock sideways for the functional study of KIC11 which has a role other than endocytosis. KS also permits to specifically target the K13 pool of KIC12, something impossible or very difficult to do with other systems. Hence, we are of the opinion that the system for inactivation was adequate for most of the proteins analysed in this manuscript.

Large tag: we agree that GFP-tagging can be a disadvantage but in our opinion its benefits often outweigh the drawbacks because it permits easy and immediate (on individual cell level, if need be) monitoring of the presence/location of the target protein (e.g. after KS, but given the discrepancy of the timing between gene excision and protein loss, it might be even more important for techniques such as diCre). No fixing/permeabilisation (prone to artifacts, prevents immediate view of cells) to detect a target with specific antibodies or via a small tag is needed with GFP. Similarly, the use of Western blots to do this is time consuming and impractical if monitoring of left-over protein in the course of an assay such as a bloated food vacuole assay is needed.

In many cases, adding GFP has no negative effect. In addition, if the bulky folded structure of GFP is tolerated, it usually also tolerates the 2 to 4 12kDa FKBP domains in our standard tag. We also typically add a linker. This approach has worked for a large number of different proteins, including many essential ones for which we would not otherwise have obtained the integration cell lines (Birnbaum *et al.*, 2017; Jonscher *et al.*, 2019; Hoeijmakers *et al.*, 2019; Birnbaum *et al.*, 2020; Kimmel *et al.*, 2023; Sabitzki *et al.*, 2023). Hence, whenever a cell line is obtained with it, this tag in most cases is not a disadvantage. Admittedly an exception in this

is MyoF and to some extent maybe MCA2 (we would like to stress that in the case of MCA2 the reason for not being able to obtain the full length tagged cell line is unclear: the protein can be severely truncated to less than 3% of its amino acid sequence and a GFP-tag is tolerated on the version with 2/3s of the protein left, which gives no good reason why the full length was not obtained; a potential reason could be a dominant negative effect). However, we obtained the full length with a small tag detected by IFA for both, MyoF and MCA2 and the location of these agreed well with the GFP tagged versions, indicating that the GFP-tagged versions are useful to show the location of these proteins in live cells.

There are also tricks to attempt monitoring the effect of e.g. diCre without tagging the target. For instance, if a fluorescent protein is connected to excision without actually being fused to the target (ie excision of the gene leads to its expression of e.g. GFP), which would avoid adding a tag to the target itself. However, the problem with this is that expression of GFP does only show excision, but mRNA producing the target protein and left over target protein may still be there in the cell. All in all, the GFP-tag on the target, while with some drawbacks, is still our preferred method to control to monitor the target protein in the cell (in principle permitting quantification of ablation efficiency on the individual cell level).

Conclusion on these considerations for this manuscript

Based on these considerations we do not see the immediate benefit of changing the system for the conclusions drawn from this study and are unsure if they are indeed better suited for this work as suggested. While a more exact readout of "essentiality" might be possible with the diCre system we are of the opinion this is less important than learning the function of a protein which - as outlined above - we believe to be considerably more difficult with diCre and even more so with glmS considering our target functions. The same applies to target specific cellular pools of a protein as done here for KIC12. Clearly MyoF is one example where the employed systems shows limitations, but with the new Figure part showing consistency in phenotype with degree of inactivation (importantly with two different forms of inactivation) and the clarification that the location of the GFP-tagged and HA-tagged versions are actually quite similar in location, we do not think employing an extra system is warranted for the conclusions of this work. Admittedly, the apparent lack of need in ring stags might give an opening to attack MyoF using diCre (by excision before its major expression peak), but depending on lethality this might preclude extended analyses (possibly vesicle assays, for sure not RSAs).

In the end the question is, if our approach provides the function of target analysed in this work and based on the data in our manuscript and the arguments in the rebuttal, we are reasonably confident that this is the case. It is not very likely the other mentioned techniques would result in a different conclusion on the function of the here studied proteins. In fact, we expect other commonly used techniques to be less suitable for the key assays in this work.

References used in our responses to the reviewers' comments:

Behrens, H.M., Schmidt, S., Peigney, D., Sabitzki, R., Henshall, I., May, J., *et al.* (2023) Impact of different mutations on Kelch13 protein levels, ART resistance and fitness cost in Plasmodium falciparum parasites. *bioRxiv* 2022.05.13.491767.

Behrens, H.M., Schmidt, S., and Spielmann, T. (2021) The newly discovered role of endocytosis in artemisinin resistance. *Med Res Rev* med.21848.

Behrens, H.M., and Spielmann, T. (2023) Identification of domains in Plasmodium falciparum proteins of unknown function using DALI search on Alphafold predictions. *bioRxiv*

2023.06.05.543710.
Birnbaum, J., Flemming, S., Reichard, N., Soares, A.B., Mesén-Ramírez, P., Jonscher, E., *et al.* (2017) A genetic system to study Plasmodium falciparum protein function. *Nat Methods* **14**: 450–456.
Birnbaum, J., Scharf, S., Schmidt, S., Jonscher, E., Hoeijmakers, W.A.M., Flemming, S., *et al.* (2020) A Kelch13-defined endocytosis pathway mediates artemisinin resistance in malaria parasites. *Science (80-)* **367**: 51–59.
Bisio, H., Chaabene, R. Ben, Sabitzki, R., Maco, B., Baptiste Marq, J., Gilberger, T.W., *et al.* (2020) The zip code of vesicle trafficking in apicomplexa: Sec1/munc18 and snare proteins. *MBio* **11**: 1–21.
Blum, M., Chang, H.Y., Chuguransky, S., Grego, T., Kandasaamy, S., Mitchell, A., *et al.* (2021) The InterPro protein families and domains database: 20 years on. *Nucleic Acids Res* **49**: D344–D354.
Borrmann, S., Straimer, J., Mwai, L., Abdi, A., Rippert, A., Okombo, J., *et al.* (2013) Genome-wide screen identifies new candidate genes associated with artemisinin susceptibility in Plasmodium falciparum in Kenya. *Sci Rep* **3**.
Bozdech, Z., Llinás, M., Pulliam, B.L., Wong, E.D., Zhu, J., and DeRisi, J.L. (2003) The transcriptome of the intraerythrocytic developmental cycle of Plasmodium falciparum. *PLoS Biol* **1**: e5.
Burnette, W.N. (1981) "Western Blotting": Electrophoretic transfer of proteins from sodium dodecyl sulfate-polyacrylamide gels to unmodified nitrocellulose and radiographic detection with antibody and radioiodinated protein A. *Anal Biochem* **112**: 195–203.
Casella, J.F., Flanagan, M.D., and Lin, S. (1981) Cytochalasin D inhibits actin polymerization and induces depolymerization of actin filaments formed during platelet shape change. *Nature* **293**: 302–305.
Cerqueira, G.C., Cheeseman, I.H., Schaffner, S.F., Nair, S., McDew-White, M., Phyo, A.P., *et al.* (2017) Longitudinal genomic surveillance of Plasmodium falciparum malaria parasites reveals complex genomic architecture of emerging artemisinin resistance. *Genome Biol* **18**: 78.
Chen, Z., and Schmid, S.L. (2020) Evolving models for assembling and shaping clathrin-coated pits. *J Cell Biol* **219**.
Dell'Angelica, E.C., Puertollano, R., Mullins, C., Aguilar, R.C., Vargas, J.D., Hartnell, L.M., and Bonifacino, J.S. (2000) GGAs: A family of ADP ribosylation factor-binding proteins related to adaptors and associated with the Golgi complex. *J Cell Biol* **149**: 81–93.
Demas, A.R., Sharma, A.I., Wong, W., Early, A.M., Redmond, S., Bopp, S., *et al.* (2018) Mutations in Plasmodium falciparum actin-binding protein coronin confer reduced artemisinin susceptibility. *Proc Natl Acad Sci* 201812317.
Henrici, R.C., Edwards, R.L., Zoltner, M., Schalkwyk, D.A. van, Hart, M.N., Mohring, F., *et al.* (2020a) The plasmodium falciparum artemisinin susceptibility-associated ap-2 adaptin μ subunit is clathrin independent and essential for schizont maturation. *MBio* **11**.
Henrici, R.C., Schalkwyk, D.A. van, and Sutherland, C.J. (2020b) Modification of pfap2μ and pfubp1 Markedly Reduces Ring-Stage Susceptibility of Plasmodium falciparum to Artemisinin in Vitro. *Antimicrob Agents Chemother* **64**.
Henriques, G., Hallett, R.L., Beshir, K.B., Gadalla, N.B., Johnson, R.E., Burrow, R., *et al.* (2014) Directional selection at the pfmdr1, pfcrt, pfubp1, and pfap2mu loci of Plasmodium falciparum in Kenyan children treated with ACT. *J Infect Dis* **210**: 2001–2008.
Heredero-Bermejo, I., Varberg, J.M., Charvat, R., Jacobs, K., Garbuz, T., Sullivan, W.J., and Arrizabalaga, G. (2019) TgDrpC, an atypical dynamin-related protein in *Toxoplasma gondii,* is associated with vesicular transport factors and parasite division. *Mol Microbiol* **111**: 46–64.
Hirst, J., Lui, W.W.Y., Bright, N.A., Totty, N., Seaman, M.N.J., and Robinson, M.S. (2000) A family of proteins with γ-adaptin and VHS domains that facilitate trafficking between the trans-golgi network and the vacuole/lysosome. *J Cell Biol* **149**: 67–79.

Hirst, J., and Robinson, M.S. (1998) Clathrin and adaptors. *Biochim Biophys Acta - Mol Cell Res* **1404**: 173–193.
Hoeijmakers, W.A.M., Miao, J., Schmidt, S., Toenhake, C.G., Shrestha, S., Venhuizen, J., *et al.* (2019) Epigenetic reader complexes of the human malaria parasite, Plasmodium falciparum. *Nucleic Acids Res* **47**: 11574–11588.
Jonscher, E., Flemming, S., Schmitt, M., Sabitzki, R., Reichard, N., Birnbaum, J., *et al.* (2019) PfVPS45 Is Required for Host Cell Cytosol Uptake by Malaria Blood Stage Parasites. *Cell Host Microbe* **25**: 166-173.e5.
Kimmel, J., Schmitt, M., Sinner, A., Jansen, P.W.T.C., Mainye, S., Ramón-Zamorano, G., *et al.* (2023) Gene-by-gene screen of the unknown proteins encoded on Plasmodium falciparum chromosome 3. *Cell Syst* **14**: 9-23.e7.
Koreny, L., Mercado-Saavedra, B.N., Klinger, C.M., Barylyuk, K., Butterworth, S., Hirst, J., *et al.* (2023) Stable endocytic structures navigate the complex pellicle of apicomplexan parasites. *Nat Commun* **14**: 2167.
Kumari, V., Singh, A.P., Singh, J., Sharma, R., Akhter, M., Mishra, P.K., *et al.* (2018) Biochemical characterization of unusual cysteine protease of P. falciparum, metacaspase-2 (MCA-2). *Mol Biochem Parasitol* **220**: 28–41.
Lazarus, M.D., Schneider, T.G., and Taraschi, T.F. (2008) A new model for hemoglobin ingestion and transport by the human malaria parasite Plasmodium falciparum. *J Cell Sci* **121**: 1937–1949.
Lopez-Hernandez, F.J., Ortiz, M.A., Bayon, Y., and Piedrafita, F.J. (2003) Z-FA-fmk inhibits effector caspases but not initiator caspases 8 and 10, and demonstrates that novel anticancer retinoid-related molecules induce apoptosis via the intrinsic pathway. *Mol Cancer Ther* **2**: 255–263.
Lord, S.J., Velle, K.B., Mullins, R.D., and Fritz-Laylin, L.K. (2020) SuperPlots: Communicating reproducibility and variability in cell biology. *J Cell Biol* **219**.
MalariaGEN, Ahouidi, A., Ali, M., Almagro-Garcia, J., Amambua-Ngwa, A., Amaratunga, C., *et al.* (2021) An open dataset of Plasmodium falciparum genome variation in 7,000 worldwide samples. *Wellcome open Res* **6**: 42.
Marti, M., Good, R.T., Rug, M., Knuepfer, E., and Cowman, A.F. (2004) Targeting malaria virulence and remodeling proteins to the host erythrocyte. *Science* **306**: 1930–3.
Mesén-Ramírez, P., Bergmann, B., Elhabiri, M., Zhu, L., Thien, H. von, Castro-Peña, C., *et al.* (2021) The parasitophorous vacuole nutrient pore is critical for drug access in malaria parasites and modulates the fitness cost of artemisinin resistance. *Cell Host Microbe* **0**: 283.
Mesén-Ramírez, P., Bergmann, B., Tran, T.T., Garten, M., Stäcker, J., Naranjo-Prado, I., *et al.* (2019) EXP1 is critical for nutrient uptake across the parasitophorous vacuole membrane of malaria parasites. *PLoS Biol* **17**: e3000473.
Mukherjee, A., Crochetière, M.-È., Sergerie, A., Amiar, S., Thompson, L.A., Ebrahimzadeh, Z., *et al.* (2022) A Phosphoinositide-Binding Protein Acts in the Trafficking Pathway of Hemoglobin in the Malaria Parasite Plasmodium falciparum. *MBio* **13**.
Otto, T.D., Wilinski, D., Assefa, S., Keane, T.M., Sarry, L.R., Böhme, U., *et al.* (2010) New insights into the blood-stage transcriptome of Plasmodium falciparum using RNA-Seq. *Mol Microbiol* **76**: 12–24.
Robinson, M.S., Sahlender, D.A., and Foster, S.D. (2010) Rapid Inactivation of Proteins by Rapamycin-Induced Rerouting to Mitochondria. *Dev Cell* **18**: 324–331.
Sabitzki, R., Schmitt, M., Flemming, S., Jonscher, E., Hoehn, K., Froehlke, U., and Spielmann, T. (2023) Identification of a Rabenosyn-5 like protein and Rab5b in host cell cytosol uptake reveals conservation of endosomal transport in malaria parasites. *bioRxiv* 2023.04.05.535711.
Simwela, N. V., Hughes, K.R., Roberts, A.B., Rennie, M.T., Barrett, M.P., and Waters, A.P. (2020) Experimentally engineered mutations in a ubiquitin hydrolase, UBP-1, modulate in vivo susceptibility to artemisinin and chloroquine in plasmodium berghei. *Antimicrob Agents*

Chemother **64**.
Spielmann, T., Gras, S., Sabitzki, R., and Meissner, M. (2020) Endocytosis in Plasmodium and Toxoplasma Parasites. *Trends Parasitol* **36**: 520–532.
Subudhi, A.K., O'Donnell, A.J., Ramaprasad, A., Abkallo, H.M., Kaushik, A., Ansari, H.R., *et al.* (2020) Malaria parasites regulate intra-erythrocytic development duration via serpentine receptor 10 to coordinate with host rhythms. *Nat Commun* **11**.
Traub, L.M., Downs, M.A., Westrich, J.L., and Fremont, D.H. (1999) Crystal structure of the α appendage of AP-2 reveals a recruitment platform for clathrin-coat assembly. *Proc Natl Acad Sci U S A* **96**: 8907–8912.
Wagner, M.P., Formaglio, P., Gorgette, O., Dziekan, J.M., Huon, C., Berneburg, I., *et al.* (2022) Human peroxiredoxin 6 is essential for malaria parasites and provides a host-based drug target. *Cell Rep* **39**: 110923.
Wall, R.J., Zeeshan, M., Katris, N.J., Limenitakis, R., Rea, E., Stock, J., *et al.* (2019) Systematic analysis of Plasmodium myosins reveals differential expression, localisation, and function in invasive and proliferative parasite stages. *Cell Microbiol* **21**.
Wan, W., Dong, H., Lai, D.-H., Yang, J., He, K., Tang, X., *et al.* (2023) The Toxoplasma micropore mediates endocytosis for selective nutrient salvage from host cell compartments. *Nat Commun* **14**: 977.
Wichers-Misterek, J.S., Binder, A.M., Mesén-Ramírez, P., Dorner, L.P., Safavi, S., Fuchs, G., *et al.* (2023) A Microtubule-Associated Protein Is Essential for Malaria Parasite Transmission. *MBio* .
Wichers, J.S., Gelder, C. van, Fuchs, G., Ruge, J.M., Pietsch, E., Ferreira, J.L., *et al.* (2021a) Characterization of Apicomplexan Amino Acid Transporters (ApiATs) in the Malaria Parasite Plasmodium falciparum. *mSphere* **6**.
Wichers, J.S., Mesén-Ramírez, P., Fuchs, G., Yu-Strzelczyk, J., Stäcker, J., Thien, H. von, *et al.* (2022) PMRT1, a Plasmodium -Specific Parasite Plasma Membrane Transporter, Is Essential for Asexual and Sexual Blood Stage Development. *MBio* **13**.
Wichers, J.S., Scholz, J.A.M., Strauss, J., Witt, S., Lill, A., Ehnold, L.-I., *et al.* (2019) Dissecting the Gene Expression, Localization, Membrane Topology, and Function of the Plasmodium falciparum STEVOR Protein Family. *MBio* **10**: e01500-19.
Wichers, J.S., Tonkin-Hill, G., Thye, T., Krumkamp, R., Kreuels, B., Strauss, J., *et al.* (2021b) Common virulence gene expression in adult first-time infected malaria patients and severe cases. *Elife* **10**.
Wichers, J.S., Wunderlich, J., Heincke, D., Pazicky, S., Strauss, J., Schmitt, M., *et al.* (2021c) Identification of novel inner membrane complex and apical annuli proteins of the malaria parasite Plasmodium falciparum. *Cell Microbiol* **23**: e13341.
