# Supplementary material for: The Kelch13 compartment contains highly divergent vesicle trafficking proteins in malaria parasites
Source: PLoS Pathog. 2023 Dec 1;19(12):e1011814. doi: 10.1371/journal.ppat.1011814 (PMC10718435; doi:10.1371/journal.ppat.1011814)
Supplement: Attachment — Submitted filename: Response to Reviewers.pdf [file ppat.1011814.s015.pdf]

RESPONSE TO EDITOR AND REVIEWERS

Editor comment:

The revised version of the manuscript, which was originally reviewed through RevComm was reviewed by the original reviewers. As you can see in their comments two of the referees are satisfied with the significant amount of data included to address their concerns. Though, one reviewer still have major concerns primarily because they feel that some of the data regarding the characterized proteins does not support the endocytosis-ART resistance paradigm i.e. this reviewer argue that maybe not all proteins involved in endocytosis are linked with ART-resistance. I believe that these criticism should and could be addressed before making a final decision for the manuscript.

We thank the editor for giving us the chance to revise this manuscript. We now responded in detail to the concerns of this reviewer. We would like to mention that we already clearly state in the manuscript that there are K13 compartment proteins involved in endocytosis that do not influence ART susceptibility, e.g. MyoF and KIC12. Likely this is because they do not influence endocytosis in rings (MyoF expression is barely detectable in rings and Cytochalasin D has no effect, suggesting dispensability of actin/myosin for endocytosis in rings; KIC12 is not at the K13 compartment in rings). Vice versa, the opposite is the case for Kelch13 for which we showed in previous work that its inactivation does not influence endocytosis in trophozoites (based on bloated FV assays, the assay used in this work), but influences endocytosis in rings and has a strong effect on RSA survival. Hence, the ART-endocytosis hypothesis is not necessarily challenged by proteins affecting only one or the other. In fact this shows some of the relevance of the findings in this manuscript, e.g. that MyoF and KIC12 are stage-specific endocytosis proteins, leading to a better understanding of this process during blood stage development and shows the importance of stage-specificity on ART susceptibility. Besides stage-specific differences, we also have reason to believe RSA change may be a more sensitive measure of endocytosis than the bloated FV assay (see details in our response below) which may result in an observable RSA change without detecting an effect on endocytosis if the effect is not strong.

The reviewer's concern is mostly with 3 proteins. KIC4 and KIC5 that we previously localised to the K13 compartment (Birnbaum et al 2020) and MCA2 that was also analysed in that previous work but only here was localised, finding it at the K13 compartment. The factors influencing the correlation between endocytosis and RSA-changes mentioned above apply also to these proteins. In addition, the tools to study these proteins, technical issues - and for KIC4 dispensability and comparably low level of RSA change - makes them not the best examples to draw rigorous conclusions (this is also the reason why we originally did not attempt to do bloated food vacuole assays with these proteins in the previous work where we showed an endocytosis phenotype for five K13 compartment proteins). As outlined below the bloated FV assay to assess endocytosis - while an excellent indicator for an endocytosis effect if that effect is strong - is rather insensitive and so far has not shown clear effects in any protein not essential. MyoF, the protein mentioned by Reviewer 2 to show this should be possible, is an excellent example for this: its inactivation through tagging already causes an accumulation of endosomal vesicles (and a substantial growth defect) but only barely shows an effect in the bloated FV assay. Only further inactivation by KS leads to a clear effect. Hence, lack of a clear effect in the bloated assay should not be taken as unequivocal evidence for absence of endocytosis.

It is in our view not ideal to use the proteins with the weakest growth effect to test the RSA-endocytosis hypothesis and this was also not the original purpose of this work. Clearly there is the possibility that MCA2, KIC5 and KIC4 have a different function and that this is the cause of the changed RSA survival change, we already state this in the manuscript and now made changes to the text to better highlight this. However, as detailed in our response to the review below, we don't think an absolute statement can be made on that matter, both options are possible.

We now carefully reviewed the text to ensure this issue is treated in a balanced way and added the data the reviewer asked to be included into the manuscript. We here would like to stress that the most fundamental

message, that there is an overrepresentation of proteins with vesicle trafficking domains at the K13 compartment, is not affected by any of this. The domain analysis was done in an unbiased way and the number of vesicle trafficking domains found is by itself already quite striking (see details in our response below). That also all of these domains then fell into the group of proteins with endocytosis or RSA change phenotypes - even if we did not detect endocytosis functions for all of them - is rather unlikely to be a coincidence. Hence, the data in the manuscript, even with the question marks for KIC5 and MCA2 and unimportance of KIC4, supports the overall model quite well.

We also added a citation to a recent paper on KIC5 to the discussion.

We hope the revisions now satisfactorily addressed all the raised issues.

Reviewer's Responses to Questions
Part I - Summary
Please use this section to discuss strengths/weaknesses of study, novelty/significance, general execution and scholarship.

Reviewer #1: The revised version of the publication is much improved. Results are presented more clearly and structured. An enormous amount of data was generated and validated with solid experiments and the results will help the research community to further explore the endocytosis pathways in Plasmodium.
We thank the reviewer for the positive response.

Reviewer #2: This paper investigates putative K13 interaction partners, identified in a previous study. Of the 10 investigated, 4 are at the K13 compartment, and two of which are involved in endocytosis. One (KIC11) is likely involved in invasion. 3 other proteins identified previously as being involved in ART resistance (MCA2, KIC4 and KIC5) do not appear to be involved in endocytosis. The paper is novel and significant with overall adequate execution. Some of their lines are suboptimal (TGDs and the MyoF work due to the poor growth of the parasites and possible compensatory effects), however the authors do their best to address this. The weaknesses lie in some of the data interpretation and structural domain assignment, which suggests a lot of the K13 compartment proteins contain endocytic domains - however this is speculative and then the two candidates that they look at end up not being involved in endocytosis. I find it peculiar that some of the endocytosis data is not believed by the authors due to it not being supportive of their domain assignment or previous models.

We thank the reviewer for the careful consideration of the revised manuscript and alerting us to the perceived bias. The reviewer indicates that the structural domain data is speculative and that the proteins for which endocytosis function was assessed showed no effect. The latter point is part of the specific major point of this reviewer below and addressed there in detail. The domain point is not further elaborated below and we therefore address this here:

We firstly would like to emphasise that the domain analysis was done with all K13 compartment proteins we had after adding the newly identified proteins in this work, not only the newly found proteins that all came from the lower parts of the list. The previously identified K13 proteins had not been thoroughly analysed for domains, and alphafold had at the time not been available. Hence, it made sense to look the entire set of known K13 compartment proteins. Also of relevance, we did not make any subgroups of K13 compartment proteins based on the domains, it was the other way around (i.e. this was unbiased).

We are not sure why the reviewer thinks the domain assignment is speculative. The number of vesicular trafficking domains found in K13 compartment proteins is actually rather striking. Please note that we call them vesicular trafficking domains (title and elsewhere), not endocytosis domains and when appropriate specify that this includes domains typical for endocytosis (not all of these domains are specific for

endocytosis only). At the most basic level, the overrepresentation is independent of any subclassification of K13 compartment proteins or phenotypes obtained with these proteins: more than 30% of the confirmed K13 compartment proteins (6 of 17) have one or more vesicle trafficking domains and there are in total 13 such domains that were detected. For comparison, using a similar search with 931 proteins annotated as unknown in *P. falciparum,* we detected only 7 typical vesicle trafficking domains (that search detected at least one domain in 288 proteins and 326 domains in total, see our preprint: https://www.biorxiv.org/content/10.1101/2023.06.05.543710v2). The vesicle trafficking domains in these 931 proteins were:

1x RabGAP-TBC GTPase activator
1x GAT bind GGAs to Arfs
1x HOOK role in endocytic cargo sorting
1x Ist1 regulator of Bps4 activity of ESCRT
1x Syntaxin prototype family of SNARE proteins
1x Arf small GTPase
1x RSN1_TM includes first 3 TMs of 11-TM proteins involved in Golgi vesicle transport and exocytosis

This amounts to ~0.7% of all searched proteins. In total we found only about half as many such domains in the 931 proteins as in the 17 K13 compartment proteins and these domains were also less coherent in their supposed vesicular trafficking function. Taking into account some more generic domains that may be found in vesicle trafficking proteins (please note that all of the vesicle trafficking domains found in K13 compartment proteins were less generic than these) the 931 proteins also included:

9x C2 targets proteins to membranes
3x PH targets proteins to specific membranes.

With these added we end up with ~2% of all tested proteins (19 of 931 proteins with a total of 19 domains), still much less frequent than in K13 compartment proteins (6 of 17 proteins with a total of 13 domains). Clearly this search is not exactly comparable, but shows that random detection of finding such domains can be considered to be low and it is remarkable how many such domains are found in the experimentally confirmed K13 compartment proteins.

If we now do the subgrouping of placing all proteins for which we detected either an endocytosis or RSA change into one bin, we find that 6 of 11 proteins (over 50%) in that bin have such a domain. Or, all proteins with such domains fall into the group with the said phenotypes. One can now argue that our grouping is somewhat speculative because it is based on our previous hypothesis or - if going with the endocytosis hypothesis - one can argue that the domains falling into this group (in this unbiased search) is one more smoking gun for the hypothesis (other smoking guns are location at K13 compartment, influencing RSA, demonstrated endocytosis function, supportive data for similar function of orthologues/location at micropore from *Toxoplasma*, etc, see also below). In sum, most of these proteins are accumulating a lot of smoking guns even though each measure may have its imperfection and not everything will apply to all of the proteins.

Domains for the proteins the reviewer indicates do not fit (KIC4, KIC5 and MCA2): they are still K13 compartment proteins even without considering their falling into the RSA change group and therefore fit the most basic criterion to enter the domain search. Particularly for KIC4 and KIC5 the domains are far from speculative: these two proteins alone together contain 7 domains not only typical for general vesicle trafficking, but marking them as adaptors (hence, the domains fit a functional subtheme in vesicle trafficking, very unlikely to be a coincidence). By the similar approach mentioned above we found only one such domain (a GAT domain) in the over 900 *P. falciparum* proteins annotated as unknown. Overall we therefore believe the overrepresentation of proteins with vesicle trafficking functions is not speculative.

In order to illustrate the over-representation of vesicle trafficking domains found in K13 compartment proteins we now added a statement to the discussion of the manuscript: "A similar search with all un-

annotated *P. falciparum* proteins (in total more than 900 proteins) detected only 7 typical vesicle trafficking domains [108]. While this did not include annotated proteins, it nevertheless indicates that vesicle trafficking domains are overrepresented in the proteins found at the K13 compartment."

Reviewer #3: The manuscript by Schmidt et al. follows up on work that identified the molecular mechanism of ART resistance in P. falciparum (PMID: 31896710), by further investigating ten genes of the identified K13/Eps15-related “proxiome”. The authors link MCA2 to ART resistance in vitro, while the proteins MyoF and KIC12 are involved in endocytosis but do not confer in vitro ART resistance. Characterization of KIC11, which partially colocalizes with K13 in trophozoites/schizonts, indicates an important function in IDC unrelated to endocytosis. Five analyzed genes however do not colocalize with the K13 compartment, while a sixth was refractory to endogenous tagging. Using AlphaFold prediction the authors identify protein domains in K13 compartment constituents, which have not been recognized before due to their unusual arrangement and low level of primary sequence conservation.
Endocytosis is insufficiently understood in Plasmodium and this manuscript makes an important contribution by further dissecting the unusual protein machinery employed by the parasite. Overall this study is of high quality and the presented experiments are well controlled for. The authors adequately addressed my previous comments and I only have some small text edit suggestions.
We thank the reviewer for the positive response and for the suggested text edits.

Part II – Major Issues: Key Experiments Required for Acceptance
Please use this section to detail the key new experiments or modifications of existing experiments that should be absolutely required to validate study conclusions.

Generally, there should be no more than 3 such required experiments or major modifications for a "Major Revision" recommendation. If more than 3 experiments are necessary to validate the study conclusions, then you are encouraged to recommend "Reject".

Reviewer #1: No major issues in the revised publication.
Reviewer #2: First of all I would like to thank the authors for all the extra work that they have done for the manuscript. It has improved the overall quality and accessibility of the data.
We thank the reviewer for this assessment.

However, I am concerned by the authors' resistance in believing the endocytosis assay data for MCA2, KIC4 and KIC5 TGD lines. It appears that the correct controls have been included and that there are no endocytosis defects. It could be argued that the data on KIC5 is questionable because of the major growth defect of this TGD line and the inability to knock the protein sideways, but MCA2 and KIC4 do not have growth defects, so how can the authors still not support this data? I also find it particularly concerning that despite the fact that they generated a convincing KIC4 KS line , they still chose not to believe the fact that KIC4 is not involved in endocytosis and chose not to put the data in the paper all together. Is this because it weakens their structural domain prediction data or is it because the MCA2 and KIC4 data does not support the model put forward by Birnbaum et al., 2020 paper that links the endoytosis/ART resistance link? Moreover if the authors don't believe some of their data because their TGD lines have growth defects, why do they believe the MyoF data? This is a major contradiction and extremely concerning and could be evidence of cherry picking data. I strongly urge the authors to include the KIC4 KS data in the manuscript and highlight how an ART resistance protein might not be involved in endocytosis (as is the case for MCA2 truncated line and KIC4) where both show ART resistance and no involvement in endocytosis.

There are several different issues in this comment. As this includes suspected misinterpretation and cherry picking of data we address these aspects in detail (we apologise that this resulted in a rather lengthy response).

Bias/cherry picking/hiding data because of not fitting hypothesis:

Firstly, we would like to stress that we were not influenced by worries about the endocytosis hypothesis or the fit with the conclusions drawn in this manuscript (we do not think there are reasons for that, see below and response to editor). But there are limitations of our assay that we feel we have a responsibility to let the reader know. We can't unequivocally exclude the possibility that KIC5 and/or MCA2 can influence endocytosis because the data is not strong enough to draw such a conclusion (see detailed discussion on KIC5 and MCA2 in the specific section below). If someone later on finds some influence of these proteins on endocytosis, we would like to avoid that the phrasing of our manuscript contradicts new, stronger data. We would also like to mention that we did not write in the manuscript that we do not believe these results, we simply highlighted factors relevant to assess these experiments (technical, stage-specificity) in addition to the possibility of a differing function causing increased RSA survival (which we do state). We went over the manuscript and amended the text to ensure this is written in a balanced fashion and hope this is now acceptable. The reviewer has a valid point asking why MyoF can show an effect in the bloated FV assay and this is addressed below in the part discussing KIC5 and MCA2. Irrespective of the endocytosis issue, KIC4 and KIC5 fit the most basic message of the manuscript that proteins containing vesicle trafficking domains are overrepresented at the K13 compartment, hence the most general conclusion (the one in the title) is unaffected by RSA or endocytosis data. Please note that KIC4 is dispensable for parasite proliferation, therefore unlikely to have an important function in endocytosis or any other essential process in the first place (see specific discussion on KIC4 below). It is noteworthy that also KIC5 and MCA2 are partially dispensable for *in vitro* growth and so far we were only able to detect an endocytosis phenotype by bloated assay for "essential" proteins. At present we do not know if the assay is sensitive enough to see endocytosis defects that lead to parasite growth reduction to a degree that it still permit deletion of the corresponding gene (again see considerations for KIC5/MCA2 below).

Secondly, we had no intention to hide data, we already published the data in question in the rebuttal that appeared together with the re-submission of the revised pre-print on bioRxiv (https://www.biorxiv.org/content/10.1101/2022.12.15.520209v3#review). We only realised when uploading that the figures in the rebuttal were not permitted to be included (only the rebuttal text - including the figure legend - is published; maybe this is something that could be taken up with Review Commons and bioRxives to be improved in the future). However, the published text already states what we found and hence it is publicly available. While the lack of the rebuttal figures is not optimal, we expect them to be published if this manuscript becomes accepted (or if bioRxiv changes this feature for the rebuttal in which case we would gladly update it). Also, if we wanted to hide this, why place it in the rebuttal? We were not specifically asked for a KS line (see section on KIC4 below which also explains why we still made the KS line).

Thirdly, the reason we showed the KIC4 KS data only in the rebuttal but not the manuscript was because we were asked by the reviewers to streamline the manuscript and concentrate on results that move the paper forward. The KIC4 KS does not add much, it confirms the KIC4 TGD results that the protein is dispensable for growth (Birnbaum et al., 2020) and endocytosis (data added to this manuscript in the previous revision).

Fourth, we have no issues with adding the KIC4 KS data to the manuscript apart from it not showing much and now did this as new Figure S10.

Considerations for KIC4:

We conducted the KIC4-TGD bloated FV assay because we were asked by the reviewer to do it (KIC4 originally was only relevant in the final part of the manuscript that looked at the domains). However, the KIC4-TGD has no growth defect (which we would expect if it affected endocytosis to an extent it can be seen in a bloated FV assay; so far all proteins leading to a bloated FV phenotype in trophozoites were refractory

for disruption) and the RSA survival of the TGD was just only above 1%. We are aware this is an imperfect comparison, but Kelch13 variants leading to a very minor K13 abundance reduction in ring stages (assuming an abundance-function relationship), result in ~ 1% RSA survival (doi: https://doi.org/10.1101/2022.05.13.491767). Hence, a small reduction in endocytosis in rings can lead to a detectable RSA survival change. Based on these considerations there are two possible explanations why our bloated assay does not show an effect for endocytosis but a change in RSA survival:

(i) RSA is a finer measure that is capable to see small endocytosis reduction in rings that we can't see with the bloated food vacuole assay in trophozoites or
(ii) that KIC4 reduces RSA susceptibility due to a different (but still dispensable) function.

It is also possible that KIC4 reduces endocytosis only in rings (like Kelch13 which does not affect bloating nor growth in trophozoites when inactivated, although for KIC4 this would be at a much lower level than for K13).

What does the KIC4 knock sideways data (the experiments the reviewer indicates we should not have omitted from the manuscript) add to this? While clearly a knock sideways is much better suited to assess endocytosis phenotypes, in this case there is no growth defect, hence the TGD parasites likely are not under selection to adapt (or at least to a lower extent) and stage-difference issues (a problem with assays using TGDs) will be less of a problem as the parasites will grow similarly. Hence, if there is no effect in the TGD on growth or endocytosis, the KS probably will also not show anything. In the case of KIC4 we also knew that a possible effect on endocytosis likely would be small, as the RSA change was just above the detection limit. We nevertheless generated the KIC4 knock sideways line and did this experiment to have tried everything possible, knowing from the start that due to the dispensability of KIC4 the probability it would show something was very small (but one never knows, there are always unknown factors). As this was not the case (and confirmed the TGD), we did not consider it worthy to include into the manuscript but placed it into the published rebuttal (see above why the actual figure is missing there). This has now been changed and the KIC4 KS was included as Figure S10.

Does the absence of effect in the bloated assay with KIC4 challenge the endocytosis hypothesis and give reasons for the suspected bias? KIC4 is dispensable and therefore it can't have an important role in endocytosis, but also no other role important for growth of asexual blood stages. Does this mean it could not in the ring reduce endocytosis to a small degree, explaining the RSA result if we assume the two things are connected? We are of the opinion that this option can't and shouldn't be discarded. Similarly, it can't be excluded that the KIC4 TGD reduced endocytosis only in rings (again, likely not by much, given the 1% RSA survival). Clearly we also can't exclude that a different function leads to the reduced RSA survival. But resolving this question is not central to this manuscript and in our opinion a non-essential proteins with a just above threshold RSA change is also not the ideal protein to make a general statement about the endocytosis-RSA hypothesis. The moderate endocytosis reduction expected (maybe restricted to rings) - if the hypothesis is correct - will be very difficult to measure. We therefore leave it at mentioning the two possible reasons for the RSA change with KIC4 (i.e. different function or change in endocytosis is too low/restricted to rings). Of note, KIC4 is in the the pool of proteins associated with an RSA change because of a survival of the TGD parasites above the threshold. But even if placed in the other group, it would not change the general enrichment of vesicle trafficking domains at the K13 compartment (please also note that the other group could in principle contain redundant endocytosis proteins).

Biased view on KIC4 due to our previous hypothesis: How does "KIC4 has a different dispensable function leading to the reduced RSA survival" compare to "the low RSA survival is too small or stage-specific to lead to a measurable endocytosis reduction" if we look at everything as it stands now? Please note that apart from the K13 co-localisation the following considerations were not used for the grouping, as they take the domain

finding already into account. KIC4 has four domains highlighting it as an adaptor (clearly showing it is a vesicle trafficking protein, not necessarily endocytosis) of which two domains were also detected by other means (not using alphafold structure comparison) in the *Toxoplasma* orthologue TgKAE (Koreny et al. 2023). KIC4 is located at the neck of the cytostome (KIC4) in *P. falciparum* and at the micropore (KAE) in *Toxoplasma gondii* (note that a pre-print now confirms the K13 rings to correspond to the cytostomal neck, Tutor et al. 2023). In Toxoplasma KAE is essential (maybe because KIC5 is missing in this parasite?) (Koreny et al. 2023). Structural modelling indicated the KAE ear domain (one of the typical adaptor domains) can bind EPS15 and this was confirmed with a recombinant version of TgKAE that bound TgEPS15 in a WxxF motif dependent manner (the expected binding motif). TgEPS15 co-locates at the micropore with TgKelch13 and in *P. falciparum* EPS15 was shown to bind PfKelch13 (by Co-IP) and to have a function in endocytosis (Koreny et al. 2023, Birnbaum et al. 2020). While all of this does not exclude a different function of KIC4, the probability that it has a different function that also influences RSA survival but differs from that of the other proteins it co-locaises with and even interacts with seems at least not highly probable. Therefore, we currently favour the possibility that KIC4 is a redundant protein at the K13 compartment that in rings might have a small effect on endocytosis, resulting in the RSA change. Also, given the 4 domains marking it as an adaptor, if it has a different function than endocytosis, this would very likely be in a different type of vesicle trafficking process. Again, this would be rather unusual to take place at the same site (the cytostome) where endocytosis is thought to take place. Clearly, the "other function" hypothesis should not be excluded, but at present it seems to us this is the less likely option. However, both of these options are in the manuscript and we amended the text to give what we hope is a balanced view (lines 666 - 675 in the track change version of the manuscript).

Dispensability of MCA2:

The reviewer indicates that MCA2 had no growth defect. However, the MCA2-TGD does have a growth defect. The reviewer is probably referring to the $MCA2^{Y1344stop}$ line which has a truncation removing ~1/3 of the protein and which does not result in a growth defect but still has a detectable effect on ART susceptibility (average of ~1.5% RSA survival) - although smaller than the MCA2-TGD. $MCA2^{Y1344stop}$ therefore does not impair the main function of MCA2, but can be expected to behave similarly to KIC4 (no growth defect, hence no detectable effect on endocytosis, small but significant effect on RSA survival, raising the similar range of options as for KIC4). In contrast, the MCA2-TGD line has a growth defect (Birnbaum et al. 2020) and behaves similarly to KIC5 in that it leads to a reduced FV size in the bloated assay that however is accompanied also with a smaller parasite size (see next section).

MCA2 and KIC5 TGD lines compared to similarly inactivated MyoF:

The reviewer indicates that the MyoF-sandwich-tag line has a growth defect and this should therefore be comparable in the endocytosis assay to KIC5 and MCA2. However, the MyoF-sandwich line still showed 88% of bloated food vacuoles, indicating that despite the considerable reduction in growth (~37% growth in a 2 cycle assay), the effect was just marginally detectable by this assay and we would not have dared concluding anything on this result alone (see Fig. 1L). Of note, in this MyoF line vesicles filled with hemoglobin are present (average of ~5 per parasite), clearly showing that transport of hemoglobin to the FV is reduced and hence shows that the bloated assay unfortunately is not very sensitive to detect what seems to be quite a substantial reduction in endocytosis. Only the knock sideways that reduced growth by another ~40% (in sum this then corresponds to only ~20% growth compared to 3D7) led to a stronger effect with only ~50% of the cells still showing a bloated FV and a proportionally smaller FV in the refined assay. Due to the limited sensitivity some influence on endocytosis can therefore not be categorically excluded with this assay if the effect is not very strong (so far we did not see a significant effect with a protein for which a TGD was possible).

The refined assay looking at parasite vs FV size probably is more sensitive but MyoF is not fully comparable with MCA2 and KIC5 for several reasons:
- the refined assay was done with the knock sideways.
- the parasite size reduction due to endocytosis reduction is expected to be more profound if hemoglobin does not even enter the parasite (as is the case with inactivation causing a block in early phase of endocytosis such as seen for UBP1, EPS15, AP2u, KIC12 and KIC7) compared to a block during transport of hemoglobin seen for MyoF which is still internalised into the parasite (contributing to cell size). No vesicles are apparent in the KIC5 and MCA2 TGDs making it more likely parasite size is affected proportionally to FV size in case endocytosis was negatively influenced. In contrast, for MyoF, parasite size will be less affected and the disproportional effect of FV vs parasite size can be expected to be more profound.
- a fine comparisons of parasite and FV size works best in conditional lines where the same culture can be split at the starting point, which may be contributing to the detectability of the effect in the MyoF KS line.
- it also has to be considered that in contrast to MyoF, KIC5 and MCA2 likely are also important in rings (something that could be assumed due to the changed RSA survival if the hypothesis is correct) and hence have a much longer time to amount a growth difference when starting with the synchronised rings compared to the 3D7 control (bloating starts only in the troph stage).

Overall, low sensitivity of the bloating assay, stage differences and differences in the nature of the phenotype are reasons why the KIC5 and MCA2 bloated assay is not fully comparable with MyoF. Importantly, inactivation of MyoF by tagging alone also had only a minor effect in the bloated assay. For these reasons this assay can't categorically exclude KIC5 and MCA2 to have some contribution to endocytosis in rings that could explain the RSA change. The bloated assay is an excellent indicator for an effect on endocytosis if the reduction is very strong (essential endocytosis proteins) but it might miss smaller effects on endocytosis. Thus, we are somewhat hesitant to state KIC5 and MCA2 have a different function in an absolute way without alerting to some of the potential limitations of the data.

We added an additional statement to the discussion to enforce that there are two possibilities for the results with the proteins for which we were able to obtain a TGD and have an effect in RSA: "We conclude that either these proteins influence ART susceptibility through a different function than endocytosis or that technical limitations or stage-specificity led to a failure to detect a contribution of these proteins to endocytosis."

I would consider re-naming the paper - only 2 of the 12 candidates you looked at are involved in endocytosis or the K13 compartment. Maybe consider something along the lines of divergent functions of K13 compartment proteins including proliferation and endocytosis.

We see the reviewer's point to change the title if this is viewed from the 4 (if we include MCA2 which was previously analysed but not localised) newly discovered K13 compartment proteins in this manuscript. But this was done to obtain a more complete list of K13 compartment proteins followed by an analysis of all K13 compartment proteins we had after that. As this was the final goal of the work, we would prefer having a title with a conclusion on the sum of K13 compartment proteins. A title making a general statement about the K13 compartment based on the bottom part of the list would not be a faithful representation of that compartment. As indicated above, looking at this "completed" (as much as we could) list of K13 compartment protein showed an overrepresentation of proteins with vesicle trafficking domains (again please note that we do not call them endocytosis domains) and we therefore believe it is a reasonable representation of the final conclusion of this manuscript.

The reviewer suggests including "diverse functions" of K13 compartment proteins into the title. Looking at the experimentally confirmed functions, we think this is somewhat speculative and hence do not feel

comfortable adding this to the title. For dispensable proteins we have no detectable phenotype, hence we do not know their function and also can't exclude that they are redundant endocytosis proteins. Only KIC11 clearly has a different function but as stated in the manuscript, it is possible this is due to KIC11 foci that do not overlap with K13 in schizonts (therefore not necessarily indicating more than one function at the K13 compartment which might be implied if this is included in the title). MCA2 and KIC5 may have other functions than endocytosis but this we do not know with absolute certainty (see above) and we also do not know what this different function could be (apart from KIC5 showing strong hallmarks of a vesicle adaptor). In summary this leaves us with:

- for the endocytosis/RSA change group: 7 proteins experimentally linked to endocytosis (all important for growth), 1 dispensable, 2 with partial importance for growth that may have other unknown function than endocytosis
- for the group of proteins without previous phenotype: 1 essential protein likely needed for invasion and 6 dispensable proteins.

We now clarified in the manuscript that the conclusions possible to draw about dispensable proteins are limited. This applies to KIC4, but also reflects on the other group of proteins.

The enrichment of vesicle trafficking domain proteins is a more conservative conclusion. Maybe calling it a hub of such proteins is an overstatement as despite the overrepresentation, there are proteins without such a domain. We would also like to stress that the proteins with vesicle trafficking domains show unusual features (compared to those in model organisms) and hence it is important to state that they are divergent, even in proteins such as KIC4 and KIC5 that have multiple vesicle trafficking domains typical for a very specific functional group (vesicle adaptors). Taking all these considerations into account we renamed the paper to: "The Kelch13 compartment contains highly divergent vesicle trafficking proteins in malaria parasites". We hope this satisfies the issues raised in regards to the suggested title change, as the new title does less exclude the possibility that other proteins or functions are present at the K13 compartment or that individual K13 compartment proteins can have other functions.

Figure 1C: the merozoite image is not representative of true merozoites, rather a schizont that is beginning to rupture. Please amend of the label of this subpanel or change the image to individual merozoites.
The label was changed to rupturing schizont.

Line 250: the authors state here 'speaking against other myosins taking over the MyoF endocytosis function in rings'. This suggests MyoF is involved in endocytosing in rings - when the authors have not shown that MyoF is acting in rings, how can the authors state this? Especially considering MyoF is not involved in ART resistance how can the authors make these claims?
We apologise for the clumsy wording, this is not what we meant with this sentence. What we intended to say is that while MyoF is not involved in endocytosis in rings (as pointed out by the reviewer and also clearly stated in the manuscript apart from this unwise wording), this does not per se exclude actin playing a role in endocytosis in rings (e.g. if a different Myosin than MyoF were involved). The cytochalasinD experiment however speaks against this option, as it suggests actin is not needed at all. We have reworded this to: "...indicating an actin/myosin independent endocytosis pathway in ring stage parasites (Figure 1M) and also speaking against a function of other myosins in endocytosis in rings."

Many of the figures, namely figures 1 & 2 are very difficult to follow since the figures are not in order and jump up and down the page in terms of order. Please revise this so that it is easier for the reader to follow the order of the figures.
The order of the figure panels in Fig. 1 and 2 was re-arranged to make them more chronological.

Reviewer #3: none

Part III – Minor Issues: Editorial and Data Presentation Modifications
Please use this section for editorial suggestions as well as relatively minor modifications of existing data that would enhance clarity.

Reviewer #1: No minor issues in the revised publication.
Reviewer #2: Line 59: typo - Papua New Guinea not Papa New Guinea
We thank the reviewer for pointing this out, this typo was corrected.

Reviewer #3: Edits:
-line 59: Change Papa New Guinea to Papua New Guinea
We thank the reviewer for pointing this out, this typo was corrected.

-line 149: Change location to localization
This was corrected.

-line 350: Change location to localization
This was corrected.

-line 435-436: "….verified by Western blot…" should be "verified by western blot…"
Please refer to previous rebuttal, both versions seem to be accepted and for instance Nature and Science have differing policies in regards to this. We will be happy to change this if at odds with what is customary for Plos Pathogens.

-line 1056: Means of independent experiments are presented as colored dots in Fig 1J and not as triangles as stated in the legend, please adjust either text or figure.
We thank the reviewer for pointing this out, the text was changed.

-line 1158: Same as above, please adjust for Fig 3G/I
We thank the reviewer for pointing this out, the text was changed.

-line 1185: Please fix typo in metacaspase
We thank the reviewer for pointing this out, this was corrected.

PLOS authors have the option to publish the peer review history of their article (what does this mean?). If published, this will include your full peer review and any attached files.

If you choose "no", your identity will remain anonymous but your review may still be made public.

Do you want your identity to be public for this peer review? For information about this choice, including consent withdrawal, please see our Privacy Policy.
Reviewer #1: No
Reviewer #2: No
Reviewer #3: No
